# Hydrolyzed corn starch with maltotetraose for skin defense through NRF2 pathway activation in human keratinocytes

**DOI:** 10.1371/journal.pone.0351422

**Published:** 2026-06-26

**Authors:** Hitomi Hagawa, Eri Ichikawa, Keiko Otake, Mayuko Ishii, Naoko Kawaguchi

**Affiliations:** 1 Global Development Center, Research & Development Headquarters, Lion Corporation, Edogawa-ku, Tokyo, Japan; 2 Northeast Asia Business Division, Business Development Department 1, Lion Corporation, Taito-ku, Tokyo, Japan; Helwan University, EGYPT

## Abstract

Oxidative stress, which can be triggered by various external stimuli, such as ultraviolet radiation and pollution, compromises skin health, accelerates aging and skin disorders, and impacts quality of life. This study investigated the potential of hydrolyzed corn starch containing maltotetraose in skin defense through nuclear factor erythroid 2-related factor 2 (NRF2) pathway activation in human keratinocytes. Using DNA microarray analysis and assessing key antioxidant-responsive genes, we evaluated the capacity of this ingredient to enhance cellular defense mechanisms. Human keratinocytes treated with it demonstrated significant upregulation of antioxidant-responsive genes, including *HMOX1*, *GPX2*, and *NQO1*, which are known targets of NRF2. These findings were corroborated by western blotting and immunostaining analyses, which confirmed increased NRF2 protein expression and NRF2 nuclear translocation. Moreover, fluorescence assays and microscopy showed that treatment with the ingredient effectively reduced reactive oxygen species levels in keratinocytes exposed to oxidative stress. These results suggest that activation of the NRF2 pathway by this ingredient enhances the cellular antioxidative response and reduces reactive oxygen species levels in keratinocytes. This NRF2-mediated antioxidative activity positions hydrolyzed corn starch containing maltotetraose as a promising ingredient for cosmetic products, with potential implications for improving skin health, reducing pigmentation, and combating aging. However, these effects were not directly evaluated in this study. Future studies are warranted to evaluate its efficacy *in vivo.*

## Introduction

The skin serves as the interface between the external and internal environments and is continuously exposed to various external stimuli, including ultraviolet radiation, air pollution, tobacco smoke, and alcohol [1–3]; these stimuli generate reactive oxygen species (ROS). Excessive or prolonged exposure to ROS leads to oxidative stress [4], which in turn exacerbates skin pigmentation and aging, ultimately resulting in uneven skin tone, hyperpigmentation, roughness, sagging, and wrinkles. This may lead to cosmetic imperfections and progress to severe dryness, sensitivity, and more severe conditions, such as atopic dermatitis, psoriasis, and skin cancer, thereby significantly impacting quality of life. Considering that the skin is continuously subjected to oxidative stress, potent antioxidant defenses are essential, especially for low-barrier or sensitive skin, which is particularly susceptible to external stimuli.

**Fig 1:**
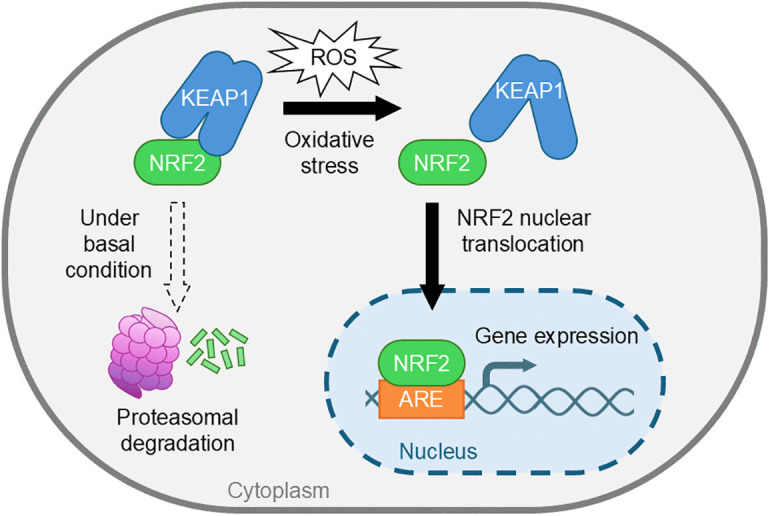
NRF2-Keap1 pathway under basal and oxidative stress conditions.

The skin possesses a robust antioxidant defense system, with the transcription factor nuclear factor erythroid 2-related factor 2 (NRF2) playing a central role by regulating the expression of a wide array of antioxidant enzymes responsible for detoxifying and eliminating oxidative agents. Under basal conditions, NRF2 is maintained in an inactive state in the cytoplasm through its binding to Kelch-like ECH-associated protein 1 (Keap1) and is directed toward proteasomal degradation. Under oxidative stress, NRF2 is released from Keap1 and translocates into the nucleus, where it binds to the antioxidant response element (ARE). This interaction modulates key skin defense mechanisms, including phase II detoxification, the regulation of inflammatory responses, DNA repair, and antioxidant responses (Fig 1) [5,6]. In the skin, the NRF2 pathway has been implicated in photoprotection, regulation of pigmentation, wound healing, and reinforcement of the epidermal barrier function [3,7]. From a cosmetic perspective, enhancement of NRF2 signaling is therefore expected to strengthen endogenous antioxidant and barrier defenses, thereby helping to prevent photoaging, pigmentation disorders, and barrier impairment induced by environmental stimuli. Moreover, transient NRF2 activation is unlikely to be harmful, as NRF2 upregulates Keap1 expression via a negative feedback mechanism that contributes to the maintenance of physiological redox balance in cells [3]. Consequently, incorporating NRF2 modulators into cosmetic formulations can be viewed as a promising strategy to protect the skin from environmental stress and to maintain skin homeostasis.

Hydrolyzed corn starch is officially categorized as a skin conditioning agent, specifically a humectant [8]. We have identified hydrolyzed corn starch, derived from corn, as a safe and effective naturally derived functional cosmetic ingredient. This starch mainly contains maltotetraose (MTO), a polymer of glucose linked by α-1,4-glycosidic bond. Given its higher concentration of hydroxyl groups compared to other humectants, such as glycerin and butylene glycol, MTO has potentially enhanced moisturizing capacity. Moisturization is a fundamental aspect of skin care; for low-barrier and sensitive skin, application of moisturizers is crucial for restoring the skin barrier [9]. In addition, the itch–scratch cycle, observed in sensitive skin, can significantly impair quality of life. Our previous studies have demonstrated that MTO possesses notable itch-suppressing effects. Specifically, MTO significantly suppresses chronic itching by modulating mechanisms involving substance P and the neurokinin 1 receptor (NK1R) [10,11], notably through inhibition of NK1R activation [12]. Based on these findings, we developed an itch-relief moisturizer containing MTO. A clinical trial involving patients with dry, itchy, and sensitive skin demonstrated that treatment with MTO effectively moisturized the skin, enhanced the skin barrier, and reduced itching [13]. Therefore, MTO holds promise as a functional component in the cosmetic field.

In this study, we aimed to investigate the other potential applications of MTO as a cosmetic ingredient by exploring its novel functionalities using human epidermal skin cells. Specifically, we examined the possibility of enhancing the skin’s ability to defend against oxidative stress by activating NRF2 and reducing ROS.

## Materials and methods

### Ethics statement

Normal Human Epidermal Keratinocytes (NHEKs; adult, single donor) were purchased from PromoCell GmbH (Heidelberg, Germany; Cat. No. C-12003; Lot Nos. 489Z018.2, 470Z025.1, 479Z032.1). PromoCell obtains donor tissues only after written informed consent and in accordance with the Declaration of Helsinki, providing fully anonymized samples (https://www.promocell.com/ethics). Under Lion Corporation Institutional Review Board (IRB) policy, research readily obtainable from commercial sources is exempt from additional review.

### Cell culture

NHEKs (PromoCell, Cat. No. C-12003) were purchased from Takara Bio Inc. (Shiga, Japan) and cultured in Keratinocyte Growth Medium 2 (PromoCell, Cat. No. C-20011) containing 5 μg/mL insulin, 0.125 ng/mL human recombinant epidermal growth factor, 0.33 μg/mL hydrocortisone, 0.39 μg/mL epinephrine, 10 μg/mL holo-transferrin, 0.06 mM CaCl_2_ and 0.004 ml/mL bovine pituitary extract at 37°C in a humidified atmosphere of 5% CO_2_. Hydrolyzed corn starch (Leogard MT; Lion Specialty Chemicals Co., Ltd., Tokyo, Japan) was added to dissolve in the medium with NHEKs.

### DNA microarray analysis

NHEKs were seeded and grown in six-well plates until they were subconfluent in a 37 ℃, 5% CO_2_ incubator. Then, the control samples (only the medium) and 2.0% hydrolyzed corn starch in the medium were added, and after culture for 24 h, RNeasy Plus Mini Kit (Qiagen, Hilden, Germany) was used to extract RNA from the cells following the manufacturer’s protocol. The quality and quantity of the total RNA were evaluated on an Agilent 2100 Bioanalyzer (Agilent Technologies Japan, Ltd., Tokyo, Japan) and the total RNA integrity number exceeded seven. These total RNA samples were subjected to DNA microarray analysis using a Clariom^TM^ S Array, human (Thermo Fisher Scientific Inc., MA, USA) by Kurabo (Osaka, Japan), and the fluorescence signals were scanned with a Biosystems^TM^ GeneChip Scanner 3000 7G System. Biosystems^TM^ GeneChip Command Console Software ver. 3.2 was used to reduce the array images to the intensity values for each probe (CEL files).

### DNA microarray data analysis

Using R software (ver. 4.3.2; The R Project, https://www.r-project.org/), the CEL files obtained from three independent samples per condition (n = 3) were quantified according to the distribution Robust Multichip Analysis. A statistical comparison of the control and 2.0% hydrolyzed corn starch was performed with the Rank Products and Benjamini and Hochberg false discovery rate (FDR) corrections. The Human Genome Array annotation file was used (Clariom™ S Array, human Transcript Cluster Annotations, CSV, Release 36). Differentially expressed genes were extracted on setting the values of *p* < 0.15, and the Database for Annotation, Visualization, and Integrated Discovery were used to classify the selected probe sets according to functional annotations. The functional analysis was performed on the basis of the molecular function in UniProtKB(UP)_Keywords(KW)_MF. In addition, while comparing cells treated with 2.0% hydrolyzed corn starch and control cells, genes that exhibited signal intensities of 20% to 100% with a fold change of ≥2.0 were considered upregulated. A significant difference was defined as an FDR-corrected value of *p* < 0.05.

### Gene-expression analysis

NHEKs were seeded and grown in six-well plates until they were subconfluent in a 37 ℃, 5% CO_2_ incubator. Then, the samples of control and 1.0%, 2.0%, and 4.0% hydrolyzed corn starch were added, and after culturing for 3, 6, 10, 24, and 48 h, an RNeasy Plus Mini Kit (Qiagen) was used to extract RNA from the cells following the manufacturer’s protocol. Then, the One Step TB Green® PrimeScript™ RT-PCR Kit II (Takara Bio) was used to evaluate the mRNA levels of heme oxygenase 1 (*HMOX1*), glutathione peroxidase 2 (*GPX2*), NAD(P)H: quinone oxidoreductase 1 (*NQO1*), peroxiredoxin 1 (*PRDX1*), superoxide dismutase 1 (*SOD1*), catalase (*CAT*), glutathione reductase (*GSR*), glutamate-cysteine ligase catalytic subunit (*GCLC*), thioredoxin (*TXN*), and glyceraldehyde 3-phosphate dehydrogenase (*GAPDH*). The primer sequences were as follows: human *HMOX1* forward, 5′-tcctgctcaacatccagctc-3′; human *HMOX1* reverse, 5′-cacatggcataaagccctac-3′; human *GPX2* forward, 5′-ttgatatcagtccccttcactg-3′; human *GPX2* reverse, 5′-ctgccctttattggtctcttc-3′; human *NQO1* forward, 5′-agtcatctcattccactgttgg-3′; human *NQO1* reverse, 5′-catctggtaaaggaggttttcc-3′; human *PRDX1* forward, 5′-gccaagtgattggtgcttc-3′; human *PRDX1* reverse, 5′-aatggtgcgcttcgggtc-3′; human *SOD1* forward, 5′-ctcactctcaggagaccattg-3′; human *SOD1* reverse, 5′-cacaagccaaacgacttccag-3′; human *CAT* forward, 5′-tgcggagattcaacactgc-3′; human *CAT* reverse, 5′-ggcaatgttctcacacagac-3′; human *GSR* forward, 5′-tatgtgagccgcctgaatg-3′; human *GSR* reverse, 5′-ctgacctctattgtgggcttg-3′; human *GCLC* forward, 5′-gaagtggatgtggacaccag-3′; human *GCLC* reverse, 5′-cttgtagtcaggatggtttgc-3′; human *TXN* forward, 5′-gtagttgacttctcagccacg-3′; human *TXN* reverse, 5′-ctgacagtcatccacatctacttc-3′; human *GAPDH* forward, 5′-tgacaacgaatttggctacag-3′; and human *GAPDH* reverse, 5′- agggtctctctcttcctcttg-3′. Three replicate wells were evaluated for each group (n = 3).

### Evaluations of the ROS levels

The ROS assay was performed according to the manufacturer’s protocol for the ROS Assay Kit Photo-oxidation Resistant DCFH-DA (DOJINDO, Kyushu, Japan), a redox-sensitive dye that fluoresces after being oxidized by ROS. NHEKs were seeded into 96-well plates at a density of 7 × 10^4^ cells/well. After culturing for 24 or 48 h at 37 ℃ in a 5% CO_2_ incubator, the samples of control, 2.0%, and 4.0% hydrolyzed corn starch were added. The cells were washed twice with Hank’s buffered salt solution (HBSS), and then the ROS detection fluorescent reagent was added for 30 min in a 37 ℃, 5% CO_2_ incubator. Subsequently, the cells were washed twice with HBSS, then 200 μM H_2_O_2_ diluted in HBSS was added, and the cells were reacted for 1 h in a 37 ℃, 5% CO_2_ incubator. The cells were then washed twice with HBSS, and a fluorescence microplate reader was used to measure the ROS levels. Three replicate wells were evaluated for each group (n = 3).

### Western blotting

NHEKs were seeded and grown in six-well plates until they were subconfluent in a 37 ℃, 5% CO_2_ incubator. Then, the samples of the control and hydrolyzed corn starch were added after culturing for a specified time. The cell suspension was collected for NRF2 detection by separately extracting the nuclear and cytoplasmic proteins according to the NE-PER™ Nuclear and Cytoplasmic Extraction Reagents (Thermo Fisher Scientific) protocol, followed by protein quantification with the bicinchoninic acid protein assay kit (Takara Bio).

Equal concentrations of protein samples were diluted in Novex Tris-Glycine Sodium Dodecyl Sulfate Sample Buffer (Invitrogen Corp., Carlsbad, California, USA) and NuPAGE Sample Reducing Agent (Invitrogen) followed by heating at 85 ℃ for 3 min to denature the proteins. Then, the protein samples were subjected to sodium dodecyl sulfate-polyacrylamide gel electrophoresis and transferred to polyvinylidene difluoride membranes (Bio-Rad Laboratories, Inc., California USA). The membrane was blocked with 5% skim milk in Tris-buffered saline and polysorbate 20 (FUJIFILM Wako Pure Chemical Corp., Osaka, Japan) for 1 h at room temperature, and the corresponding primary antibodies: β-actin (Cell Signaling Technology, Inc., Danvers, MA, USA) at a 1:5000 dilution and NRF2 (Abcam, Cambridge, UK) at a 1:500 dilution were incubated overnight at 4°C. The next day, the polyvinylidene difluoride membranes were washed three times with Tris-buffered saline containing polysorbate 20 and incubated with the corresponding rabbit antibody (Abcam; 1:20,000 dilution for NRF2 and 1:100,000 dilution for β-actin) for 1 h at room temperature. The target protein bands were detected with a chemiluminescence method using the ECL select western blotting detection reagent (Cytiva, Tokyo, Japan) and Amersham ImageQuant 800 (Cytiva). The target protein was quantified using ImageJ (National Institutes of Health, Bethesda, MD, USA), and each protein amount was normalized to that of β-actin. Three independent samples were evaluated for each group (n = 3).

### Immunostaining

NHEKs were seeded and grown in six-well plates until they were subconfluent in a 37 ℃, 5% CO_2_ incubator. Then, the samples of the control and 4.0% hydrolyzed corn starch were added. After culturing for 2 h, the cell supernatant was removed, and then 1 mL/well of 4% (w/v) formaldehyde (Thermo Fisher Scientific) solution dissolved in phosphate-buffered saline (PBS) was added and incubated at room temperature for 15 min. Then, after washing three times with PBS, 1 mL/well of 0.5% Triton X-100 (Sigma Aldrich, St. Louis, Missouri, USA) solution dissolved in PBS was added followed by incubation at room temperature for 15 min. After washing three times with PBS again, 2 mL/well of 3% bovine serum albumin (FUJIFILM Wako Pure Chemical Corp.) solution dissolved in PBS was added for blocking at room temperature for 1 h. Then, the primary antibody (Proteintech Group, Inc., Rosemont, IL, USA; dilution concentration 1:250) was incubated overnight at 4°C. The next day, after washing three times with PBS, a secondary antibody (dilution concentration 1:300) was added and incubated at room temperature for 30 min. After washing three times with PBS, 1 mL of PBS was added to each well, followed by the addition of 2 drops/well of NucBlue™ Live ReadyProbes™ Reagent (Invitrogen). The plate was covered with aluminum foil to block light, incubated at room temperature for 20 min, and observed under a fluorescence microscope.

### Statistical analysis

JMP^®^ statistical analysis software (JMP Statistical Discovery LLC, Cary, NC, USA) was used to perform Student’s *t*-test and Dunnett’s test to determine the significance between the hydrolyzed corn starch-treated and control groups. Data are presented as the mean ± SD and “n” depicts the number of biological replicates in each experiment. Values of *p* < 0.05 were considered significant.

## Results

### Effects of hydrolyzed corn starch treatment on the gene-expression profiles

**Fig 2:**
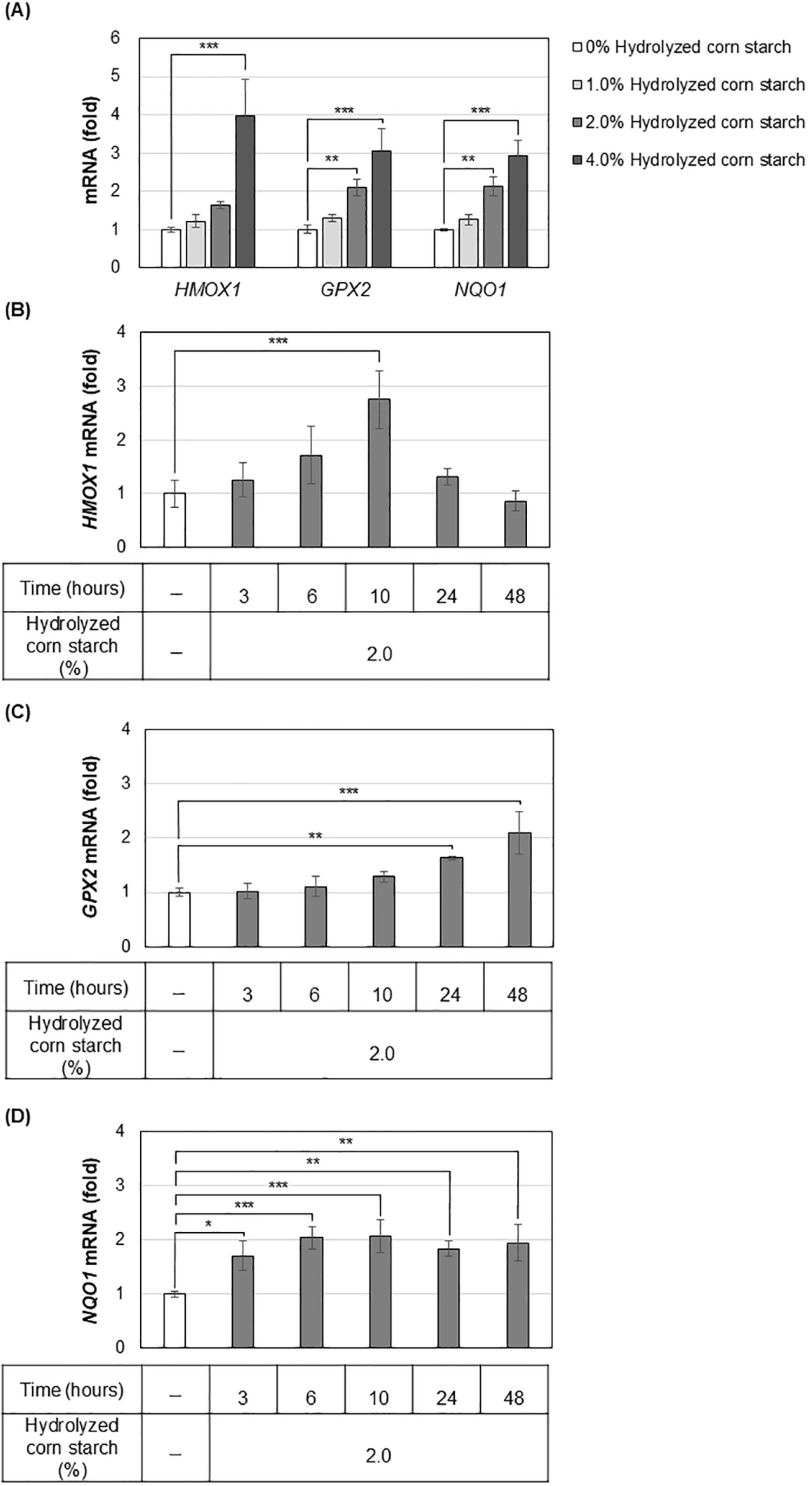
Effects of hydrolyzed corn starch on the mRNA expression levels of the oxidative stress-related genes in keratinocytes. (A) Cells were treated with 1.0%, 2.0%, and 4.0% hydrolyzed corn starch for 24 h. (B-D) Cells were treated with 2.0% hydrolyzed corn starch for 3, 6, 10, 24, and 48 h. The mRNA levels of *HMOX1* (B), *GPX2* (C), and *NQO1* (D) were determined by RT-qPCR and normalized to that of *GAPDH*. Dunnett’s test, *p < 0.05, **p < 0.01, ***p < 0.001 (n = 3).

The statistical comparison of control cells and those treated with 2.0% hydrolyzed corn starch for 24 h according to the Rank Products method with multiple testing corrections using the Benjamini and Hochberg FDR (*p* < 0.15) resulted in the selection of 73 probes (S1 Table). Subsequently, we used the online Database for Annotation, Visualization, and Integrated Discovery software module to identify the overrepresented Keywords and Gene Ontology (GO) terms in the selected genes. A functional analysis of the selected 73 probes demonstrated that the effects of hydrolyzed corn starch treatment indicated significant involvement in the oxidation reduction (KW-0560, GO:0016491). In addition, 14 genes related to the oxidation reduction were found (Table 1). Among these genes, *NQO1*, Aldo-keto reductase family 1 member C1 (*AKR1C1*), Aldo-keto reductase family 1 member C2 (*AKR1C2*), Aldo-keto reductase family 1 member C3 (*AKR1C3*), Cytochrome P450 family 4 subfamily F member 11 (*CYP4F11*), *GPX2*, and *HMOX1* were upregulated more than 2-fold by treatment with hydrolyzed corn starch. We focused on *HMOX1*, *GPX2*, and *NQO1*, which are commonly regulated by NRF2. To confirm the reproducibility of the microarray data on upregulation of these genes by hydrolyzed corn starch treatment, the mRNA expressions of *HMOX1*, *GPX2*, and *NQO1* in keratinocytes were measured by reverse transcription quantitative polymerase chain reaction (RT-qPCR) (Fig 2A). The findings indicated a concentration-dependent increase in mRNA expressions when treated with 1.0%, 2.0%, and 4.0% hydrolyzed corn starch for 24 h.

**Table 1:** Oxidation reduction-related genes.

UNIGENE	GENE NAME
Hs.406515	NAD(P)H quinone dehydrogenase 1 (*NQO1*)
Hs.460260	Aldo-keto reductase family 1 member C1 (*AKR1C1*)
Hs.460260, Hs.567256	Aldo-keto reductase family 1 member C2 (*AKR1C2*)
Hs.78183	Aldo-keto reductase family 1 member C3 (*AKR1C3*)
Hs.72912	Cytochrome P450 family 1 subfamily A member 1 (*CYP1A1*)
Hs.187393	Cytochrome P450 family 4 subfamily F member 11 (*CYP4F11*)
Hs.179608	Dehydrogenase/reductase 9 (*DHRS9*)
Hs.2704	Glutathione peroxidase 2 (*GPX2*)
Hs.517581	Heme oxygenase 1 (*HMOX1*)
Hs.596461	Isocitrate dehydrogenase (NADP(+)) 2 (*IDH2*)
Hs.464071	Phosphogluconate dehydrogenase (*PGD*)
Hs.495728	Pirin (*PIR*)
Hs.196384	Prostaglandin-endoperoxide synthase 2 (*PTGS2*)
Hs.654922	Thioredoxin reductase 1 (*TXNRD1*)

Furthermore, we examined the effect of hydrolyzed corn starch at different exposure times (3, 6, 10, 24, and 48 h) on the mRNA expression of these enzymes in keratinocytes (Fig 2B–2D). Hydrolyzed corn starch increased the mRNA expression of *HMOX1*, *GPX2*, and *NQO1* at different times in a gene dependent manner.

To further characterize the antioxidant response to hydrolyzed corn starch, additional antioxidant-related gene expression analyses were performed. The mRNA expression levels of *PRDX1*, *SOD1*, *CAT*, *GSR*, *GCLC*, and *TXN* were determined by RT-qPCR after 24 h treatment with 1.0%, 2.0%, and 4.0% hydrolyzed corn starch (S1 Fig). As shown in S1 Fig, the mRNA expression levels of *GCLC* and *TXN* were upregulated in a concentration-dependent manner, and that of *PRDX1* was significantly increased at 4.0% hydrolyzed corn starch compared with control (*p* < 0.05). In contrast, *SOD1*, *CAT*, and *GSR* showed no significant changes under these conditions.

### Effects of hydrolyzed corn starch on the ROS level in keratinocytes exposed to hydrogen peroxide (H_2_O_2_)

**Fig 3:**
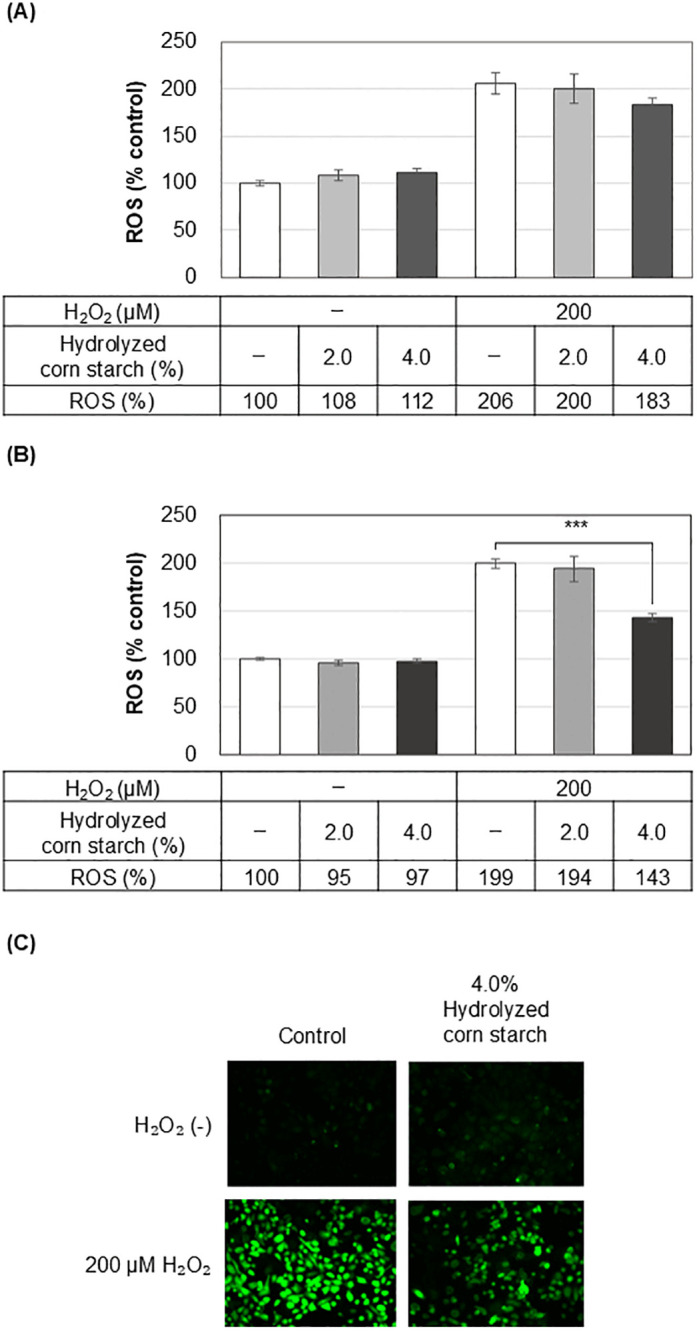
Effects of hydrolyzed corn starch on the reactive oxygen species (ROS) production in keratinocytes exposed to H_2_O_2_. (A, B) Cells were labeled with DCFH-DA, treated with 2.0% and 4.0% hydrolyzed corn starch for 24 h (A) and 48 h (B), and exposed to 200 μM H_2_O_2_ for 1 h. The fluorescence intensity of the cells was measured to determine the ROS levels. (C) Typical images of cells fluorescing due to the oxidation of DCFH-DA by ROS. Dunnett’s test, ***p < 0.001 (n = 3).

To evaluate the antioxidant response to treatment with 2.0% and 4.0% hydrolyzed corn starch for 24 and 48 h, intracellular ROS levels were measured using the fluorescent probe DCFH-DA (Fig 3A and 3B). As shown in Fig 3A and 3B, compared with the H_2_O_2_-untreated group, the intracellular ROS production was increased in the H_2_O_2_-treated group. Pretreatment with the hydrolyzed corn starch inhibited the increase in ROS production induced by H_2_O_2_-treatment, and 4.0% hydrolyzed corn starch after 48 h exposure significantly inhibited ROS production relative to that of the control (*p* < 0.001) (Fig 3B). Typical fluorescence images of the cells are shown in Fig 3C. The fluorescence caused by the oxidation of DCFH-DA was increased by H_2_O_2_, and the change was reduced by hydrolyzed corn starch.

### Effects of hydrolyzed corn starch on NRF2 activation

The activation of NRF2 occurs by translocating NRF2 into the nucleus from the cytosol. After NRF2 translocates into the nucleus, it can bind to the ARE and drive the expression of target genes, such as *HMOX1*, *GPX2*, and *NQO1*.

**Fig 4:**
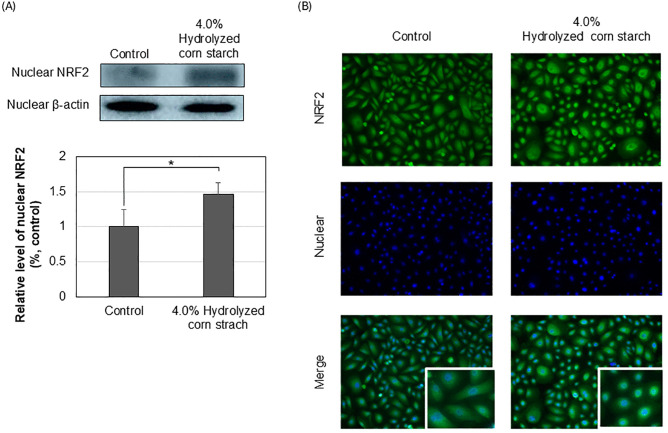
Effects of hydrolyzed corn starch on NRF2 activation in keratinocytes. (A) Cells were treated with 4.0% hydrolyzed corn starch for 2 h, and nuclear NRF2 protein levels were determined by western blotting and normalized to that of nuclear β-actin. Student’s *t*-test, *p < 0.05 (n = 3). (B) Nuclear translocation of NRF2 was determined by immunostaining.

To analyze the protein level of NRF2 in the nucleus, we performed western blotting on cells treated with 4.0% hydrolyzed corn starch for 2 h. β-actin served as a reference protein for normalization. As shown in Fig 4A, 4.0% hydrolyzed corn starch significantly increased NRF2 in the nucleus compared with that in the control (*p* < 0.05). Furthermore, Fig 4B shows that NRF2 is localized from the cytosol to the nucleus in the cells treated with 4.0% hydrolyzed corn starch for 2 h.

## Discussion

The skin generates ROS in response to external stimuli, such as ultraviolet radiation and air pollution. The transcription factor NRF2 plays a central role in the cellular defense mechanism against oxidative stress. Upon translocation from the cytoplasm to the nucleus, NRF2 binds to the ARE and upregulates genes, including *HMOX1*, *GPX2*, and *NQO1*. The *HMOX1* gene encodes heme oxygenase-1, which is a key enzyme that catalyzes the rate-limiting first step of heme degradation, yielding carbon monoxide, ferrous, and biliverdin. Biliverdin is rapidly reduced to bilirubin, a known strong antioxidant [14]. *GPX2* reduces hydrogen peroxide and lipid hydroperoxides at the expense of glutathione, whereas *NQO1* reduces quinone to hydroquinone. These antioxidant enzymes act both directly and indirectly to help alleviate oxidative stress in the long term [15]. Conversely, when ROS levels become excessively high, oxidative damage to DNA and proteins can occur along with increased synthesis and activation of matrix metalloproteinases, enzymes that degrade extracellular matrix components, and higher ROS levels are thought to promote collagen and elastin degradation, leading to skin aging, such as wrinkles and sagging [16,17]. Furthermore, ROS stimulate keratinocytes and release various melanocyte activating factors, such as endothelin-1 and protease-activated receptor-2, which is known to promote melanin synthesis and melanosome transport [18,19], and is thought to cause skin problems, such as age spots and pigmentation.

In this study, we demonstrated that hydrolyzed corn starch activates the NRF2 pathway in keratinocytes, leading to increase the expression of antioxidant-responsive genes and a subsequent reduction in ROS levels. Specifically, NRF2 activation occurred 2 h after the application of hydrolyzed corn starch, subsequently leading to ≥2-fold changes in *NQO1* at 6 h, *HMOX1* at 10 h, and *GPX2* at 48 h and ultimately leading to the inhibition of H_2_O_2_-dependent ROS generation at 48 h. These findings indicate that hydrolyzed corn starch enhances the cellular defense capacity against oxidative stress via the NRF2 pathway in keratinocytes.

In addition, previous studies have suggested that the expressions of *GCLC*, *TXN*, *PRDX1*, *SOD1*, *CAT*, and *GSR* can be regulated by NRF2 [2,20,21], although the extent of NRF2 involvement may vary depending on the cell type and experimental conditions. In our study, treatment with 1.0%, 2.0%, and 4.0% hydrolyzed corn starch for 24 h upregulated the mRNA expression levels of *GCLC* and *TXN* in a concentration-dependent manner and significantly increased that of *PRDX1* at 4.0%, whereas *SOD1*, *CAT*, and *GSR* showed no significant changes under these conditions. Taken together with the observed induction of *HMOX1*, *GPX2*, and *NQO1,* these additional changes in antioxidant‑related genes further support the possibility that the overall gene‑expression profile induced by hydrolyzed corn starch is, at least in part, a consequence of NRF2 activation, although contributions from other transcriptional regulators cannot be excluded.

Given that excessive ROS are known to contribute to collagen and elastin degradation and to hyperpigmentation through the activation of melanocyte-stimulation factors [16–19], NRF2 activation and ROS reduction by hydrolyzed corn starch raise the possibility that this ingredient may indirectly influence skin conditions such as wrinkles, sagging, and pigmentation. However, our current data are limited to antioxidant responses in keratinocytes, and we did not directly assess structural or functional endpoints (e.g., collagen content, skin elasticity, melanin production) in the skin. Therefore, any anti-wrinkle, anti-sagging, or depigmenting effects remain speculative and should be validated in future *in vitro* and clinical studies. In addition, our experiments were conducted exclusively in keratinocytes and did not address the role of dermal fibroblasts, which are crucial for extracellular matrix production and skin elasticity. Future investigations using dermal fibroblasts, melanocyte-keratinocyte co-culture systems, and *in vivo* or clinical evaluations will be required to clarify whether the NRF2 activation and ROS reduction observed here translate into measurable improvements in these skin conditions.

People with sensitive skin often have low-barrier skin function, making them susceptible to external stimuli and increased oxidative stress. The multifunctional profile of hydrolyzed corn starch, including moisturizing, itch-relief, and antioxidant effects via NRF2 activation, supports its potential application as a cosmetic ingredient for sensitive skin.

Moreover, recent studies have demonstrated that activation of the NRF2 pathway can suppress inflammation by inhibiting the expression of interleukin 6 and interleukin 1β, cytokines that exacerbate inflammation [22]. On the basis of these reports, the NRF2 activation observed in our study raises the possibility that hydrolyzed corn starch may have anti-inflammatory effects on the skin. However, we did not measure inflammatory markers, cytokine expression, or other indices of inflammation in our experiments. Thus, any anti-inflammatory effect of hydrolyzed corn starch should be regarded as a hypothesis derived from the known function of NRF2 rather than as an observed effect.

Our results also suggest that maltotetraose (MTO), a major component of hydrolyzed corn starch, may play a key role in NRF2 activation and subsequent ROS reduction in keratinocytes, although the detailed mechanism remains unclear. Whether ROS reduction is solely mediated by the NRF2 pathway and the specific mechanism by which MTO activates NRF2 are not yet established. In addition, we did not evaluate the overall status of enzymatic and non‑enzymatic antioxidant capacity in this study, and thus the global impact of MTO on the cutaneous antioxidant defense system remains unclear. A comprehensive understanding will require the identification of the individual compounds in the hydrolyzed corn starch, subsequent validation using each individual compound, and elucidation of the associated molecular interactions and signaling pathways with specific inhibitors.

## Conclusion

The main focus of this study was to evaluate the potential novel beneficial effects of hydrolyzed corn starch containing maltotetraose on the skin, particularly defense against oxidative stress. The results suggest that hydrolyzed corn starch enhances cellular antioxidant capacity through induction of antioxidant-responsive genes, thereby contributing to the reduction of ROS levels. Furthermore, the results also suggest that hydrolyzed corn starch enhances cellular defense against oxidative stress by NRF2 activation.

## Supporting information

S1 Table 73 differentially expressed probes. Cells were treated with 2.0% hydrolyzed corn starch for 24 h. Differentially expressed probes were identified by microarray analysis.
(DOCX)

S1 Fig Effects of hydrolyzed corn starch on the mRNA expression levels of antioxidant related genes. Cells were treated with 1.0%, 2.0%, and 4.0% hydrolyzed corn starch for 24 h.
(TIF)

S1 Raw images Original, uncropped Western blot images correspond to Fig 4A. (PDF)
