# Supplementary material for: Hydrolyzed corn starch with maltotetraose for skin defense through NRF2 pathway activation in human keratinocytes
Source: PLoS One. 2026 Jun 26;21(6):e0351422. doi: 10.1371/journal.pone.0351422 (PMC13309040; doi:10.1371/journal.pone.0351422)
Supplement: S1 Table — Cells were treated with 2.0% hydrolyzed corn starch for 24 h. Differentially expressed probes were identified by microarray analysis. (DOCX) [file pone.0351422.s001.docx]

| **UNIGENE** | **GENE NAME** | **FOLD CHANGE** | **P-VALUE** |
| --- | --- | --- | --- |
| Hs.460260 | *AKR1C1* | 3.45 | 2.9E-07 |
| Hs.517581 | *HMOX1* | 4.03 | 1.8E-06 |
| Hs.567256 | *AKR1C2* | 2.81 | 1.1E-05 |
| Hs.459709 | *PRSS22* | 0.35 | 7.8E-05 |
| Hs.926 | *MX2* | 0.38 | 1.7E-04 |
| Hs.2704 | *GPX2* | 2.43 | 2.4E-04 |
| Hs.368243 | *ABCC2* | 2.55 | 2.7E-04 |
| Hs.412117 | *ANXA6* | 0.42 | 6.5E-04 |
| Hs.78183 | *AKR1C3* | 2.32 | 5.8E-04 |
| Hs.375129 | *MMP3* | 2.31 | 1.1E-03 |
| Hs.187393 | *CYP4F11* | 2.22 | 1.1E-03 |
| Hs.211282 | *CRELD2* | 0.45 | 3.1E-03 |
| Hs.406515 | *NQO1* | 2.18 | 1.4E-03 |
| Hs.436446 | *MANF* | 0.46 | 4.9E-03 |
| Hs.390594 | *SLC7A11* | 2.10 | 3.0E-03 |
| Hs.83169 | *MMP1* | 2.09 | 2.8E-03 |
| Hs.677557 | *TMPRSS11F* | 2.14 | 3.3E-03 |
| Hs.183428 | *SSPN* | 2.08 | 5.3E-03 |
| Hs.406714 | *KRTAP2-3* | 0.48 | 8.9E-03 |
| Hs.20315 | *IFIT1* | 0.49 | 1.4E-02 |
| Hs.270364 | *LAMA1* | 0.49 | 1.5E-02 |
| Hs.744072 | *IFIT3* | 0.50 | 1.8E-02 |
| Hs.441664 | *TSPAN7* | 1.90 | 2.0E-02 |
| Hs.143250 | *TNC* | 0.51 | 2.3E-02 |
| Hs.650131 | *KRTAP2-2* | 0.51 | 2.1E-02 |
| Hs.93659 | *PDIA4* | 0.51 | 2.1E-02 |
| Hs.679230 | *OCLM* | 0.51 | 2.0E-02 |
| Hs.17518 | *RSAD2* | 0.51 | 2.0E-02 |
| Hs.72912 | *CYP1A1* | 1.88 | 2.3E-02 |
| Hs.528921 | *KRTAP2-1* | 0.52 | 2.4E-02 |
| Hs.352213 | *ZPLD1* | 1.89 | 2.6E-02 |
| Hs.182432 | *HIST1H2BM* | 0.53 | 3.8E-02 |
| Hs.403933 | *FBXO32* | 0.53 | 3.6E-02 |
| Hs.196384 | *PTGS2* | 1.84 | 3.9E-02 |
| Hs.512676 | *RPS25* | 1.83 | 3.7E-02 |
| Hs.438102 | *IGFBP2* | 0.53 | 3.7E-02 |
| Hs.518203 | *PARP14* | 0.53 | 3.7E-02 |
| Hs.495728 | *PIR* | 1.82 | 4.4E-02 |
| Hs.524692 | *NUAK1* | 0.54 | 4.7E-02 |
| Hs.517033 | *TGM2* | 0.54 | 5.9E-02 |
| Hs.464071 | *PGD* | 1.76 | 7.1E-02 |
| Hs.435371 | *TCP11* | 0.55 | 7.0E-02 |
| Hs.368348 | *PUS10* | 1.78 | 7.8E-02 |
| Hs.558218 | *CASP8AP2* | 1.75 | 8.3E-02 |
| Hs.425769 | *OMA1* | 1.75 | 8.1E-02 |
| Hs.260041 | *CASD1* | 1.75 | 8.0E-02 |
| Hs.493771 | *FAM219A* | 1.74 | 8.1E-02 |
| Hs.655123 | *KLC4* | 1.73 | 8.1E-02 |
| Hs.720813 | *ZNF223* | 1.73 | 8.8E-02 |
| Hs.248202 | *GLP2R* | 0.56 | 1.0E-01 |
| Hs.179608 | *DHRS9* | 0.56 | 1.1E-01 |
| Hs.134434 | *OVOL1* | 1.72 | 1.0E-01 |
| Hs.162200 | *PER3* | 1.71 | 9.9E-02 |
| Hs.503721 | *DYNC2H1* | 0.54 | 1.1E-01 |
| Hs.183656 | *VNN3* | 0.57 | 1.2E-01 |
| Hs.277704 | *HYOU1* | 0.57 | 1.1E-01 |
| Hs.411501 | *KRT7* | 0.57 | 1.1E-01 |
| Hs.596461 | *IDH2* | 0.57 | 1.2E-01 |
| Hs.187667 | *CTNS* | 1.69 | 1.2E-01 |
| Hs.658866 | *SYT14* | 1.69 | 1.1E-01 |
| Hs.438867 | *SLC48A1* | 1.69 | 1.1E-01 |
| Hs.654922 | *TXNRD1* | 1.70 | 1.1E-01 |
| Hs.545789 | *BAGE* | 1.65 | 1.1E-01 |
| Hs.419171 | *KIAA1671* | 0.58 | 1.3E-01 |
| Hs.24422 | *RFXAP* | 1.68 | 1.2E-01 |
| Hs.655087 | *MCTP1* | 1.66 | 1.2E-01 |
| Hs.435012 | *GALM* | 1.69 | 1.2E-01 |
| Hs.546467 | *EPSTI1* | 0.58 | 1.4E-01 |
| Hs.79361 | *KLK6* | 0.58 | 1.4E-01 |
| Hs.489118 | *SAMD9L* | 0.58 | 1.4E-01 |
| Hs.733762 | *ADGRF1* | 1.67 | 1.2E-01 |
| Hs.149195 | *PADI3* | 0.58 | 1.5E-01 |
| Hs.438385 | *C15orf61* | 1.68 | 1.3E-01 |
