# Supplementary material for: Hydrolyzed corn starch with maltotetraose for skin defense through NRF2 pathway activation in human keratinocytes
Source: PLoS One. 2026 Jun 26;21(6):e0351422. doi: 10.1371/journal.pone.0351422 (PMC13309040; doi:10.1371/journal.pone.0351422)

Original blot for Fig 4 (nuclear NRF2)
Lane 1: marker, Lane 2, 5, 6, 7: X, Lane 3: 0% Hydrolyzed corn starch (Control),
Lane 4: 4.0% (2h) Hydrolyzed corn starch
Imaging system: Amersham ImageQuant 800 (Cytiva), ECL substrate

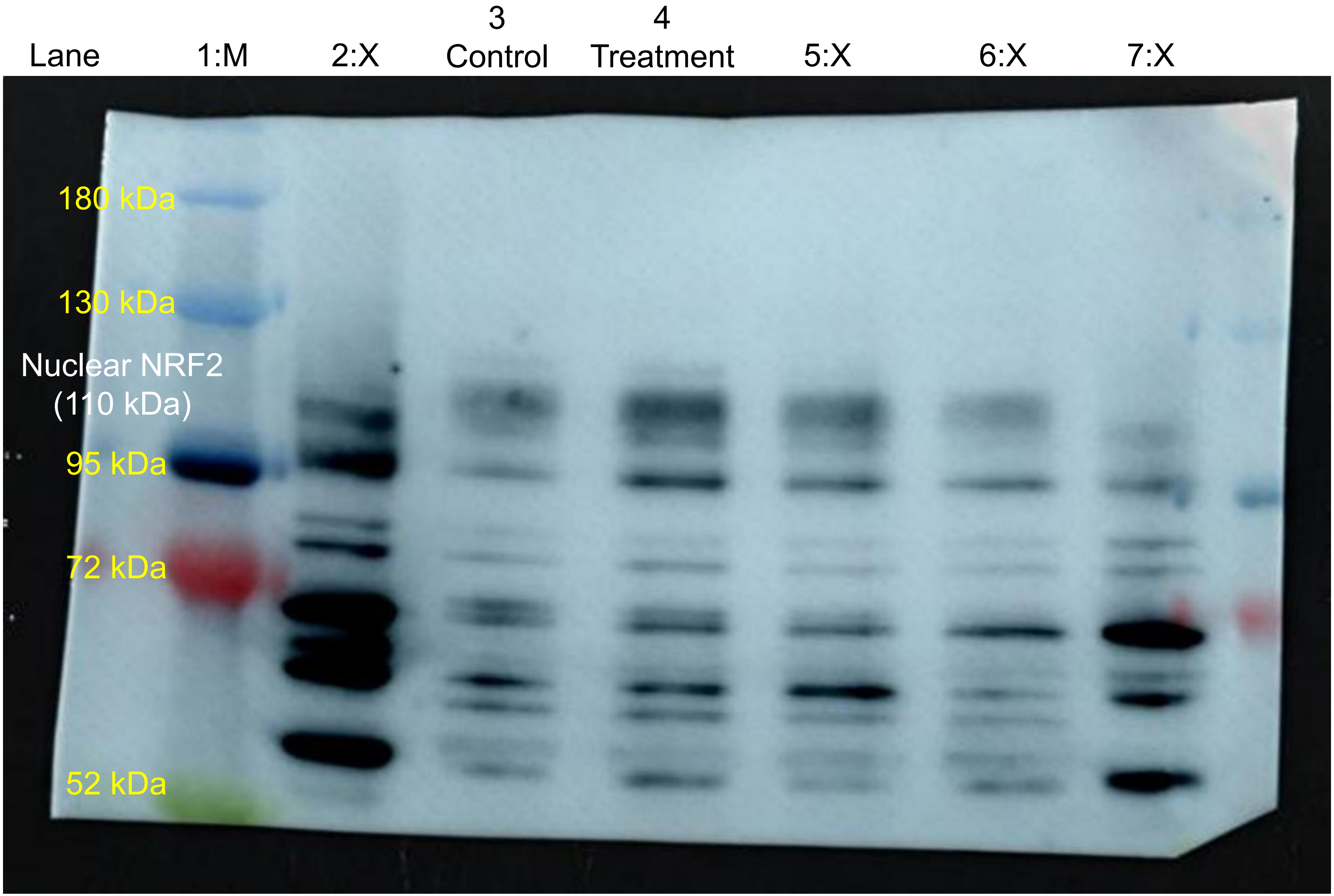

Original blot for Fig 4 (nuclear β-actin)
Lane 1: marker, Lane 2, 5, 6, 7: X, Lane 3: 0% Hydrolyzed corn starch (Control),
Lane 4: 4.0% (2h) Hydrolyzed corn starch
Imaging system: Amersham ImageQuant 800 (Cytiva), ECL substrate

Lane	1:M	2:X	3 Control	4 Treatment	5:X	6:X	7:X

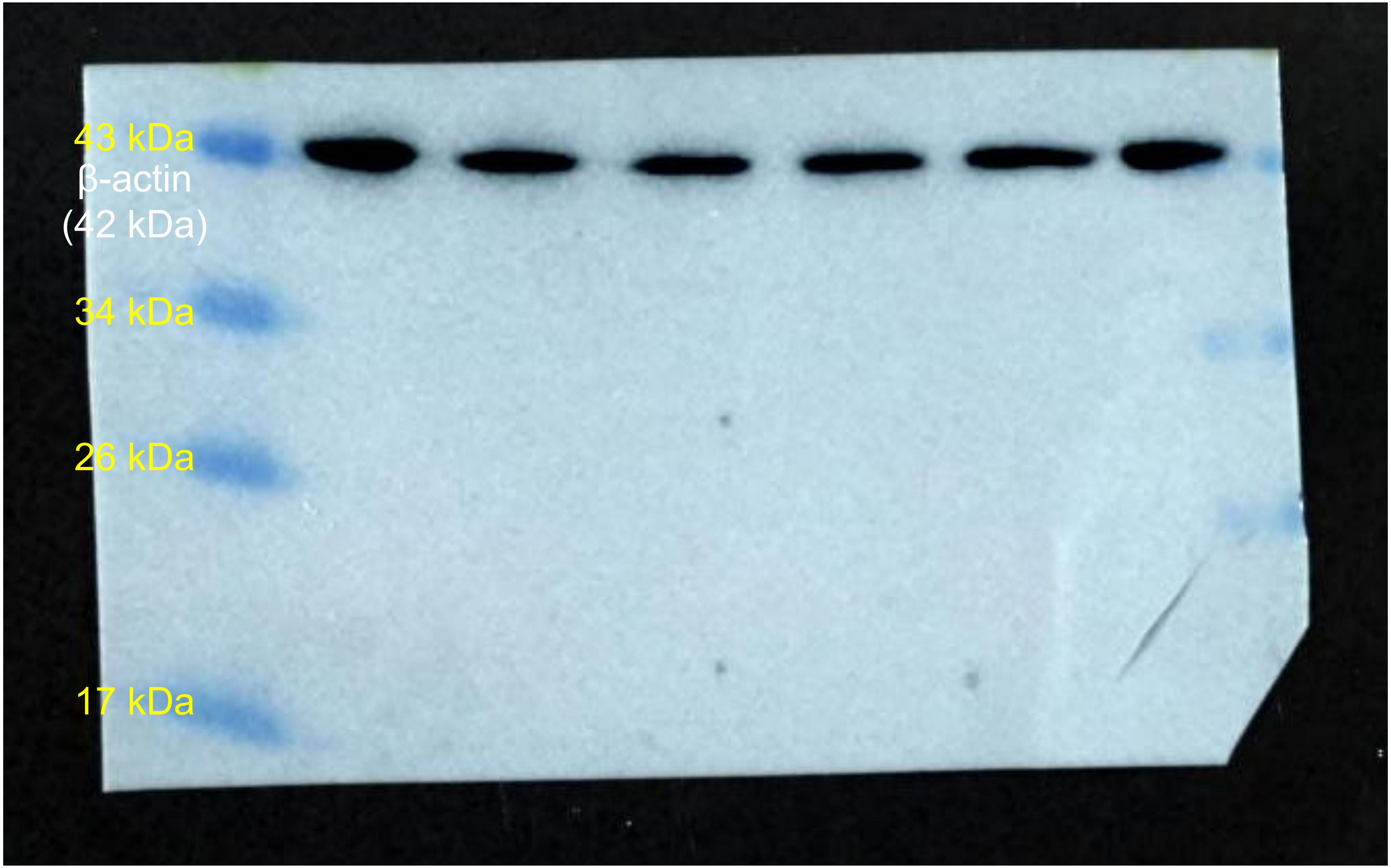

Supplement: S1 Raw images — (PDF) [file pone.0351422.s002.pdf]
