# Supplementary material for: Hydrolyzed corn starch with maltotetraose for skin defense through NRF2 pathway activation in human keratinocytes
Source: PLoS One. 2026 Jun 26;21(6):e0351422. doi: 10.1371/journal.pone.0351422 (PMC13309040; doi:10.1371/journal.pone.0351422)
Supplement: Attachment — Submitted filename: Response to Reviewers.pdf [file pone.0351422.s006.pdf]

[Point-by-point response] (Black: Editor's and Reviewer's comments, Red: our responses)

Journal Requirement (Editor)

1. Please ensure that your manuscript meets PLOS ONE's style requirements, including those for file naming. The PLOS ONE style templates can be found at https://journals.plos.org/plosone/s/file?id=wjVg/PLOSOne_formatting_sample_main_body.pdf and https://journals.plos.org/plosone/s/file?id=ba62/PLOSOne_formatting_sample_title_authors_affiliations.pdf.

We have carefully revised the manuscript to ensure that it follows the PLOS ONE style requirements, including those for file naming and formatting. We have consulted and applied the formatting guidelines provided in the PLOS ONE style templates.

2. Thank you for stating in your Funding Statement: This research was supported financially by the Lion Corporation. No other specific grant was received from funding agencies in the public, commercial, or not-for-profit sectors." Please provide an amended statement that declares *all* the funding or sources of support (whether external or internal to your organization) received during this study, as detailed online in our guide for authors at http://journals.plos.org/plosone/s/submit-now. Please also include the statement "There was no additional external funding received for this study." in your updated Funding Statement.

Please include your amended Funding Statement within your cover letter. We will change the online submission form on your behalf.

We appreciate the editor's guidance regarding the Funding Statement. We have revised the Funding Statement to read as follows, and will include this updated version in the cover letter:

"This research was supported financially by the Lion Corporation. No other specific grant was received from funding agencies in the public, commercial, or not-for-profit sectors. There was no additional external funding received for this study."

We understand that the online submission form will be updated by the editorial office based on the text provided in the cover letter.

3. Thank you for stating the following financial disclosure:

“This research was supported financially by the Lion Corporation. No other specific grant was received from funding agencies in the public, commercial, or not-for-profit sectors.” Please state what role the funders took in the study. If the funders had no role, please state: "The funders had no role in study design, data collection and analysis, decision to publish, or preparation of the manuscript." If this statement is not correct you must amend it as needed. Please include this amended Role of Funder statement in your cover letter; we will change the online submission form on your behalf.

We thank the editor for pointing out the need to clarify the role of the funder.

In accordance with the journal’s request, we have added the following statement regarding the role of the funder, which will be included in the cover letter:

"The funders had no role in study design, data collection and analysis, decision to publish, or preparation of the manuscript."

We understand that the editorial office will update the corresponding section in the online submission system based on this statement.

4. We note that you have indicated that there are restrictions to data sharing for this study. For studies involving human research participant data or other sensitive data, we encourage authors to share de-identified or anonymized data. However, when data cannot be publicly shared for ethical reasons, we allow authors to make their data sets available upon request. For information on unacceptable data access restrictions, please see http://journals.plos.org/plosone/s/data-availability#loc-unacceptable-data-access-restrictions. Before we proceed with your manuscript, please address the following prompts:

a) If there are ethical or legal restrictions on sharing a de-identified data set, please explain them in detail (e.g., data contain potentially identifying or sensitive patient information, data are owned by a third-party organization, etc.) and who has imposed them (e.g., a Research Ethics Committee or Institutional Review Board, etc.). Please also provide contact information for a data access committee, ethics committee, or other institutional body to which data requests may be sent.

b) If there are no restrictions, please upload the minimal anonymized data set necessary to replicate your study findings to a stable, public repository and provide us with the relevant URLs, DOIs, or accession numbers. Please see http://www.bmj.com/content/340/bmj.c181.long

for guidelines on how to de-identify and prepare clinical data for publication. For a list of recommended repositories, please see https://journals.plos.org/plosone/s/recommended-repositories. You also have the option of uploading the data as Supporting Information files, but we would recommend depositing data directly to a data repository if possible.

Please update your Data Availability statement in the submission form accordingly.

We have revised the Data Availability statement in the manuscript to more clearly describe where the data can be accessed. The revised statement reads as follows: However, the microarray data have not yet been assigned an accession number in GEO. The revised statement reads as follows:

"All microarray data files have been deposited in the NCBI Gene Expression Omnibus (GEO)

under accession number GSEXXXXXX (to be updated once the accession number is assigned). All other datasets used for the statistical analyses, including individual data points and sample sizes for each experiment, have been deposited in the Zenodo repository and are freely available under the accession DOI 10.5281/zenodo.19227766. All other relevant data are within the manuscript and its Supporting Information files." (line 372–380)

We hope that this revision sufficiently clarifies the sample sizes and the accessibility of all datasets used in our analyses and ensures compliance with the PLOS ONE Data Availability policy.

5. In the online submission form you indicate that your data is not available for proprietary reasons and have provided a contact point for accessing this data. Please note that your current contact point is a co-author on this manuscript. According to our Data Policy, the contact point must not be an author on the manuscript and must be an institutional contact, ideally not an individual. Please revise your data statement to a non-author institutional point of contact, such as a data access or ethics committee, and send this to us via return email. Please also include contact information for the third party organization, and please include the full citation of where the data can be found.

In response to the journal's data policy, we have revised our data sharing plan so that the underlying datasets are now publicly available and no longer subject to proprietary access restrictions.

As noted above, the microarray data have been submitted to the NCBI Gene Expression

Omnibus (GEO) and will be available under accession GSEXXXXXX once it is assigned. All other datasets used for statistical analyses have been deposited in Zenodo (DOI 10.5281/zenodo.19227766). Therefore, we no longer rely on a "data available upon request" model, and no author will serve as a direct data-access contact point.

Because the data are openly available through these public repositories, an additional institutional contact point for data access is no longer required.

6. Please be informed that funding information should not appear in the Acknowledgments section or other areas of your manuscript. We will only publish funding information present in the Funding Statement section of the online submission form. Please remove any funding-related text from the manuscript.

We acknowledge the journal's policy that funding information should only appear in the Funding Statement section of the online submission form and not in the Acknowledgments or other parts of the manuscript. Accordingly, we have removed all funding-related text from the Acknowledgments section and from any other location in the manuscript.

7. We note that you have included the phrase "data not shown" in your manuscript. Unfortunately, this does not meet our data sharing requirements. PLOS does not permit references to inaccessible data. We require that authors provide all relevant data within the paper, Supporting Information files, or in an acceptable, public repository. Please add a citation to support this phrase or upload the data that corresponds with these findings to a stable repository (such as Figshare or Dryad) and provide and URLs, DOIs, or accession numbers that may be used to access these data. Or, if the data are not a core part of the research being presented in your study, we ask that you remove the phrase that refers to these data.

We thank the editor and reviewers for drawing our attention to the use of the phrase "data not shown" and for emphasizing the importance of data transparency. In response to the concern that "data not shown" is not acceptable for key results and that the underlying data must be available, we have now provided the supporting microarray data for the 73 selected probes as a supplementary file (S1 Table). These changes ensure that all relevant data supporting our findings are accessible to readers and fully comply with PLOS ONE's data sharing requirements.

8. PLOS ONE now requires that authors provide the original uncropped and unadjusted images underlying all blot or gel results reported in a submission's figures or Supporting Information

files. This policy and the journal’s other requirements for blot/gel reporting and figure preparation are described in detail at https://journals.plos.org/plosone/s/figures#loc-blot-and-gel-reporting-requirements and https://journals.plos.org/plosone/s/figures#loc-preparing-figures-from-image-files. When you submit your revised manuscript, please ensure that your figures adhere fully to these guidelines and provide the original underlying images for all blot or gel data reported in your submission. See the following link for instructions on providing the original image data: https://journals.plos.org/plosone/s/figures#loc-original-images-for-blots-and-gels. In your cover letter, please note whether your blot/gel image data are in Supporting Information or posted at a public data repository, provide the repository URL if relevant, and provide specific details as to which raw blot/gel images, if any, are not available. Email us at plosone@plos.org if you have any questions.

We understand and appreciate the journal’s policy requiring the submission of original, uncropped, and unadjusted images for all blot and gel data. In accordance with this policy, we have prepared and submitted the raw Western blot images underlying Figure 4 as Supporting information (S1_raw_images.pdf). These files correspond directly to the processed images shown in Figure 4. We will clearly indicate in the cover letter that the raw blot images for Figure 4 have been provided, and we confirm that there are no additional blot or gel images in the manuscript for which raw data are unavailable.

9. If the reviewer comments include a recommendation to cite specific previously published works, please review and evaluate these publications to determine whether they are relevant and should be cited. There is no requirement to cite these works unless the editor has indicated otherwise.

We note that the reviewers did not explicitly recommend any specific previously published works that must be cited in this manuscript. Accordingly, no reviewer-suggested citations needed to be added. We have, however, reviewed and updated the references in the Introduction and Discussion where appropriate to ensure that the most relevant and recent literature is cited.

Reviewer #1

1. Repetition of the phrase "hydrolyzed corn starch containing maltotetraose" affects flow and should be reduced in the Abstract section.

We appreciate this helpful comment. In the revised Abstract, we now use the full expression "hydrolyzed corn starch containing maltotetraose" only when the ingredient is first introduced and again in the concluding sentence where we summarize its significance. In the rest of the Abstract, we refer to it more concisely as "this ingredient (line: 24 and 32)," "it," (line: 25) or "the ingredient" (line: 29-30) where the meaning is clear. We believe this has reduced redundancy and improved the readability of the Abstract.

2. No pigmentation-related markers, melanocyte models, or aging-associated endpoints are mentioned.

We appreciate insightful comments. We fully agree that our current study did not include pigmentation-related markers, melanocyte-based models, or aging-associated endpoints, and that any statements regarding pigmentation or aging must therefore be made with caution.

In response to this comment, we have revised the Abstract and Discussion to clearly state that the data presented in this study are limited to antioxidant responses and NRF2 activation in keratinocytes. Any potential implications for pigmentation or aging are now explicitly described as hypothetical and not directly evaluated in this work. In the Abstract we now state:

"This NRF2-mediated antioxidative activity positions hydrolyzed corn starch containing maltotetraose as a promising ingredient for cosmetic products, with potential implications for improving skin health, reducing pigmentation, and combating aging. However, these effects were not directly evaluated in this study. Future studies are warranted to evaluate its efficacy *in vivo*." (line: 33-38)

In the Discussion, we also note that:

"However, our current data are limited to antioxidant responses in keratinocytes, and we did not directly assess structural or functional endpoints (e.g., collagen content, skin elasticity, melanin production) in the skin. Therefore, any anti-wrinkle, anti-sagging, or depigmenting effects remain speculative and should be validated in future *in vitro* and clinical studies." (line: 324-328)

We hope that these revisions clarify the scope and limitations of our study.

3. In line 32, the phrase "novel functionality" is vague and should be replaced with more precise scientific language.

We appreciate this helpful comment. We agree that the term "novel functionality" was vague. In the revised manuscript, we have replaced this phrase with the more specific and scientifically accurate term "NRF2-mediated antioxidative activity," (line: 34) which directly reflects the mechanism demonstrated in our study. The sentence now reads:

"This NRF2-mediated antioxidative activity positions hydrolyzed corn starch containing maltotetraose as a promising ingredient for cosmetic products, with potential implications for improving skin health, reducing pigmentation, and combating aging." (line: 33-36)

This revision clarifies that the functionality in question is the activation of the NRF2 pathway leading to an enhanced antioxidative response.

4. Typographical error identified: "maltotetoraose" should be corrected to "maltotetraose" in line 33.

We thank the reviewer for pointing out this typographical error. In the revised manuscript, we have corrected the misspelling "maltotetoraose" to "maltotetraose" on line 35. We have also checked the entire manuscript to ensure that the term "maltotetraose" is spelled consistently throughout.

5. In line 56, the statement that NRF2 is a "promising target in cosmetic applications" is plausible but under-argued. Clarify why moderate NRF2 activation is beneficial and safe in cosmetic formulations.

We appreciate this valuable comment. We agree that additional explanation was needed to clarify why NRF2 can be regarded as a beneficial and safe target in the context of cosmetic formulations.

In the revised Introduction, we have elaborated on the role of NRF2 in skin and the rationale for targeting it in cosmetics. (line: 59-67) Specifically, we now emphasize that:

•NRF2 regulates a broad range of antioxidant and phase II detoxification enzymes that detoxify and eliminate reactive oxygen species and other oxidative agents, thereby contributing to photoprotection, regulation of pigmentation, wound healing, and reinforcement of epidermal

barrier function [3,5-7]. Enhancement of NRF2 signaling is therefore expected to strengthen endogenous antioxidant and barrier defenses, thereby helping to prevent photoaging, pigmentation disorders, and barrier impairment induced by environmental stressors.

•Transient NRF2 activation is unlikely to be harmful, as NRF2 upregulates Keap1 expression via a negative feedback mechanism that contributes to the maintenance of physiological redox balance in cells.

We hope that this clarification addresses the concern regarding why moderate NRF2 activation can be considered both beneficial and safe in cosmetic formulations.

6. "Raw and functional cosmetic ingredient" is vague. Consider replacing with "naturally derived functional cosmetic ingredient" in line 57.

Thank you for pointing this out. We agree that the previous wording was ambiguous. In the revised manuscript, we have replaced "raw and functional cosmetic ingredient" with a clearer description. The relevant sentence now reads: "We have identified hydrolyzed corn starch, derived from corn, as a safe and effective naturally derived functional cosmetic ingredient." (line: 71-72)

This wording better reflects both the natural origin and the functional role of the ingredient in cosmetic formulations.

7. The assertion that MTO has "superior skin-moisturizing properties" based on hydroxyl group content alone is chemically simplistic. Provide experimental or literature-based evidence supporting superior humectancy, or rephrase to a more cautious claim (e.g., "potentially enhanced moisturizing capacity") in line 60.

We fully agree that our initial wording was too strong given that it was based only on the number of hydroxyl groups. In accordance with your suggestion, we have revised this part of the text to adopt a more cautious wording. The sentence now reads:

"Given its higher concentration of hydroxyl groups compared to other humectants, such as glycerin and butylene glycol, MTO has potentially enhanced moisturizing capacity." (line: 74-76)

By using the phrase "potentially enhanced moisturizing capacity," we avoid overinterpreting the chemical structure alone and clearly indicate that superior moisturizing efficacy is a possibility

rather than an established fact.

Where relevant, we refer to our clinical study demonstrating moisturizing and barrier-improving effects of an MTO-containing moisturizer [13], which supports the practical usefulness of MTO in skin hydration without attributing this solely to the number of hydroxyl groups.

8. In line 184, the term “robes” appears to be a typographical error and should be corrected to “probes”. The phrase “data not shown” is not acceptable for a primary result in a manuscript. Transparency requires that the underlying data be accessible.

We thank the reviewer for carefully pointing out the typographical error and the issue regarding data transparency.

First, the spelling error “robes” has been corrected to “probes” in the revised manuscript. (line: 219)

Second, in response to the reviewer’s comment that “data not shown” is not acceptable for major results and that the underlying data should be accessible, we have now provided the supporting microarray data (S1 Table) for the selected 73 probes. (line: 219)

9. Reporting only the number of selected probes (n = 73) without presenting any supporting data (e.g., fold changes, adjusted p-values, or gene identities) is insufficient. At a minimum, a supplementary table should be provided.

We have added S1 Table, which lists, for each of the 73 probes, the gene name, Unigene, fold change, and p-value. We believe these changes improve the transparency and reproducibility of our analysis and adequately address the reviewer’s concern.

10. The discussion appropriately outlines the NRF2–ARE antioxidant pathway and its relevance to skin oxidative stress. However, the conclusion that hydrolyzed corn starch mitigates wrinkles, sagging, and pigmentation is speculative and not directly supported by the presented data.

We agree that our original wording could be interpreted as claiming direct evidence for anti-wrinkle, anti-sagging, and depigmenting effects, which is not supported by our current dataset. Accordingly, we have revised the Discussion to clearly distinguish between our experimental findings and potential implications.

The following paragraphs were added:

“Given that excessive ROS are known to contribute to collagen and elastin degradation and to hyperpigmentation through the activation of melanocyte-stimulation factors [16-19], NRF2

activation and ROS reduction by hydrolyzed corn starch raise the possibility that this ingredient may indirectly influence skin conditions such as wrinkles, sagging, and pigmentation. However, our current data are limited to antioxidant responses in keratinocytes, and we did not directly assess structural or functional endpoints (e.g., collagen content, skin elasticity, melanin production) in the skin. Therefore, any anti-wrinkle, anti-sagging, or depigmenting effects remain speculative and should be validated in future *in vitro* and clinical studies. In addition, our experiments were conducted exclusively in keratinocytes and did not address the role of dermal fibroblasts, which are crucial for extracellular matrix production and skin elasticity. Future investigations using dermal fibroblasts, melanocyte-keratinocyte co-culture systems, and *in vivo* or clinical evaluations will be required to clarify whether the NRF2 activation and ROS reduction observed here translate into measurable improvements in these skin conditions." (line: 321-332)

11. Justify this statement: the anti-inflammatory effect is inferred solely from literature citing NRF2-mediated suppression of IL-6 and IL-1β. Since no inflammatory markers were measured in this study, this conclusion should be framed as a potential implication, not as an observed effect in lines 265-268.

We fully concur with this comment. In the revised Discussion, we explicitly state that any anti-inflammatory effect is hypothetical and derived from known NRF2 functions, not from our own measurements. The relevant part now reads:

"Moreover, recent studies have demonstrated that activation of the NRF2 pathway can suppress inflammation by inhibiting the expression of interleukin 6 and interleukin 1β, cytokines that exacerbate inflammation [16]. On the basis of these reports, the NRF2 activation observed in our study raises the possibility that hydrolyzed corn starch may have anti-inflammatory effects on the skin. However, we did not measure inflammatory markers, cytokine expression, or other indices of inflammation in our experiments. Thus, any anti-inflammatory effect of hydrolyzed corn starch should be regarded as a hypothesis derived from the known function of NRF2 rather than as an observed effect." (line: 340-346)

Reviewer #2

1. Please provide latest references for the second paragraph of the Introduction section.

We appreciate your suggestion to include more recent references supporting the role of NRF2 in skin biology and pathology. In the revised version of the Introduction (second paragraph, line: 50-67), we have added the following key references:

[5] Schäfer M, Werner S. Nrf2-A regulator of keratinocyte redox signaling. Free Radical Biology and Medicine. 2015;88: 243-252

[6] Salman S, Paulet V, Hardonniére K, Kerdine-Römer S. The role of NRF2 transcription factor in inflammatory skin diseases. Biofactors. 2025;51(2)

[7] Kim HJ, Hong JH. Emerging Therapeutic Strategies for Nrf2-Associated Skin Disorders: From Photoaging to Autoimmunity. Antioxidants. 2026;15(1): 69

These references have been incorporated into the second paragraph of the Introduction to better substantiate the description of NRF2 as a central regulator of cutaneous antioxidant and cytoprotective responses, and to align the manuscript with the latest advances in the field.

2. If possible please provide a schematic to explain the pathways during presence and absence of oxidative stress.

Thank you for this helpful suggestion. In the revised manuscript, we have added a schematic illustration of the NRF2–Keap1 pathway under basal and oxidative stress conditions.

The new figure is included as Figure 1 and is described as follows:

"Fig 1. NRF2-Keap1 pathway under basal and oxidative stress conditions.

Under basal conditions, NRF2 is retained in the cytoplasm through its interaction with Keap1, which promotes NRF2 ubiquitination and proteasomal degradation. In the presence of oxidative stress, structural changes in Keap1 lead to the release and stabilization of NRF2. The stabilized NRF2 translocates into the nucleus, where it binds to ARE in the promoters of target genes. This induces the expression of phase II detoxification enzymes, antioxidant enzymes, and other cytoprotective molecules." (line: 90-96)

We believe this schematic will facilitate readers' understanding of the molecular mechanism by which NRF2 contributes to the skin's defense against oxidative stress and will clarify the rationale for targeting NRF2 in our study

3. Is there previously available literature that supports hydrolyzed corn starch or MTO f as a potent cosmetic ingredient?

Thank you for this important comment. We agree that the basis for describing hydrolyzed corn starch as a promising cosmetic ingredient should be clarified.

In the revised manuscript, we have added one sentence in the Introduction to explicitly indicate the official cosmetic classification of hydrolyzed corn starch:

“Hydrolyzed corn starch is officially categorized as a skin conditioning agent, specifically a humectant, in cosmetic ingredient nomenclature [8].” (line: 70-71)

We hope that this clarification in the Introduction, sufficiently addresses the reviewer’s concern regarding the existing evidence supporting hydrolyzed corn starch as a cosmetic ingredient.

4. The data of 73 probes can be provided as supplementary information?

We agree with the reviewer that the data for the 73 selected probes should be made available as supplementary information. In the revised manuscript, we have therefore added Supplementary S1 Table, which provides, for each of the 73 probes, the gene name, Unigene, fold change, and p-value. The corresponding text in the Results section has also been updated to explicitly refer to S1 Table instead of “data not shown.” (line: 219)

We hope that this supplementary table sufficiently addresses the reviewer’s request for access to the underlying data.

5. Need more clarity on the sample size and availability of the statistical analysis data.

We appreciate the reviewer’s comment regarding the need for clearer information on sample size and statistical analyses. In the revised manuscript, we have clarified the sample size (n) and statistical methods as follows.

•For the DNA microarray analysis, we now specify that “the CEL files obtained from three independent samples per condition (n = 3)”. (line: 126-127)

•For the gene expression and western blotting experiments, we now state that “ Three replicate wells were evaluated for each group (n = 3)” in the Materials and Methods sections (line: 159, 170, 193).

•The figure legends (e.g., Fig 2 (line: 247-254)) have been revised to indicate the statistical test used (Dunnett's test) and the significance thresholds, together with the sample size (n = 3).

•We have revised the Data Availability statement in the manuscript as follows:

"All microarray data files have been deposited in the NCBI Gene Expression Omnibus (GEO) under accession number **GSEXXXXXXX (to be updated once the accession number is assigned)**. All other datasets used for the statistical analyses, including individual data points and sample sizes for each experiment, have been deposited in the Zenodo repository and are freely available under the accession DOI 10.5281/zenodo.19227766. All other relevant data are within the manuscript and its Supporting Information files." (line: 372-380)

We hope that this revision sufficiently clarifies the sample sizes and the accessibility of all datasets used in our analyses.

Reviewer #3

1. The bar graphs are not properly labeled on the x-axis, and statistical significance is not indicated.

We appreciate the reviewer's valuable comment regarding the x-axis labels and the indication of statistical significance.

In response, we have revised the figures as follows:

Fig 2: We performed a Dunnett's multiple comparison test and have now indicated statistically significant differences on the graph. In addition, we adjusted the positions of the x-axis labels to improve clarity and readability.

Fig 3: We conducted a Student's t-test and have added the corresponding statistical significance to the graph. Similarly, we optimized the placement of the x-axis labels in this figure.

We believe these revisions address the reviewer's concerns and improve the quality and interpretability of the figures.

2. The authors claim that maltotetraose is a promising ingredient for cosmetic applications with potential roles in improving skin health, reducing pigmentation, and combating aging. However, no experimental data are provided to support claims related to pigmentation reduction or anti-aging effects. Moreover, the experiments were conducted exclusively in keratinocytes. The role of dermal fibroblasts, which are critical for maintaining skin elasticity and extracellular matrix integrity, has not been addressed.

We appreciate this important comment and have revised the Discussion to avoid overstatement and to clarify the limitations of our study design. We now explicitly acknowledge that our data are restricted to keratinocyte responses and do not include fibroblast or pigmentation endpoints. The following statements were added:

"Given that excessive ROS are known to contribute to collagen and elastin degradation and to hyperpigmentation through the activation of melanocyte-stimulation factors [16-19], NRF2 activation and ROS reduction by hydrolyzed corn starch raise the possibility that this ingredient may indirectly influence skin conditions such as wrinkles, sagging, and pigmentation. However, our current data are limited to antioxidant responses in keratinocytes, and we did not directly assess structural or functional endpoints (e.g., collagen content, skin elasticity, melanin production) in the skin. Therefore, any anti-wrinkle, anti-sagging, or depigmenting effects remain speculative and should be validated in future in vitro and clinical studies. In addition,

our experiments were conducted exclusively in keratinocytes and did not address the role of dermal fibroblasts, which are crucial for extracellular matrix production and skin elasticity. Future investigations using dermal fibroblasts, melanocyte-keratinocyte co-culture systems, and in vivo or clinical evaluations will be required to clarify whether the NRF2 activation and ROS reduction observed here translate into measurable improvements in these skin conditions." (line: 321-332)

Furthermore, we have revised the description of maltotetraose (MTO) so that it is no longer presented as a definitively "promising" cosmetic ingredient, but rather positioned as a mechanistic hypothesis based on the current data.

"Our results also suggest that maltotetraose (MTO), a major component of hydrolyzed corn starch, may play a key role in NRF2 activation and subsequent ROS reduction in keratinocytes, although the detailed mechanism remains unclear. Whether ROS reduction is solely mediated by the NRF2 pathway and the specific mechanism by which MTO activates NRF2 are not yet established. In addition, we did not evaluate the overall status of enzymatic and non-enzymatic antioxidant capacity in this study, and thus the global impact of MTO on the cutaneous antioxidant defense system remains unclear. A comprehensive understanding will require the identification of the individual compounds in the hydrolyzed corn starch, subsequent validation using each individual compound, and elucidation of the associated molecular interactions and signaling pathways with specific inhibitors." (line: 350-359)

3. The discussion section requires substantial improvement to better interpret the findings and place them in the context of existing literature.

We have reorganized the Discussion to follow a more logical progression:

(1) overview of NRF2–ARE signaling and ROS biology in the skin

(2) summary of our key findings (NRF2 activation, gene expression, and ROS reduction in keratinocytes)

(3) additional findings (expression of other antioxidant related genes)

(4) cautious discussion of potential implications for skin aging and pigmentation, explicitly framed as hypotheses

(5) clarification of limitations, including the lack of data on fibroblasts, extracellular matrix, pigmentation, and inflammatory markers

(6) future directions, including the need for compound-level analyses, mechanistic studies with inhibitors, and clinical validation.

4. The authors evaluated NRF2-associated antioxidant response genes, including HMOX1, GPX2, and NQO1. However, the effects of maltotetraose on other antioxidant systems, such as PRDX1, as well as overall enzymatic and non-enzymatic antioxidant status, were not investigated.

We appreciate the reviewer's suggestion to further investigate the broader antioxidant system beyond NRF2-related genes such as *HMOX1*, *GPX2*, and *NQO1*. In response, we performed additional analyses of antioxidant-related gene expression. Specifically, we quantified the mRNA expression levels of peroxiredoxin 1 (*PRDX1*), superoxide dismutase 1 (*SOD1*), catalase (*CAT*), glutathione reductase (*GSR*), glutamate-cysteine ligase catalytic subunit (*GCLC*), and thioredoxin (*TXN*) by RT-qPCR after treatment with 1.0%, 2.0%, and 4.0% hydrolyzed corn starch for 24 h. As shown in the revised Results section and Fig. S1, *GCLC* and *TXN* were upregulated in a concentration-dependent manner, and *PRDX1* was significantly increased at 4.0%, whereas *SOD1*, *CAT*, and *GSR* showed no significant changes.

These findings have been incorporated into the Results (line: 238-244) and discussed in the Discussion section (line: 311-320). In addition, we explicitly noted in the revised Discussion that "we did not evaluate the overall status of enzymatic and non-enzymatic antioxidant capacity in this study, and thus the global impact of maltotetraose on the cutaneous antioxidant defense system remains unclear." (line: 354-356)
