# Supplementary material for: Hydrolyzed corn starch with maltotetraose for skin defense through NRF2 pathway activation in human keratinocytes
Source: PLoS One. 2026 Jun 26;21(6):e0351422. doi: 10.1371/journal.pone.0351422 (PMC13309040; doi:10.1371/journal.pone.0351422)
Supplement: Attachment — Submitted filename: PONE-D-25-63339_R1.pdf [file pone.0351422.s005.pdf]

PLOS One

Hydrolyzed corn starch with maltotetraose for skin defense through NRF2 pathway activation in human keratinocytes

--Manuscript Draft--

**Manuscript Number:**	PONE-D-25-63339R1
**Article Type:**	Research Article
**Full Title:**	Hydrolyzed corn starch with maltotetraose for skin defense through NRF2 pathway activation in human keratinocytes
**Short Title:**	Hydrolyzed corn starch with maltotetraose induced antioxidative responses via NRF2 pathway
**Corresponding Author:**	Hitomi Hagawa<br>Lion Corporation<br>Tokyo, JAPAN
**Keywords:**	
**Abstract:**	Oxidative stress, which can be triggered by various external stimuli, such as ultraviolet radiation and pollution, compromises skin health, accelerates aging and skin disorders, and impacts quality of life. This study investigated the potential of hydrolyzed corn starch containing maltotetraose in skin defense through nuclear factor erythroid 2-related factor 2 (NRF2) pathway activation in human keratinocytes exposed to oxidative stress. Using DNA microarray analysis and assessing key antioxidant-responsive genes, we evaluated the capacity of this ingredient to enhance cellular defense mechanisms. Human keratinocytes treated with it demonstrated significant upregulation of antioxidant-responsive genes, including HMOX1, GPX2, and NQO1, which are known targets of NRF2. These findings were corroborated by western blotting and immunostaining analyses, which confirmed increased NRF2 protein expression and NRF2 nuclear translocation. Moreover, fluorescence assays and microscopy showed that treatment with the ingredient effectively reduced reactive oxygen species levels in keratinocytes exposed to oxidative stress. These results suggest that activation of the NRF2 pathway by this ingredient enhances the cellular antioxidative response and reduces reactive oxygen species levels in keratinocytes. This NRF2-mediated antioxidative activity positions hydrolyzed corn starch containing maltotetraose as a promising ingredient for cosmetic products, with potential implications for improving skin health, reducing pigmentation, and combating aging. However, these effects were not directly evaluated in this study. Future studies are warranted to evaluate its efficacy in vivo.
**Order of Authors:**	Hitomi Hagawa
	Eri Ichikawa
	Keiko Otake
	Mayuko Ishii
	Naoko Kawaguchi
**Response to Reviewers:**	Journal Requirement (Editor)<br>1. Please ensure that your manuscript meets PLOS ONE's style requirements, including those for file naming. The PLOS ONE style templates can be found at https://journals.plos.org/plosone/s/file?id=wjVg/PLOSOne_formatting_sample_main_body.pdf and https://journals.plos.org/plosone/s/file?id=ba62/PLOSOne_formatting_sample_title_authors_affiliations.pdf.<br>>We have carefully revised the manuscript to ensure that it follows the PLOS ONE style requirements, including those for file naming and formatting. We have consulted and applied the formatting guidelines provided in the PLOS ONE style templates.<br>2. Thank you for stating in your Funding Statement: This research was supported financially by the Lion Corporation. No other specific grant was received from funding agencies in the public, commercial, or not-for-profit sectors.” Please provide an amended statement that declares *all* the funding or sources of support (whether

Powered by Editorial Manager® and ProduXion Manager® from Aries Systems Corporation

external or internal to your organization) received during this study, as detailed online in our guide for authors at http://journals.plos.org/plosone/s/submit-now. Please also include the statement “There was no additional external funding received for this study.” in your updated Funding Statement.
Please include your amended Funding Statement within your cover letter. We will change the online submission form on your behalf.
>We appreciate the editor’s guidance regarding the Funding Statement. We have revised the Funding Statement to read as follows, and will include this updated version in the cover letter:“This research was supported financially by the Lion Corporation. No other specific grant was received from funding agencies in the public, commercial, or not-for-profit sectors. There was no additional external funding received for this study.” We understand that the online submission form will be updated by the editorial office based on the text provided in the cover letter.
3. Thank you for stating the following financial disclosure:
“This research was supported financially by the Lion Corporation. No other specific grant was received from funding agencies in the public, commercial, or not-for-profit sectors.” Please state what role the funders took in the study. If the funders had no role, please state: "The funders had no role in study design, data collection and analysis, decision to publish, or preparation of the manuscript." If this statement is not correct you must amend it as needed. Please include this amended Role of Funder statement in your cover letter; we will change the online submission form on your behalf.
>We thank the editor for pointing out the need to clarify the role of the funder.
In accordance with the journal’s request, we have added the following statement regarding the role of the funder, which will be included in the cover letter:
"The funders had no role in study design, data collection and analysis, decision to publish, or preparation of the manuscript."
We understand that the editorial office will update the corresponding section in the online submission system based on this statement.
4. We note that you have indicated that there are restrictions to data sharing for this study. For studies involving human research participant data or other sensitive data, we encourage authors to share de-identified or anonymized data. However, when data cannot be publicly shared for ethical reasons, we allow authors to make their data sets available upon request. For information on unacceptable data access restrictions, please see http://journals.plos.org/plosone/s/data-availability#loc-unacceptable-data-access-restrictions. Before we proceed with your manuscript, please address the following prompts:
a) If there are ethical or legal restrictions on sharing a de-identified data set, please explain them in detail (e.g., data contain potentially identifying or sensitive patient information, data are owned by a third-party organization, etc.) and who has imposed them (e.g., a Research Ethics Committee or Institutional Review Board, etc.). Please also provide contact information for a data access committee, ethics committee, or other institutional body to which data requests may be sent.
b) If there are no restrictions, please upload the minimal anonymized data set necessary to replicate your study findings to a stable, public repository and provide us with the relevant URLs, DOIs, or accession numbers. Please see http://www.bmj.com/content/340/bmj.c181.long for guidelines on how to de-identify and prepare clinical data for publication. For a list of recommended repositories, please see https://journals.plos.org/plosone/s/recommended-repositories. You also have the option of uploading the data as Supporting Information files, but we would recommend depositing data directly to a data repository if possible.
Please update your Data Availability statement in the submission form accordingly.
>We have revised the Data Availability statement in the manuscript to more clearly describe where the data can be accessed. The revised statement reads as follows: However, the microarray data have not yet been assigned an accession number in GEO. The revised statement reads as follows:
“All microarray data files have been deposited in the NCBI Gene Expression Omnibus (GEO) under accession number GSEXXXXXX (to be updated once the accession number is assigned). All other datasets used for the statistical analyses, including individual data points and sample sizes for each experiment, have been deposited in the Zenodo repository and are freely available under the accession DOI 10.5281/zenodo.19227766. All other relevant data are within the manuscript and its Supporting Information files.” (line 372–380)
We hope that this revision sufficiently clarifies the sample sizes and the accessibility of

all datasets used in our analyses and ensures compliance with the PLOS ONE Data Availability policy.
5. In the online submission form you indicate that your data is not available for proprietary reasons and have provided a contact point for accessing this data. Please note that your current contact point is a co-author on this manuscript. According to our Data Policy, the contact point must not be an author on the manuscript and must be an institutional contact, ideally not an individual. Please revise your data statement to a non-author institutional point of contact, such as a data access or ethics committee, and send this to us via return email. Please also include contact information for the third party organization, and please include the full citation of where the data can be found.
>In response to the journal's data policy, we have revised our data sharing plan so that the underlying datasets are now publicly available and no longer subject to proprietary access restrictions.
As noted above, the microarray data have been submitted to the NCBI Gene Expression Omnibus (GEO) and will be available under accession GSEXXXXXX once it is assigned. All other datasets used for statistical analyses have been deposited in Zenodo (DOI 10.5281/zenodo.19227766). Therefore, we no longer rely on a "data available upon request" model, and no author will serve as a direct data-access contact point.
Because the data are openly available through these public repositories, an additional institutional contact point for data access is no longer required.
6. Please be informed that funding information should not appear in the Acknowledgments section or other areas of your manuscript. We will only publish funding information present in the Funding Statement section of the online submission form. Please remove any funding-related text from the manuscript.
>We acknowledge the journal's policy that funding information should only appear in the Funding Statement section of the online submission form and not in the Acknowledgments or other parts of the manuscript. Accordingly, we have removed all funding-related text from the Acknowledgments section and from any other location in the manuscript.
7. We note that you have included the phrase "data not shown" in your manuscript. Unfortunately, this does not meet our data sharing requirements. PLOS does not permit references to inaccessible data. We require that authors provide all relevant data within the paper, Supporting Information files, or in an acceptable, public repository. Please add a citation to support this phrase or upload the data that corresponds with these findings to a stable repository (such as Figshare or Dryad) and provide and URLs, DOIs, or accession numbers that may be used to access these data. Or, if the data are not a core part of the research being presented in your study, we ask that you remove the phrase that refers to these data.
>We thank the editor and reviewers for drawing our attention to the use of the phrase "data not shown" and for emphasizing the importance of data transparency. In response to the concern that "data not shown" is not acceptable for key results and that the underlying data must be available, we have now provided the supporting microarray data for the 73 selected probes as a supplementary file (S1 Table). These changes ensure that all relevant data supporting our findings are accessible to readers and fully comply with PLOS ONE's data sharing requirements.
8. PLOS ONE now requires that authors provide the original uncropped and unadjusted images underlying all blot or gel results reported in a submission's figures or Supporting Information files. This policy and the journal's other requirements for blot/gel reporting and figure preparation are described in detail at https://journals.plos.org/plosone/s/figures#loc-blot-and-gel-reporting-requirements and https://journals.plos.org/plosone/s/figures#loc-preparing-figures-from-image-files. When you submit your revised manuscript, please ensure that your figures adhere fully to these guidelines and provide the original underlying images for all blot or gel data reported in your submission. See the following link for instructions on providing the original image data: https://journals.plos.org/plosone/s/figures#loc-original-images-for-blots-and-gels. In your cover letter, please note whether your blot/gel image data are in Supporting Information or posted at a public data repository, provide the repository URL if relevant, and provide specific details as to which raw blot/gel images, if any, are not available. Email us at plosone@plos.org if you have any questions.
>We understand and appreciate the journal's policy requiring the submission of original, uncropped, and unadjusted images for all blot and gel data. In accordance with this policy, we have prepared and submitted the raw Western blot images

Powered by Editorial Manager® and ProduXion Manager® from Aries Systems Corporation

	underlying Figure 4 as Supporting information (S1_raw_images.pdf). These files correspond directly to the processed images shown in Figure 4. We will clearly indicate in the cover letter that the raw blot images for Figure 4 have been provided, and we confirm that there are no additional blot or gel images in the manuscript for which raw data are unavailable.<br>9. If the reviewer comments include a recommendation to cite specific previously published works, please review and evaluate these publications to determine whether they are relevant and should be cited. There is no requirement to cite these works unless the editor has indicated otherwise.<br>>We note that the reviewers did not explicitly recommend any specific previously published works that must be cited in this manuscript. Accordingly, no reviewer-suggested citations needed to be added. We have, however, reviewed and updated the references in the Introduction and Discussion where appropriate to ensure that the most relevant and recent literature is cited.<br><br>Reviewer #1<br>1. Repetition of the phrase “hydrolyzed corn starch containing maltotetraose” affects flow and should be reduced in the Abstract section.<br>>We appreciate this helpful comment. In the revised Abstract, we now use the full expression “hydrolyzed corn starch containing maltotetraose” only when the ingredient is first introduced and again in the concluding sentence where we summarize its significance. In the rest of the Abstract, we refer to it more concisely as “this ingredient (line: 24 and 32),” “it,” (line: 25) or “the ingredient” (line: 29-30) where the meaning is clear. We believe this has reduced redundancy and improved the readability of the Abstract.<br>2. No pigmentation-related markers, melanocyte models, or aging-associated endpoints are mentioned.<br>>We appreciate insightful comments. We fully agree that our current study did not include pigmentation-related markers, melanocyte-based models, or aging-associated endpoints, and that any statements regarding pigmentation or aging must therefore be made with caution.<br>In response to this comment, we have revised the Abstract and Discussion to clearly state that the data presented in this study are limited to antioxidant responses and NRF2 activation in keratinocytes. Any potential implications for pigmentation or aging are now explicitly described as hypothetical and not directly evaluated in this work. In the Abstract we now state:<br>“This NRF2-mediated antioxidative activity positions hydrolyzed corn starch containing maltotetraose as a promising ingredient for cosmetic products, with potential implications for improving skin health, reducing pigmentation, and combating aging. However, these effects were not directly evaluated in this study. Future studies are warranted to evaluate its efficacy in vivo.” (line: 33-38)<br>In the Discussion, we also note that:<br>“However, our current data are limited to antioxidant responses in keratinocytes, and we did not directly assess structural or functional endpoints (e.g., collagen content, skin elasticity, melanin production) in the skin. Therefore, any anti-wrinkle, anti-sagging, or depigmenting effects remain speculative and should be validated in future in vitro and clinical studies.” (line: 324-328)<br>We hope that these revisions clarify the scope and limitations of our study.<br>3. In line 32, the phrase “novel functionality” is vague and should be replaced with more precise scientific language.<br>>We appreciate this helpful comment. We agree that the term “novel functionality” was vague. In the revised manuscript, we have replaced this phrase with the more specific and scientifically accurate term “NRF2-mediated antioxidative activity,” (line: 34) which directly reflects the mechanism demonstrated in our study. The sentence now reads:<br>“This NRF2-mediated antioxidative activity positions hydrolyzed corn starch containing maltotetraose as a promising ingredient for cosmetic products, with potential implications for improving skin health, reducing pigmentation, and combating aging.” (line: 33-36)<br>This revision clarifies that the functionality in question is the activation of the NRF2 pathway leading to an enhanced antioxidative response.<br>4. Typographical error identified: “maltotetoraose” should be corrected to “maltotetraose” in line 33.<br>>We thank the reviewer for pointing out this typographical error. In the revised manuscript, we have corrected the misspelling “maltotetoraose” to “maltotetraose” on

	line 35. We have also checked the entire manuscript to ensure that the term "maltotetraose" is spelled consistently throughout.<br>5. In line 56, the statement that NRF2 is a "promising target in cosmetic applications" is plausible but under-argued. Clarify why moderate NRF2 activation is beneficial and safe in cosmetic formulations.<br>>We appreciate this valuable comment. We agree that additional explanation was needed to clarify why NRF2 can be regarded as a beneficial and safe target in the context of cosmetic formulations.<br>In the revised Introduction, we have elaborated on the role of NRF2 in skin and the rationale for targeting it in cosmetics. (line: 59-67) Specifically, we now emphasize that:<br>•NRF2 regulates a broad range of antioxidant and phase II detoxification enzymes that detoxify and eliminate reactive oxygen species and other oxidative agents, thereby contributing to photoprotection, regulation of pigmentation, wound healing, and reinforcement of epidermal barrier function [3,5-7]. Enhancement of NRF2 signaling is therefore expected to strengthen endogenous antioxidant and barrier defenses, thereby helping to prevent photoaging, pigmentation disorders, and barrier impairment induced by environmental stressors.<br>•Transient NRF2 activation is unlikely to be harmful, as NRF2 upregulates Keap1 expression via a negative feedback mechanism that contributes to the maintenance of physiological redox balance in cells.<br>We hope that this clarification addresses the concern regarding why moderate NRF2 activation can be considered both beneficial and safe in cosmetic formulations.<br>6. "Raw and functional cosmetic ingredient" is vague. Consider replacing with "naturally derived functional cosmetic ingredient" in line 57.<br>>Thank you for pointing this out. We agree that the previous wording was ambiguous. In the revised manuscript, we have replaced "raw and functional cosmetic ingredient" with a clearer description. The relevant sentence now reads: "We have identified hydrolyzed corn starch, derived from corn, as a safe and effective naturally derived functional cosmetic ingredient." (line: 71-72)<br>This wording better reflects both the natural origin and the functional role of the ingredient in cosmetic formulations.<br>7. The assertion that MTO has "superior skin-moisturizing properties" based on hydroxyl group content alone is chemically simplistic. Provide experimental or literature-based evidence supporting superior humectancy, or rephrase to a more cautious claim (e.g., "potentially enhanced moisturizing capacity") in line 60.<br>>We fully agree that our initial wording was too strong given that it was based only on the number of hydroxyl groups. In accordance with your suggestion, we have revised this part of the text to adopt a more cautious wording. The sentence now reads: "Given its higher concentration of hydroxyl groups compared to other humectants, such as glycerin and butylene glycol, MTO has potentially enhanced moisturizing capacity." (line: 74-76)<br>By using the phrase "potentially enhanced moisturizing capacity," we avoid overinterpreting the chemical structure alone and clearly indicate that superior moisturizing efficacy is a possibility rather than an established fact.<br>Where relevant, we refer to our clinical study demonstrating moisturizing and barrier-improving effects of an MTO-containing moisturizer [13], which supports the practical usefulness of MTO in skin hydration without attributing this solely to the number of hydroxyl groups.<br>8. In line 184, the term "robes" appears to be a typographical error and should be corrected to "probes". The phrase "data not shown" is not acceptable for a primary result in a manuscript. Transparency requires that the underlying data be accessible.<br>>We thank the reviewer for carefully pointing out the typographical error and the issue regarding data transparency.<br>First, the spelling error "robes" has been corrected to "probes" in the revised manuscript. (line: 219)<br>Second, in response to the reviewer's comment that "data not shown" is not acceptable for major results and that the underlying data should be accessible, we have now provided the supporting microarray data (S1 Table) for the selected 73 probes. (line: 219)<br>9. Reporting only the number of selected probes (n = 73) without presenting any supporting data (e.g., fold changes, adjusted p-values, or gene identities) is insufficient. At a minimum, a supplementary table should be provided.<br>>We have added S1 Table, which lists, for each of the 73 probes, the gene name, Unigene, fold change, and p-value. We believe these changes improve the

Powered by Editorial Manager® and ProduXion Manager® from Aries Systems Corporation

transparency and reproducibility of our analysis and adequately address the reviewer's concern.

10. The discussion appropriately outlines the NRF2–ARE antioxidant pathway and its relevance to skin oxidative stress. However, the conclusion that hydrolyzed corn starch mitigates wrinkles, sagging, and pigmentation is speculative and not directly supported by the presented data.

>We agree that our original wording could be interpreted as claiming direct evidence for anti-wrinkle, anti-sagging, and depigmenting effects, which is not supported by our current dataset. Accordingly, we have revised the Discussion to clearly distinguish between our experimental findings and potential implications.

The following paragraphs were added:

"Given that excessive ROS are known to contribute to collagen and elastin degradation and to hyperpigmentation through the activation of melanocyte-stimulation factors [16-19], NRF2 activation and ROS reduction by hydrolyzed corn starch raise the possibility that this ingredient may indirectly influence skin conditions such as wrinkles, sagging, and pigmentation. However, our current data are limited to antioxidant responses in keratinocytes, and we did not directly assess structural or functional endpoints (e.g., collagen content, skin elasticity, melanin production) in the skin. Therefore, any anti-wrinkle, anti-sagging, or depigmenting effects remain speculative and should be validated in future in vitro and clinical studies. In addition, our experiments were conducted exclusively in keratinocytes and did not address the role of dermal fibroblasts, which are crucial for extracellular matrix production and skin elasticity. Future investigations using dermal fibroblasts, melanocyte-keratinocyte co-culture systems, and in vivo or clinical evaluations will be required to clarify whether the NRF2 activation and ROS reduction observed here translate into measurable improvements in these skin conditions." (line: 321-332)

11. Justify this statement: the anti-inflammatory effect is inferred solely from literature citing NRF2-mediated suppression of IL-6 and IL-1β. Since no inflammatory markers were measured in this study, this conclusion should be framed as a potential implication, not as an observed effect in lines 265-268.

>We fully concur with this comment. In the revised Discussion, we explicitly state that any anti-inflammatory effect is hypothetical and derived from known NRF2 functions, not from our own measurements. The relevant part now reads:

"Moreover, recent studies have demonstrated that activation of the NRF2 pathway can suppress inflammation by inhibiting the expression of interleukin 6 and interleukin 1β, cytokines that exacerbate inflammation [16]. On the basis of these reports, the NRF2 activation observed in our study raises the possibility that hydrolyzed corn starch may have anti-inflammatory effects on the skin. However, we did not measure inflammatory markers, cytokine expression, or other indices of inflammation in our experiments. Thus, any anti-inflammatory effect of hydrolyzed corn starch should be regarded as a hypothesis derived from the known function of NRF2 rather than as an observed effect." (line: 340-346)

Reviewer #2

1. Please provide latest references for the second paragraph of the Introduction section.

>We appreciate your suggestion to include more recent references supporting the role of NRF2 in skin biology and pathology. In the revised version of the Introduction (second paragraph, line: 50-67), we have added the following key references:

[5] Schäfer M, Werner S. Nrf2-A regulator of keratinocyte redox signaling. Free Radical Biology and Medicine. 2015;88: 243-252

[6] Salman S, Paulet V, Hardonniére K, Kerdine-Römer S. The role of NRF2 transcription factor in inflammatory skin diseases. Biofactors. 2025;51(2)

[7] Kim HJ, Hong JH. Emerging Therapeutic Strategies for Nrf2-Associated Skin Disorders: From Photoaging to Autoimmunity. Antioxidants. 2026;15(1): 69

These references have been incorporated into the second paragraph of the Introduction to better substantiate the description of NRF2 as a central regulator of cutaneous antioxidant and cytoprotective responses, and to align the manuscript with the latest advances in the field.

2. If possible please provide a schematic to explain the pathways during presence and absence of oxidative stress.

>Thank you for this helpful suggestion. In the revised manuscript, we have added a schematic illustration of the NRF2–Keap1 pathway under basal and oxidative stress conditions.

Powered by Editorial Manager® and ProduXion Manager® from Aries Systems Corporation

The new figure is included as Figure 1 and is described as follows:
"Fig 1. NRF2-Keap1 pathway under basal and oxidative stress conditions.
Under basal conditions, NRF2 is retained in the cytoplasm through its interaction with Keap1, which promotes NRF2 ubiquitination and proteasomal degradation. In the presence of oxidative stress, structural changes in Keap1 lead to the release and stabilization of NRF2. The stabilized NRF2 translocates into the nucleus, where it binds to ARE in the promoters of target genes. This induces the expression of phase II detoxification enzymes, antioxidant enzymes, and other cytoprotective molecules." (line: 90-96)
We believe this schematic will facilitate readers' understanding of the molecular mechanism by which NRF2 contributes to the skin's defense against oxidative stress and will clarify the rationale for targeting NRF2 in our study
3. Is there previously available literature that supports hydrolyzed corn starch or MTO f as a potent cosmetic ingredient?
>Thank you for this important comment. We agree that the basis for describing hydrolyzed corn starch as a promising cosmetic ingredient should be clarified.
In the revised manuscript, we have added one sentence in the Introduction to explicitly indicate the official cosmetic classification of hydrolyzed corn starch:
"Hydrolyzed corn starch is officially categorized as a skin conditioning agent, specifically a humectant, in cosmetic ingredient nomenclature [8]." (line: 70-71)
We hope that this clarification in the Introduction, sufficiently addresses the reviewer's concern regarding the existing evidence supporting hydrolyzed corn starch as a cosmetic ingredient.
4. The data of 73 probes can be provided as supplementary information?
>We agree with the reviewer that the data for the 73 selected probes should be made available as supplementary information. In the revised manuscript, we have therefore added Supplementary S1 Table, which provides, for each of the 73 probes, the gene name, Unigene, fold change, and p-value. The corresponding text in the Results section has also been updated to explicitly refer to S1 Table instead of "data not shown." (line: 219)
We hope that this supplementary table sufficiently addresses the reviewer's request for access to the underlying data.
5. Need more clarity on the sample size and availability of the statistical analysis data.
>We appreciate the reviewer's comment regarding the need for clearer information on sample size and statistical analyses. In the revised manuscript, we have clarified the sample size (n) and statistical methods as follows.
•For the DNA microarray analysis, we now specify that "the CEL files obtained from three independent samples per condition (n = 3)". (line: 126-127)
•For the gene expression and western blotting experiments, we now state that " Three replicate wells were evaluated for each group (n = 3)" in the Materials and Methods sections (line: 159, 170, 193).
•The figure legends (e.g., Fig 2 (line: 247-254)) have been revised to indicate the statistical test used (Dunnett's test) and the significance thresholds, together with the sample size (n = 3).
•We have revised the Data Availability statement in the manuscript as follows:
"All microarray data files have been deposited in the NCBI Gene Expression Omnibus (GEO) under accession number GSEXXXXXX (to be updated once the accession number is assigned). All other datasets used for the statistical analyses, including individual data points and sample sizes for each experiment, have been deposited in the Zenodo repository and are freely available under the accession DOI 10.5281/zenodo.19227766. All other relevant data are within the manuscript and its Supporting Information files." (line: 372-380)
We hope that this revision sufficiently clarifies the sample sizes and the accessibility of all datasets used in our analyses.

Reviewer #3
1. The bar graphs are not properly labeled on the x-axis, and statistical significance is not indicated.
>We appreciate the reviewer's valuable comment regarding the x-axis labels and the indication of statistical significance.
In response, we have revised the figures as follows:
Fig 2: We performed a Dunnett's multiple comparison test and have now indicated statistically significant differences on the graph. In addition, we adjusted the positions of the x-axis labels to improve clarity and readability.

Fig 3: We conducted a Student's t-test and have added the corresponding statistical significance to the graph. Similarly, we optimized the placement of the x-axis labels in this figure.
We believe these revisions address the reviewer's concerns and improve the quality and interpretability of the figures.
2. The authors claim that maltotetraose is a promising ingredient for cosmetic applications with potential roles in improving skin health, reducing pigmentation, and combating aging. However, no experimental data are provided to support claims related to pigmentation reduction or anti-aging effects. Moreover, the experiments were conducted exclusively in keratinocytes. The role of dermal fibroblasts, which are critical for maintaining skin elasticity and extracellular matrix integrity, has not been addressed.
>We appreciate this important comment and have revised the Discussion to avoid overstatement and to clarify the limitations of our study design. We now explicitly acknowledge that our data are restricted to keratinocyte responses and do not include fibroblast or pigmentation endpoints. The following statements were added:
"Given that excessive ROS are known to contribute to collagen and elastin degradation and to hyperpigmentation through the activation of melanocyte-stimulation factors [16-19], NRF2 activation and ROS reduction by hydrolyzed corn starch raise the possibility that this ingredient may indirectly influence skin conditions such as wrinkles, sagging, and pigmentation. However, our current data are limited to antioxidant responses in keratinocytes, and we did not directly assess structural or functional endpoints (e.g., collagen content, skin elasticity, melanin production) in the skin. Therefore, any anti-wrinkle, anti-sagging, or depigmenting effects remain speculative and should be validated in future in vitro and clinical studies. In addition, our experiments were conducted exclusively in keratinocytes and did not address the role of dermal fibroblasts, which are crucial for extracellular matrix production and skin elasticity. Future investigations using dermal fibroblasts, melanocyte-keratinocyte co-culture systems, and in vivo or clinical evaluations will be required to clarify whether the NRF2 activation and ROS reduction observed here translate into measurable improvements in these skin conditions." (line: 321-332)
Furthermore, we have revised the description of maltotetraose (MTO) so that it is no longer presented as a definitively "promising" cosmetic ingredient, but rather positioned as a mechanistic hypothesis based on the current data.
"Our results also suggest that maltotetraose (MTO), a major component of hydrolyzed corn starch, may play a key role in NRF2 activation and subsequent ROS reduction in keratinocytes, although the detailed mechanism remains unclear. Whether ROS reduction is solely mediated by the NRF2 pathway and the specific mechanism by which MTO activates NRF2 are not yet established. In addition, we did not evaluate the overall status of enzymatic and non-enzymatic antioxidant capacity in this study, and thus the global impact of MTO on the cutaneous antioxidant defense system remains unclear. A comprehensive understanding will require the identification of the individual compounds in the hydrolyzed corn starch, subsequent validation using each individual compound, and elucidation of the associated molecular interactions and signaling pathways with specific inhibitors." (line: 350-359)
3. The discussion section requires substantial improvement to better interpret the findings and place them in the context of existing literature.
>We have reorganized the Discussion to follow a more logical progression:
(1) overview of NRF2–ARE signaling and ROS biology in the skin
(2) summary of our key findings (NRF2 activation, gene expression, and ROS reduction in keratinocytes)
(3) additional findings (expression of other antioxidant related genes)
(4) cautious discussion of potential implications for skin aging and pigmentation, explicitly framed as hypotheses
(5) clarification of limitations, including the lack of data on fibroblasts, extracellular matrix, pigmentation, and inflammatory markers
(6) future directions, including the need for compound-level analyses, mechanistic studies with inhibitors, and clinical validation.
4. The authors evaluated NRF2-associated antioxidant response genes, including HMOX1, GPX2, and NQO1. However, the effects of maltotetraose on other antioxidant systems, such as PRDX1, as well as overall enzymatic and non-enzymatic antioxidant status, were not investigated.
>We appreciate the reviewer's suggestion to further investigate the broader antioxidant system beyond NRF2-related genes such as HMOX1, GPX2, and NQO1. In response,

	we performed additional analyses of antioxidant-related gene expression. Specifically, we quantified the mRNA expression levels of peroxiredoxin 1 (PRDX1), superoxide dismutase 1 (SOD1), catalase (CAT), glutathione reductase (GSR), glutamate-cysteine ligase catalytic subunit (GCLC), and thioredoxin (TXN) by RT-qPCR after treatment with 1.0%, 2.0%, and 4.0% hydrolyzed corn starch for 24 h. As shown in the revised Results section and Fig. S1, GCLC and TXN were upregulated in a concentration-dependent manner, and PRDX1 was significantly increased at 4.0%, whereas SOD1, CAT, and GSR showed no significant changes.<br>These findings have been incorporated into the Results (line: 238-244) and discussed in the Discussion section (line: 311-320). In addition, we explicitly noted in the revised Discussion that "we did not evaluate the overall status of enzymatic and non-enzymatic antioxidant capacity in this study, and thus the global impact of maltotetraose on the cutaneous antioxidant defense system remains unclear." (line: 354-356)
**Additional Information:**	
**Question**	**Response**
**Financial Disclosure**<br><br>Enter a financial disclosure statement that describes the sources of funding for the work included in this submission. Review the submission guidelines for detailed requirements. View published research articles from *PLOS ONE* for specific examples.<br><br>This statement is required for submission and **will appear in the published article** if the submission is accepted. Please make sure it is accurate.<br><br>**Funded studies**<br>Enter a statement with the following details:<br>• Initials of the authors who received each award<br>• Grant numbers awarded to each author<br>• The full name of each funder<br>• URL of each funder website<br>• Did the sponsors or funders play any role in the study design, data collection and analysis, decision to publish, or preparation of the manuscript?<br><br>Did you receive funding for this work?	Yes
Please add funding details.<br>as follow-up to "**Financial Disclosure**<br><br>Enter a financial disclosure statement that describes the sources of funding for the work included in this submission. Review the submission guidelines for detailed	This research was supported financially by the Lion Corporation. No other specific grant was received from funding agencies in the public, commercial, or not-for-profit sectors.

requirements. View published research articles from *PLOS ONE* for specific examples.<br><br>This statement is required for submission and **will appear in the published article** if the submission is accepted. Please make sure it is accurate.<br><br>**Funded studies**<br>Enter a statement with the following details:<br>• Initials of the authors who received each award<br>• Grant numbers awarded to each author<br>• The full name of each funder<br>• URL of each funder website<br>• Did the sponsors or funders play any role in the study design, data collection and analysis, decision to publish, or preparation of the manuscript?<br><br>Did you receive funding for this work?"	
Please select the country of your main research funder (please select carefully as in some cases this is used in fee calculation).<br>as follow-up to "**Financial Disclosure**<br><br>Enter a financial disclosure statement that describes the sources of funding for the work included in this submission. Review the submission guidelines for detailed requirements. View published research articles from *PLOS ONE* for specific examples.<br><br>This statement is required for submission and **will appear in the published article** if the submission is accepted. Please make sure it is accurate.	JAPAN - JP

**Funded studies**<br>Enter a statement with the following details:<br>• Initials of the authors who received each award<br>• Grant numbers awarded to each author<br>• The full name of each funder<br>• URL of each funder website<br>• Did the sponsors or funders play any role in the study design, data collection and analysis, decision to publish, or preparation of the manuscript?<br><br>Did you receive funding for this work?"	
**Competing Interests**<br><br>Use the instructions below to enter a competing interest statement for this submission. On behalf of all authors, disclose any competing interests that could be perceived to bias this work—acknowledging all financial support and any other relevant financial or non-financial competing interests.<br><br>This statement is required for submission and **will appear in the published article** if the submission is accepted. Please make sure it is accurate and that any funding sources listed in your Funding Information later in the submission form are also declared in your Financial Disclosure statement.<br><br>View published research articles from *PLOS ONE* for specific examples.	The authors have declared that no competing interests exist.

**NO authors have competing interests**<br><br>Enter: *The authors have declared that no competing interests exist.*<br><br>**Authors with competing interests**<br><br>Enter competing interest details beginning with this statement:<br><br>*I have read the journal's policy and the authors of this manuscript have the following competing interests: [insert competing interests here]*<br><br>* typeset	
**Ethics Statement**<br><br>Enter an ethics statement for this submission. This statement is required if the study involved:<br><br>• Human participants<br>• Human specimens or tissue<br>• Vertebrate animals or cephalopods<br>• Vertebrate embryos or tissues<br>• Field research<br><br>Write "N/A" if the submission does not require an ethics statement.<br><br>General guidance is provided below. Consult the submission guidelines for detailed instructions. **Make sure that all information entered here is included in the Methods section of the manuscript.**	N/A

**Format for specific study types**<br><br>**Human Subject Research (involving human participants and/or tissue)**<br>• Give the name of the institutional review board or ethics committee that approved the study<br>• Include the approval number and/or a statement indicating approval of this research<br>• Indicate the form of consent obtained (written/oral) or the reason that consent was not obtained (e.g. the data were analyzed anonymously)<br><br>**Animal Research (involving vertebrate animals, embryos or tissues)**<br>• Provide the name of the Institutional Animal Care and Use Committee (IACUC) or other relevant ethics board that reviewed the study protocol, and indicate whether they approved this research or granted a formal waiver of ethical approval<br>• Include an approval number if one was obtained<br>• If the study involved *non-human primates*, add *additional details* about animal welfare and steps taken to ameliorate suffering<br>• If anesthesia, euthanasia, or any kind of animal sacrifice is part of the study, include briefly which substances and/or methods were applied<br><br>**Field Research**<br><br>Include the following details if this study involves the collection of plant, animal, or other materials from a natural setting:<br>• Field permit number<br>• Name of the institution or relevant body that granted permission	
**Data Availability**<br><br>Authors are required to make all data underlying the findings described fully available, without restriction, and from the time of publication. PLOS allows rare exceptions to address legal and ethical concerns. See the PLOS Data Policy and FAQ for detailed information.	Yes - all data are fully available without restriction

A Data Availability Statement describing where the data can be found is required at submission. Your answers to this question constitute the Data Availability Statement and **will be published in the article**, if accepted.<br><br>**Important:** Stating 'data available on request from the author' is not sufficient. If your data are only available upon request, select 'No' for the first question and explain your exceptional situation in the text box.<br><br>Do the authors confirm that all data underlying the findings described in their manuscript are fully available without restriction?	
**Describe where the data may be found in full sentences. If you are copying our sample text, replace any instances of XXX with the appropriate details.**<br><br>• If the data are **held or will be held in a public repository**, include URLs, accession numbers or DOIs. If this information will only be available after acceptance, indicate this by ticking the box below. For example: *All XXX files are available from the XXX database (accession number(s) XXX, XXX.)*.<br>• If the data are all contained **within the manuscript and/or Supporting Information files**, enter the following: *All relevant data are within the manuscript and its Supporting Information files.*<br>• If neither of these applies but you are able to provide **details of access elsewhere**, with or without limitations, please do so. For example:<br><br>*Data cannot be shared publicly because of [XXX]. Data are available from the XXX Institutional Data Access / Ethics Committee (contact via XXX) for researchers who meet the criteria for access to confidential data.*<br><br>*The data underlying the results presented in the study are available from (include the name of the third party*	All microarray data files have been deposited in the NCBI Gene Expression Omnibus (GEO) under accession number GSEXXXXXX (to be updated once the accession number is assigned). All other datasets used for the statistical analyses, including individual data points and sample sizes for each experiment, have been deposited in the Zenodo repository and are freely available under the accession DOI 10.5281/zenodo.19227766. All other relevant data are within the manuscript and its Supporting Information files.

and contact information or URL).<br>• This text is appropriate if the data are owned by a third party and authors do not have permission to share the data.<br><br>* typeset	
Additional data availability information:	

Powered by Editorial Manager® and ProduXion Manager® from Aries Systems Corporation

Manuscript Click here to access/download;Manuscript;Manuscript.docx

Hydrolyzed corn starch with maltotetraose for skin defense through NRF2 pathway activation in human keratinocytes

Short title: Hydrolyzed corn starch with maltotetraose induced antioxidative responses via NRF2 pathway

Hitomi Hagawa[1*], Eri Ichikawa[2], Keiko Otake[1], Mayuko Ishii[1], Naoko Kawaguchi[1]

[1] Global Development Center, Research & Development Headquarters, Lion Corporation, Edogawa-ku, Tokyo, Japan

[2] Northeast Asia Business Division, Business Development Department 1, Lion Corporation, Tokyo, Japan

* Corresponding author:

E-mail: hagawa-h@lion.co.jp (HH)

Abstract

Oxidative stress, which can be triggered by various external stimuli, such as ultraviolet radiation and pollution, compromises skin health, accelerates aging and skin disorders, and impacts quality of life. This study investigated the potential of hydrolyzed corn starch containing maltotetraose in skin defense through nuclear factor erythroid 2-related factor 2 (NRF2) pathway activation in human keratinocytes exposed to oxidative stress. Using DNA microarray analysis and assessing key antioxidant-responsive genes, we evaluated the capacity of this ingredient to enhance cellular defense mechanisms. Human keratinocytes treated with it demonstrated significant upregulation of antioxidant-responsive genes, including *HMOX1*, *GPX2*, and *NQO1*, which are known targets of NRF2. These findings were corroborated by western blotting and immunostaining analyses, which confirmed increased NRF2 protein expression and NRF2 nuclear translocation. Moreover, fluorescence assays and microscopy showed that treatment with the ingredient effectively reduced reactive oxygen species levels in keratinocytes exposed to oxidative stress. These results suggest that activation of the NRF2 pathway by this ingredient enhances the cellular antioxidative response and reduces reactive oxygen species levels in keratinocytes. This NRF2-mediated antioxidative activity positions hydrolyzed corn starch containing maltotetraose as a promising ingredient for cosmetic products, with potential implications for improving skin health, reducing pigmentation, and combating aging. However, these effects were not directly evaluated in this study. Future studies are warranted to evaluate its efficacy *in vivo.*

Introduction

The skin serves as the interface between the external and internal environments and is continuously exposed to various external stimuli, including ultraviolet radiation, air pollution, tobacco smoke, and alcohol [1-3]; these stimuli generate reactive oxygen species (ROS). Excessive or prolonged exposure to ROS leads to oxidative stress [4], which in turn exacerbates skin pigmentation and aging, ultimately resulting in uneven skin tone, hyperpigmentation, roughness, sagging, and wrinkles. This may lead to cosmetic imperfections and progress to severe dryness, sensitivity, and more severe conditions, such as atopic dermatitis, psoriasis, and skin cancer, thereby significantly impacting quality of life. Considering that the skin is continuously subjected to oxidative stress, potent antioxidant defenses are essential, especially for low-barrier or sensitive skin, which is particularly susceptible to external stimuli.

The skin possesses a robust antioxidant defense system, with the transcription factor nuclear factor erythroid 2-related factor 2 (NRF2) playing a central role by regulating the expression of a wide array of antioxidant enzymes responsible for detoxifying and eliminating oxidative agents. Under basal conditions, NRF2 is maintained in an inactive state in the cytoplasm through its binding to Kelch-like ECH-associated protein 1 (Keap1) and is directed toward proteasomal degradation. Under oxidative stress, NRF2 is released from Keap1 and translocates into the nucleus, where it binds to the antioxidant response element (ARE). This interaction modulates key skin defense mechanisms, including phase II detoxification, inflammatory signaling, DNA repair, and antioxidant responses (Fig 1) [5, 6]. In the skin, the NRF2 pathway has been implicated in photoprotection, regulation of pigmentation, wound healing, and reinforcement of the epidermal barrier function [3, 7]. From a cosmetic perspective, enhancement of NRF2 signaling is therefore expected to strengthen endogenous antioxidant and barrier defenses, thereby helping to prevent photoaging, pigmentation disorders, and barrier impairment induced by environmental stimuli. Moreover, transient NRF2 activation is unlikely to be harmful, as NRF2 upregulates Keap1 expression via a negative feedback mechanism that contributes

to the maintenance of physiological redox balance in cells [3]. Consequently, incorporating NRF2 modulators into cosmetic formulations can be viewed as a promising strategy to protect the skin from environmental stress and to maintain skin homeostasis.

Hydrolyzed corn starch is officially categorized as a skin conditioning agent, specifically a humectant, in cosmetic ingredient nomenclature [8]. We have identified hydrolyzed corn starch, derived from corn, as a safe and effective naturally derived functional cosmetic ingredient. This starch mainly contains maltotetraose (MTO), a polymer of glucose linked by α-1,4-glycosidic bond. Given its higher concentration of hydroxyl groups compared to other humectants, such as glycerin and butylene glycol, MTO has potentially enhanced moisturizing capacity. Moisturization is a fundamental aspect of skin care; for low-barrier and sensitive skin, application of moisturizers is crucial for restoring the skin barrier [9]. In addition, the itch–scratch cycle, observed in sensitive skin, can significantly impair quality of life. Our previous studies have demonstrated that MTO possesses notable itch-suppressing effects. Specifically, MTO significantly suppresses chronic itching by modulating mechanisms involving substance P and the neurokinin 1 receptor (NK1R) [10, 11], notably through inhibition of NK1R activation [12]. Based on these findings, we developed an itch-relief moisturizer containing MTO. A clinical trial involving patients with dry, itchy, and sensitive skin demonstrated that treatment with MTO effectively moisturized the skin, enhanced the skin barrier, and reduced itching [13]. Therefore, MTO holds promise as a functional component in the cosmetic field.

In this study, we aimed to investigate the other potential applications of MTO as a cosmetic ingredient by exploring its novel functionalities using human epidermal skin cells. Specifically, we examined the possibility of enhancing the skin's ability to defend against oxidative stress by activating NRF2 and reducing ROS.

Fig 1. NRF2-Keap1 pathway under basal and oxidative stress conditions.

Under basal conditions, NRF2 is retained in the cytoplasm through its interaction with Keap1, which promotes NRF2 ubiquitination and proteasomal degradation. In the presence of oxidative stress,

structural changes in Keap1 lead to the release and stabilization of NRF2. The stabilized NRF2 translocates into the nucleus, where it binds to ARE in the promoters of target genes. This induces the expression of phase II detoxification enzymes, antioxidant enzymes, and other cytoprotective molecules.

Materials and methods

Ethics Statement

Normal Human Epidermal Keratinocytes (NHEK; adult, single donor) were purchased from PromoCell GmbH (Heidelberg, Germany; Cat. No. C-12003; Lot Nos. 489Z018.2, 470Z025.1, 479Z032.1). PromoCell obtains donor tissues only after written informed consent and in accordance with the Declaration of Helsinki, providing fully anonymized samples (https://www.promocell.com/ethics). Under Lion Corporation Institutional Review Board (IRB) policy, research readily obtainable from commercial sources is exempt from additional review.

Cell culture

Human keratinocytes (NHEKs; PromoCell, Cat. No. C-12003) were purchased from Takara Bio Inc. (Shiga, Japan; Cat. No. C-12003) and cultured in Keratinocyte Growth Medium 2 (PromoCell, Cat. No. C-20011) containing 5 μg/mL insulin, 0.125 ng/mL human recombinant epidermal growth factor, 0.33 μg/mL hydrocortisone, 0.39 μg/mL epinephrine, 10 μg/mL holo-transferrin, 0.06 mM $CaCl_2$ and 0.004 ml/mL bovine pituitary extract at 37°C in a humidified atmosphere of 5% CO_2. Hydrolyzed corn starch (Leogard MT; Lion Specialty Chemicals Co., Ltd., Tokyo, Japan) was added to dissolve in the medium with NHEKs.

DNA microarray analysis

NHEKs were seeded and grown in six-well plates until they were subconfluent in a 37°C, 5% CO_2 incubator. Then, the control samples (only the medium) and 2.0% hydrolyzed corn starch in the medium were added, and after culture for 24 h, RNeasy Plus Mini Kit (Qiagen, Hilden, Germany) was

used to extract RNA from the cells following the manufacturer's protocol. The quality and quantity of the total RNA were evaluated on an Agilent 2100 Bioanalyzer (Agilent Technologies Japan, Ltd., Tokyo, Japan) and the total RNA integrity number exceeded seven. These total RNA samples were subjected to DNA microarray analysis using a Clariom™ S Array, human (Thermo Fisher Scientific Inc., MA, USA) by Kurabo (Osaka, Japan), and the fluorescence signals were scanned with a Biosystems™ GeneChip Scanner 3000 7G System. Biosystems™ GeneChip Command Console Software ver. 3.2 was used to reduce the array images to the intensity values for each probe (CEL files).

DNA microarray data analysis

Using R software (ver. 4.3.2; The R Project, https://www.r-project.org/), the CEL files obtained from three independent samples per condition (n=3) were quantified according to the distribution Robust Multichip Analysis. A statistical comparison of the control and 2.0% hydrolyzed corn starch was performed with the Rank Products and Benjamini and Hochberg false discovery rate (FDR) corrections. The Human Genome Array annotation file was used (Clariom™ S Array, human Transcript Cluster Annotations, CSV, Release 36). Differentially expressed genes were extracted on setting the values of $p < 0.15$, and the Database for Annotation, Visualization, and Integrated Discovery were used to classify the selected probe sets according to functional annotations. The functional analysis was performed on the basis of the molecular function in UniProtKB(UP)_Keywords(KW)_MF. In addition, while comparing cells treated with 2.0% hydrolyzed corn starch and control cells, genes that exhibited signal intensities of 20% to 100% with a fold change of ≥2.0 were considered upregulated. A significant difference was defined as an FDR-corrected value of $p < 0.05$.

Gene-expression analysis

NHEKs were seeded and grown in six-well plates until they were subconfluent in a 37°C, 5% CO_2

incubator. Then, the samples of control and 1.0%, 2.0% and 4.0% hydrolyzed corn starch were added, and after culturing for 3, 6, 10, 24, and 48 h, an RNeasy Plus Mini Kit (Qiagen) was used to extract RNA from the cells following the manufacturer's protocol. Then, the One Step TB Green® PrimeScript™ RT-PCR Kit II (Takara) was used to evaluate the mRNA levels of heme oxygenase 1 (*HMOX1*), glutathione peroxidase 2 (*GPX2*), NAD(P)H: quinone oxidoreductase 1 (*NQO1*), peroxiredoxin 1 (*PRDX1*), superoxide dismutase 1 (*SOD1*), catalase (*CAT*), glutathione reductase (*GSR*), glutamate-cysteine ligase catalytic subunit (*GCLC*), thioredoxin (*TXN*), and glyceraldehyde 3-phosphate dehydrogenase (*GAPDH*). The primer sequences were as follows: human *HMOX1* forward, 5′-tcctgctcaacatccagctc-3′; human *HMOX1* reverse, 5′-cacatggcataaagccctac-3′; human *GPX2* forward, 5′-ttgatatcagtccccttcactg-3′; human *GPX2* reverse, 5′-ctgccctttattggtctcttc-3′; human *NQO1* forward, 5′-agtcatctcattccactgttgg-3′; and human *NQO1* reverse, 5′-catctggtaaaggaggttttcc-3′, human *PRDX1* forward, 5′-gccaagtgattggtgcttc-3′, human *PRDX1* reverse, 5′-aatggtgcgcttcgggtc-3′, human *SOD1* forward, 5′-ctcactctcaggagaccattg-3′, human *SOD1* reverse, 5′-cacaagccaaacgacttccag-3′, human *CAT* forward, 5′-tgcggagattcaacactgc-3′, human *CAT* reverse, 5′-ggcaatgttctcacacagac-3′, human *GSR* forward, 5′-tatgtgagccgcctgaatg-3′, human *GSR* reverse, 5′-ctgacctctattgtgggcttg-3′, human *GCLC* forward, 5′-gaagtggatgtggacaccag-3′, human *GCLC* reverse, 5′-cttgtagtcaggatggtttgc-3′, human *TXN* forward, 5′-gtagttgacttctcagccacg-3′, human *TXN* reverse, 5′-ctgacagtcatccacatctacttc-3′, human *GAPDH* forward, 5′-tgacaacgaatttggctacag-3′; and human *GAPDH* reverse, 5′- agggtctctctcttcctcttg-3′. Three replicate wells were evaluated for each group (n = 3).

Evaluations of the ROS levels

The ROS assay was performed according to the manufacturer's protocol for the ROS Assay Kit Photo-oxidation Resistant DCFH-DA (DOJINDO, Kyushu, Japan), a redox-sensitive dye that fluoresces after being oxidized by ROS. NHEKs were seeded into 96-well plates at a density of 7 × 10^4 cells/well. After culturing overnight for 24 or 48 h at 37°C in a 5% CO_2 incubator, the samples of control, 2.0%, and 4.0% hydrolyzed corn starch were added. The cells were washed twice with Hank's

buffered salt solution (HBSS), and then the ROS detection fluorescent reagent was added for 30 min in a 37°C, 5% CO_2 incubator. Subsequently, the cells were washed twice with HBSS, then 200 μM H_2O_2 diluted in HBSS was added, and the cells were reacted for 1 h in a 37°C, 5% CO_2 incubator. The cells were then washed twice with HBSS, and a fluorescence microplate reader was used to measure the ROS levels. Three replicate wells were evaluated for each group (n = 3).

Western blotting

NHEKs were seeded and grown in six-well plates until they were subconfluent in a 37°C, 5% CO_2 incubator. Then, the samples of the control and hydrolyzed corn starch were added after culturing for a specified time. The cell suspension was collected for NRF2 detection by separately extracting the nuclear and cytoplasmic proteins according to the NE-PER™ Nuclear and Cytoplasmic Extraction Reagents (Thermo Fisher Scientific Inc.) protocol, followed by protein quantification with the bicinchoninic acid protein assay kit (Takara).

Equal concentrations of protein samples were diluted in Novex Tris-Glycine Sodium Dodecyl Sulfate Sample Buffer (Invitrogen Corp., Carlsbad, California, USA) and NuPAGE Sample Reducing Agent (Invitrogen) followed by heating at 85°C for 3 min to denature the proteins. Then, the protein samples were subjected to sodium dodecyl sulfate-polyacrylamide gel electrophoresis and transferred to polyvinylidene difluoride membranes (Bio-Rad Laboratories, Inc., California USA). The membrane was blocked with 5% skim milk in tris-buffered saline and polysorbate 20 (FUJIFILM Wako Pure Chemical Corp., Osaka, Japan) for 1 h at room temperature, and the corresponding primary antibodies: β-actin (Cell Signaling Technology, Inc., Danvers, MA, USA) at a 1:5000 dilution and NRF2 (Proteintech Group, Inc., Rosemont, IL, USA) at a 1:500 dilution were incubated overnight at 4°C. The next day, the polyvinylidene difluoride membranes were washed three times with tris-buffered saline and polysorbate 20 and incubated with the corresponding rabbit antibody (Abcam; 1:20,000 dilution for NRF2 and 1:100,000 dilution for β-actin) for 1 h at room temperature. The target protein bands were detected with a chemiluminescence method using the ECL select western blotting detection

reagent (Cytiva, Tokyo, Japan) and Amersham ImageQuant 800 (Cytiva). The target protein was quantified using ImageJ (National Institutes of Health, Bethesda, MD, USA), and each protein amount was normalized to that of β-actin. Three replicate wells were evaluated for each group (n = 3).

Immunostaining

NHEKs were seeded and grown in six-well plates until they were subconfluent in a 37°C, 5% CO_2 incubator. Then, the samples of the control and 4.0% hydrolyzed corn starch were added. After culturing for 2 h, the cell supernatant was removed, and then 1 mL/well of 4% (w/v) formaldehyde (Pierce) solution dissolved in PBS was added and incubated at room temperature for 15 min. Then, after washing three times with PBS, 1 mL/well of 0.5% Triton-X (Sigma Aldrich, St. Louis, Missouri, USA) solution dissolved in PBS was added followed by incubation at room temperature for 15 min. After washing three times with PBS again, 2 mL/well of 3% bovine serum albumin (FUJIFILM Wako Pure Chemical Corp.) solution dissolved in PBS was added for blocking at room temperature for 1 h. Then, the primary antibody (dilution concentration 1:250) was incubated overnight at 4°C. The next day, after washing three times with PBS, a secondary antibody (dilution concentration 1:300) was added and incubated at room temperature for 30 min. After washing three times with PBS, 1 mL of PBS was added to each well, followed by the addition of 2 drops/well of NucBlue™ Live ReadyProbes™ Reagent (Invitrogen). The plate was covered with aluminum foil to block light, incubated at room temperature for 20 min, and observed under a fluorescence microscope.

Statistical analysis

JMP® statistical analysis software (JMP Statistical Discovery LLC, Cary, NC, USA) was used to perform Student's *t*-test and Dunnett's test to determine the significance between the hydrolyzed corn starch-treated and control groups. Data are presented as the mean ± SD and "n" depicts the number of biological replicates in each experiment. Values of $p < 0.05$ were considered significant.

Results

Effect of hydrolyzed corn starch treatment on the gene-expression profiles

The statistical comparison of control cells and those treated with 2.0% hydrolyzed corn starch for 24 h according to the Rank Products method with multiple testing corrections using the Benjamini and Hochberg FDR ($p < 0.15$) resulted in the selection of 73 probes (S1 Table). Subsequently, we used the online Database for Annotation, Visualization, and Integrated Discovery software module to identify the overrepresented Keywords and Gene Ontology (GO) terms in the selected genes. A functional analysis of the selected 73 probes demonstrated that the effects of hydrolyzed corn starch treatment indicated significant involvement in the oxidation reduction (KW-0560, GO:0016491). In addition, 14 genes related to the oxidation reduction were found (Table 1). Among these genes, *NQO1*, Aldo-keto reductase family 1 member C1 (*AKR1C1*), Aldo-keto reductase family 1 member C2 (*AKR1C2*), Aldo-keto reductase family 1 member C3 (*AKR1C3*), Cytochrome P450 family 1 subfamily A member 1 (*CYP4F11*), *GPX2*, and *HMOX1* were upregulated more than 2-fold by treatment with hydrolyzed corn starch. We focused on *HMOX1*, *GPX2*, and *NQO1*, which are commonly regulated by NRF2. To confirm the reproducibility of the microarray data on upregulation of these genes by hydrolyzed corn starch treatment, the mRNA expressions of *HMOX1*, *GPX2*, and *NQO1* in keratinocytes were measured by reverse transcription quantitative polymerase chain reaction (RT-qPCR) (Fig 2A). The findings indicated a concentration-dependent increase in mRNA expressions when treated with 1.0%, 2.0%, and 4.0% hydrolyzed corn starch for 24 h.

Furthermore, we examined the effect of hydrolyzed corn starch at different exposure times (3, 6, 10, 24, and 48 h) on the mRNA expression of these enzymes in keratinocytes (Figs 2B-D). Hydrolyzed corn starch increased the mRNA expression of *HMOX1*, *GPX2*, and *NQO1* at different times in a gene dependent manner.

To further characterize the antioxidant response to hydrolyzed corn starch, additional antioxidant-

related gene expression analyses were performed. The mRNA expression levels of *PRDX1*, *SOD1*, *CAT*, *GSR*, *GCLC*, and *TXN* were determined by RT-qPCR after 24 h treatment with 1.0%, 2.0%, and 4.0% hydrolyzed corn starch (S1 Fig). As shown in S1 Fig, the mRNA expression levels of *GCLC* and *TXN* were upregulated in a concentration-dependent manner, and that of *PRDX1* was significantly increased at 4.0% hydrolyzed corn starch compared with control (*$p < 0.05$). In contrast, *SOD1*, *CAT*, and *GSR* showed no significant changes under these conditions.

Table 1. Oxidation reduction-related genes

UNIGENE	GENE NAME
Hs.406515	NAD(P)H quinone dehydrogenase 1 (*NQO1*)
Hs.460260	Aldo-keto reductase family 1 member C1 (*AKR1C1*)
Hs.460260, Hs.567256	Aldo-keto reductase family 1 member C2 (*AKR1C2*)
Hs.78183	Aldo-keto reductase family 1 member C3 (*AKR1C3*)
Hs.72912	Cytochrome P450 family 1 subfamily A member 1 (*CYP1A1*)
Hs.187393	Cytochrome P450 family 4 subfamily F member 11 (*CYP4F11*)
Hs.179608	Dehydrogenase/reductase 9 (*DHRS9*)
Hs.2704	Glutathione peroxidase 2 (*GPX2*)
Hs.517581	Heme oxygenase 1 (*HMOX1*)
Hs.596461	Isocitrate dehydrogenase (NADP(+)) 2 (*IDH2*)
Hs.464071	Phosphogluconate dehydrogenase (*PGD*)
Hs.495728	Pirin (*PIR*)
Hs.196384	Prostaglandin-endoperoxide synthase 2 (*PTGS2*)
Hs.654922	Thioredoxin reductase 1 (*TXNRD1*)

Fig 2. Effects of hydrolyzed corn starch on the mRNA expression levels of the oxidative stress-related genes in keratinocytes.

(A) Cells were treated with 1.0%, 2.0%, and 4.0% hydrolyzed corn starch for 24 h. (B-D) Cells were treated with 2.0% hydrolyzed corn starch for 3, 6, 10, 24, and 48 h. The mRNA levels of *HMOX1* (B), *GPX2* (C), and *NQO1* (D) were determined by RT-qPCR and normalized to that of *GAPDH*. Dunnett's test, *$p < 0.05$, **$p < 0.01$, ***$p < 0.001$ (n = 3).

Effects of hydrolyzed corn starch on the ROS level in keratinocytes exposed to hydrogen peroxide (H_2O_2)

To evaluate the antioxidant response to treatment with 2.0% and 4.0% hydrolyzed corn starch for 24 and 48 h, intracellular ROS levels were measured using the fluorescent probe DCFH-DA (Figs 3A and 3B). As shown in Figs 3A and 3B, compared with the H_2O_2-untreated group, the intracellular ROS production was increased in the H_2O_2-treated group. Pretreatment with the hydrolyzed corn starch inhibited the increase in ROS production induced by H_2O_2-treatment, and 4.0% hydrolyzed corn starch after 48 h exposure significantly inhibited ROS production relative to that of the control ($p < 0.001$) (Fig 3B). Typical fluorescence images of the cells are shown in Fig 3C. The fluorescence caused by the oxidation of DCFH-DA was increased by H_2O_2, and the change was reduced by hydrolyzed corn starch.

Fig 3. Effects of hydrolyzed corn starch on the reactive oxygen species (ROS) production in keratinocytes exposed to H_2O_2.

(A, B) Cells were labeled with DCFH-DA, treated with 2.0% and 4.0% hydrolyzed corn starch for 24 h (A) and 48 h (B), and exposed to 200 μM H_2O_2 for 1 h. The fluorescence intensity of the cells was measured to determine the ROS levels. (C) Typical images of cells fluorescing due to the oxidation of DCFH-DA by ROS. Dunnett's test, ***$p < 0.001$ (n = 3).

Effects of hydrolyzed corn starch on NRF2 activation

The activation of NRF2 occurs by translocating NRF2 into the nucleus from the cytosol. After NRF2 translocates into the nucleus, it can bind to the ARE and drive the expression of target genes,

such as *HMOX1*, *GPX2*, and *NQO1*.

To analyze the protein level of NRF2 in the nucleus, we performed western blotting on cells treated with 4.0% hydrolyzed corn starch for 2 h. β-actin served as a reference protein for normalization. As shown in Fig 4A, 4.0% hydrolyzed corn starch increased the protein levels of NRF2 in the nucleus, and 4.0% hydrolyzed corn starch significantly increased NRF2 in the nucleus compared with that in the control ($p < 0.05$). Furthermore, Fig 4B shows that NRF2 is localized from the cytosol to the nucleus in the cells treated with 4.0% hydrolyzed corn starch for 2 h.

Fig 4. Effects of hydrolyzed corn starch on NRF2 activation in keratinocytes.

(A) Cells were treated with 4.0% hydrolyzed corn starch for 2 h, and nuclear NRF2 protein levels were determined by western blotting and normalized to that of nuclear β-actin. Student's *t*-test, $*p < 0.05$ (n = 3). (B) Nuclear translocation of NRF2 was determined by immunostaining.

Discussion

The skin generates ROS in response to external stimuli, such as ultraviolet radiation and air pollution. The transcription factor NRF2 plays a central role in the cellular defense mechanism against oxidative stress. Upon translocation from the cytoplasm to the nucleus, NRF2 binds to the ARE and upregulates genes, including *HMOX1*, *GPX2*, and *NQO1*. The *HMOX1* gene encodes heme oxygenase-1, which is a key enzyme that catalyzes the rate-limiting first step of heme degradation, yielding carbon monoxide, ferrous, and biliverdin. Biliverdin is rapidly reduced to bilirubin, a known strong antioxidant [14]. *GPX2* reduces hydrogen peroxide and lipid hydroperoxides at the expense of glutathione, whereas *NQO1* reduces quinone to hydroquinone. These antioxidant enzymes act both directly and indirectly to help alleviate oxidative stress in the long term [15]. Conversely, when ROS levels become excessively high, oxidative damage to DNA and proteins can occur along with increased synthesis and activation of matrix metalloproteinases, enzymes that degrade extracellular matrix components, and higher ROS levels are thought to promote collagen and elastin degradation, leading to skin aging, such as wrinkles and sagging [16, 17]. Furthermore, ROS stimulate keratinocytes and

release various melanocyte activating factors, such as endothelin-1 and protease-activated receptor-2, which is known to promote melanin synthesis and melanosome transport [18, 19], and is thought to cause skin problems, such as age spots and pigmentation.

In this study, we demonstrated that hydrolyzed corn starch activates the NRF2 pathway in keratinocytes, leading to increase the expression of antioxidant-responsive genes and a subsequent reduction in ROS levels. Specifically, NRF2 activation occurred 2 h after the application of hydrolyzed corn starch, subsequently leading to ≥2-fold changes in *NQO1* at 6 h, *HMOX1* at 10 h, and *GPX2* at 48 h and ultimately leading to the inhibition of H_2O_2-dependent ROS generation at 48 h. These findings indicate that hydrolyzed corn starch enhances the cellular defense capacity against oxidative stress via the NRF2 pathway in keratinocytes.

In addition, previous studies have suggested that the expressions of *GCLC*, *TXN*, *PRDX1*, *SOD1*, *CAT*, and *GSR* can be regulated by NRF2 [2, 20, 21], although the extent of NRF2 involvement may vary depending on the cell type and experimental conditions. In our study, treatment with 1.0%, 2.0%, and 4.0% hydrolyzed corn starch for 24 h upregulated the mRNA expression levels of *GCLC* and *TXN* in a concentration-dependent manner and significantly increased that of *PRDX1* at 4.0%, whereas *SOD1*, *CAT*, and *GSR* showed no significant changes under these conditions. Taken together with the observed induction of *HMOX1*, *GPX2*, and *NQO1,* these additional changes in antioxidant-related genes further support the possibility that the overall gene-expression profile induced by hydrolyzed corn starch is, at least in part, a consequence of NRF2 activation, although contributions from other transcriptional regulators cannot be excluded.

Given that excessive ROS are known to contribute to collagen and elastin degradation and to hyperpigmentation through the activation of melanocyte-stimulation factors [16-19], NRF2 activation and ROS reduction by hydrolyzed corn starch raise the possibility that this ingredient may indirectly influence skin conditions such as wrinkles, sagging, and pigmentation. However, our current data are limited to antioxidant responses in keratinocytes, and we did not directly assess structural or functional

endpoints (e.g., collagen content, skin elasticity, melanin production) in the skin. Therefore, any anti-wrinkle, anti-sagging, or depigmenting effects remain speculative and should be validated in future *in vitro* and clinical studies. In addition, our experiments were conducted exclusively in keratinocytes and did not address the role of dermal fibroblasts, which are crucial for extracellular matrix production and skin elasticity. Future investigations using dermal fibroblasts, melanocyte-keratinocyte co-culture systems, and *in vivo* or clinical evaluations will be required to clarify whether the NRF2 activation and ROS reduction observed here translate into measurable improvements in these skin conditions.

People with sensitive skin often have low-barrier skin function, making them susceptible to external stimuli and increased oxidative stress. The multifunctional profile of hydrolyzed corn starch, including moisturizing, itch-relief, and antioxidant effects via NRF2 activation, supports its potential application as a cosmetic ingredient for sensitive skin.

Moreover, recent studies have demonstrated that activation of the NRF2 pathway can suppress inflammation by inhibiting the expression of interleukin 6 and interleukin 1β, cytokines that exacerbate inflammation [16]. On the basis of these reports, the NRF2 activation observed in our study raises the possibility that hydrolyzed corn starch may have anti-inflammatory effects on the skin. However, we did not measure inflammatory markers, cytokine expression, or other indices of inflammation in our experiments. Thus, any anti-inflammatory effect of hydrolyzed corn starch should be regarded as a hypothesis derived from the known function of NRF2 rather than as an observed effect.

Our results also suggest that maltotetraose (MTO), a major component of hydrolyzed corn starch, may play a key role in NRF2 activation and subsequent ROS reduction in keratinocytes, although the detailed mechanism remains unclear. Whether ROS reduction is solely mediated by the NRF2 pathway and the specific mechanism by which MTO activates NRF2 are not yet established. In addition, we did not evaluate the overall status of enzymatic and non-enzymatic antioxidant capacity in this study, and thus the global impact of MTO on the cutaneous antioxidant defense system remains unclear. A comprehensive understanding will require the identification of the individual compounds in the

hydrolyzed corn starch, subsequent validation using each individual compound, and elucidation of the associated molecular interactions and signaling pathways with specific inhibitors.

Conclusion

The main focus of this study was to evaluate the potential novel beneficial effects of hydrolyzed corn starch containing maltotetraose on the skin, particularly defense against oxidative stress. The results suggest that hydrolyzed corn starch enhances cellular antioxidant capacity through induction of antioxidant-responsive genes, thereby contributing to the reduction of ROS levels. Furthermore, the results also suggest that hydrolyzed corn starch enhances cellular defense against oxidative stress by NRF2 activation.

Acknowledgments

We thank Dr. Tetsuji Hirao (Professor, School of Pharmacy and Pharmaceutical Sciences, Mukogawa Women’s University), for valuable technical advice and support throughout this study.

Data Availability

All microarray data files have been deposited in the NCBI Gene Expression Omnibus (GEO) under accession number GSEXXXXXX (to be updated once the accession number is assigned). All other datasets used for the statistical analyses, including individual data points and sample sizes for each experiment, have been deposited in the Zenodo repository and are freely available under the accession DOI 10.5281/zenodo.19227766. All other relevant data are within the manuscript and its Supporting Information files.

References

1. Jaffri JM. Reactive oxygen species and antioxidant system in selected skin disorders. Malays J Med Sci. 2023;30: 7
2. Ngo V, Duennwald ML. Nrf2 and oxidative stress: A general overview of mechanisms and implications in human disease. Antioxidants. 2022;11: 2345
3. Frants MC, Rozot R, Marrot L. NRF2 in dermo-cosmetic: From scientific knowledge to skin care products. BioFactors. 2023;49: 32-61.
4. Chen J, Liu Y, Zhao Z, Qiu J. Oxidative stress in the skin: Impact and related protection. Int J Cosmet Sci. 2021;43: 495-509
5. Schäfer M, Werner S. Nrf2-A regulator of keratinocyte redox signaling. Free Radical Biology and Medicine. 2015;88: 243-252
6. Salman S, Paulet V, Hardonniére K, Kerdine-Römer S. The role of NRF2 transcription factor in inflammatory skin diseases. Biofactors. 2025;51(2)
7. Kim HJ, Hong JH. Emerging Therapeutic Strategies for Nrf2-Associated Skin Disorders: From Photoaging to Autoimmunity. Antioxidants. 2026;15(1): 69
8. Andersen FA, Bergfeld WF, Belsito DV, Klaassen CD, Marks JG Jr, Shank RC, et al. Final Report of the Safety Assessment of Cosmetic Ingredients Derived From Zea Mays (Corn). International Journal of Toxicology. 2011;30(Supplement 1): 17S-39S
9. Jiang C, Guo C, Yan J, Chen J, Peng S, Huang H, et al. Sensitive skin syndrome: Research progress on mechanisms and applications. J Dermatol Sci Cosmet Technol. 2024;1: 100015.
10. Ständer S, Yosipovitch G. Substance P and neurokinin 1 receptor are new targets for the treatment of chronic pruritus. Br J Dermatol. 2019;81: 932-938.
11. Alam M, Buddenkotte J, Ahmad F, Steinhoff M. Neurokinin 1 receptor antagonists for pruritus. Drugs. 2021;81: 621-634.
12. Uozumi T, Morishita S, Tanaka Y, Yumoto M. NK1 receptor antagonist composition. US Patent

8426386 B2, April 23, 2013.
13. Ichikawa E, Inoue A, Matsuzaki K, Kaneda S, Nito A, Yokoyama M. Clinical evaluation of the efficacy of itch-relief moisturizers containing maltotetraose for dry, itchy, and sensitive skin. Skinmed. 2024;22: 187-196.
14. Stocker R, Yamamoto Y, McDonagh AF, Glazer AN, Ames BN. Bilirubin is an antioxidant of possible physiological importance. Science. 1987;235: 1043-1046.
15. Kensler TW, Wakabayashi N, Biswal S. Cell survival responses to environmental stresses via the Keap1-Nrf2-ARE pathway. Annu Rev Pharmacol Toxicol. 2007;47: 89-116
16. Fisher GJ, Datta SC, Talwar HS, Wang ZQ, Varani J, Kang S, et al. Molecular basis of sun-induced premature skin ageing and retinoid antagonism. Nature. 1996;379: 335-339.
17. Fisher GJ, Talwar HS, Lin J, Lin P, McPhillips F, Wang Z, et al. Retinoic acid inhibits induction of c-Jun protein by ultraviolet radiation that occurs subsequent to activation of mitogen-activated protein kinase pathways in human skin in vivo. J Clin Invest. 1998;101: 1432-1440.
18. Imokawa G, Yada Y, Miyagishi M. Endothelins secreted from human keratinocytes are intrinsic mitogens for human melanocytes. J Biol Chem. 1992;267: 24675-24680.
19. Li J, Tang LY, Fu WW, Yuan J, Sheng YY, Yang QP. Low-concentration hydrogen peroxide can upregulate keratinocyte intracellular calcium and PAR-2 expression in a human keratinocyte–melanocyte co-culture system. Arch Dermatol Res. 2016;308: 723-731.
20. He F, Ru X, Wen T. NRF2, a Transcription Factor for Stress Response and Beyond. Int J Mol Sci. 2020;21(13): 4777
21. Jung K, Kwak Mi. The Nrf2 System as a Potential Target for the Development of Indirect Antioxidants. Molecules. 2010;15(10): 7266-7291
22. Kobayashi EH, Suzuki T, Funayama R, Nagashima T, Hayashi M, Sekine H, et al. Nrf2 suppresses macrophage inflammatory response by blocking proinflammatory cytokine transcription. Nat Commun. 2016;7: 11624.

Supporting information

S1 Table. 73 differentially expressed probes. Cells were treated with 2.0% hydrolyzed corn starch for 24 h. Differentially expressed probes were identified by microarray analysis.

S1 Fig. Effects of hydrolyzed corn starch on the mRNA expression levels of antioxidant related genes. Cells were treated with 1.0%, 2.0%, and 4.0% hydrolyzed corn starch for 24 h.

Figure

Click here to access/download;Figure;Fig1.tif

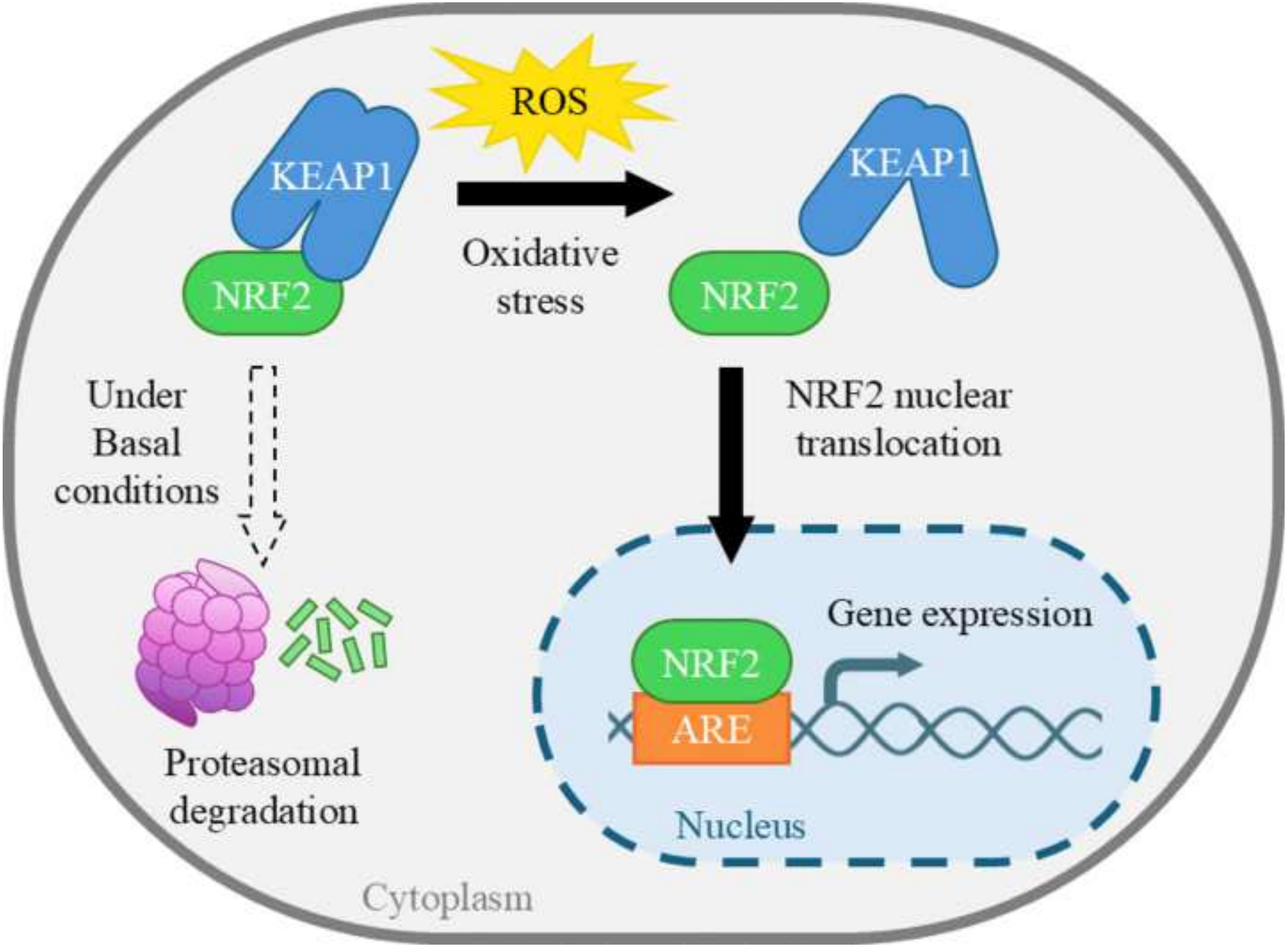

Figure

Click here to access/download;Figure;Fig2.tif

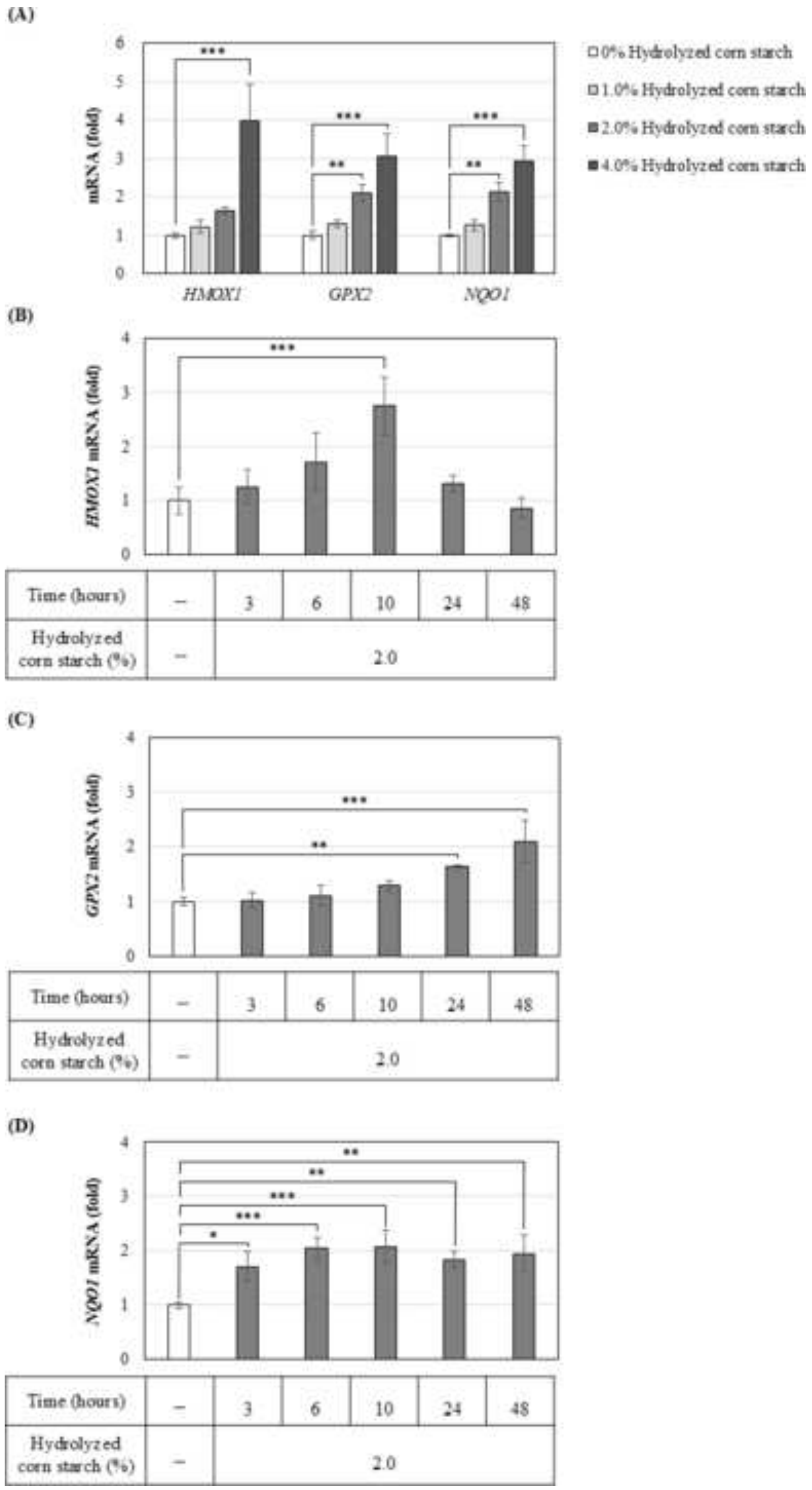

Figure
Click here to access/download;Figure;Fig3.tif

(A)

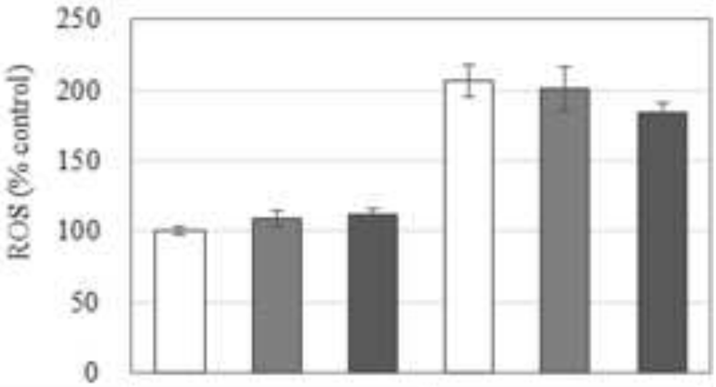

H_2O_2 (μM)	–			200		
Hydrolyzed corn starch (%)	–	2.0	4.0	–	2.0	4.0
ROS (%)	100	108	112	206	200	183

(B)

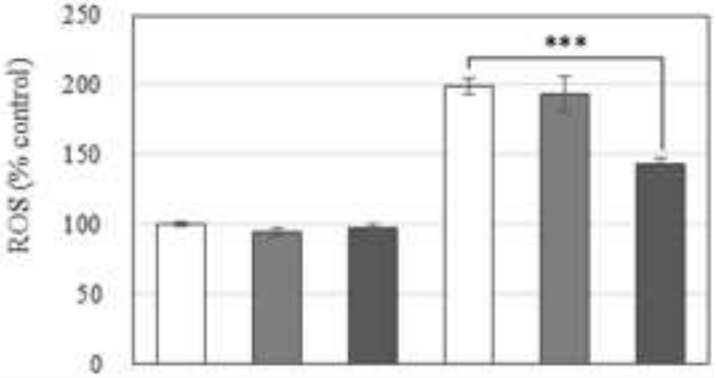

H_2O_2 (μM)	–			200		
Hydrolyzed corn starch (%)	–	2.0	4.0	–	2.0	4.0
ROS (%)	100	95	97	199	194	143

(C)

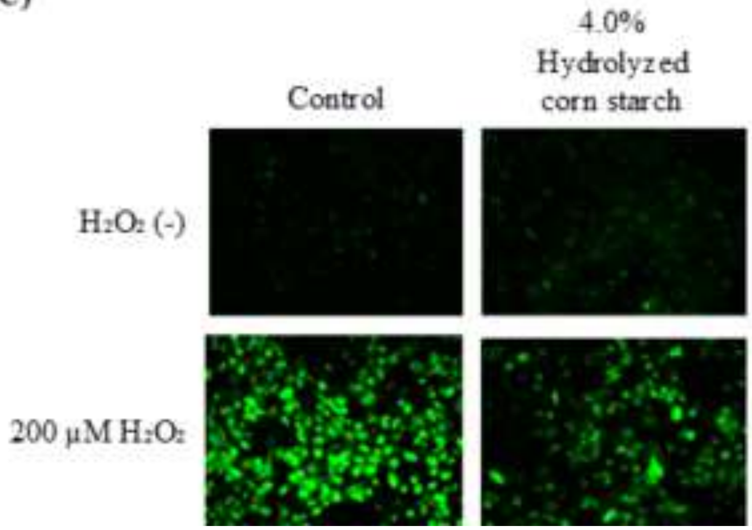

Figure

Click here to access/download;Figure;Fig4.tif

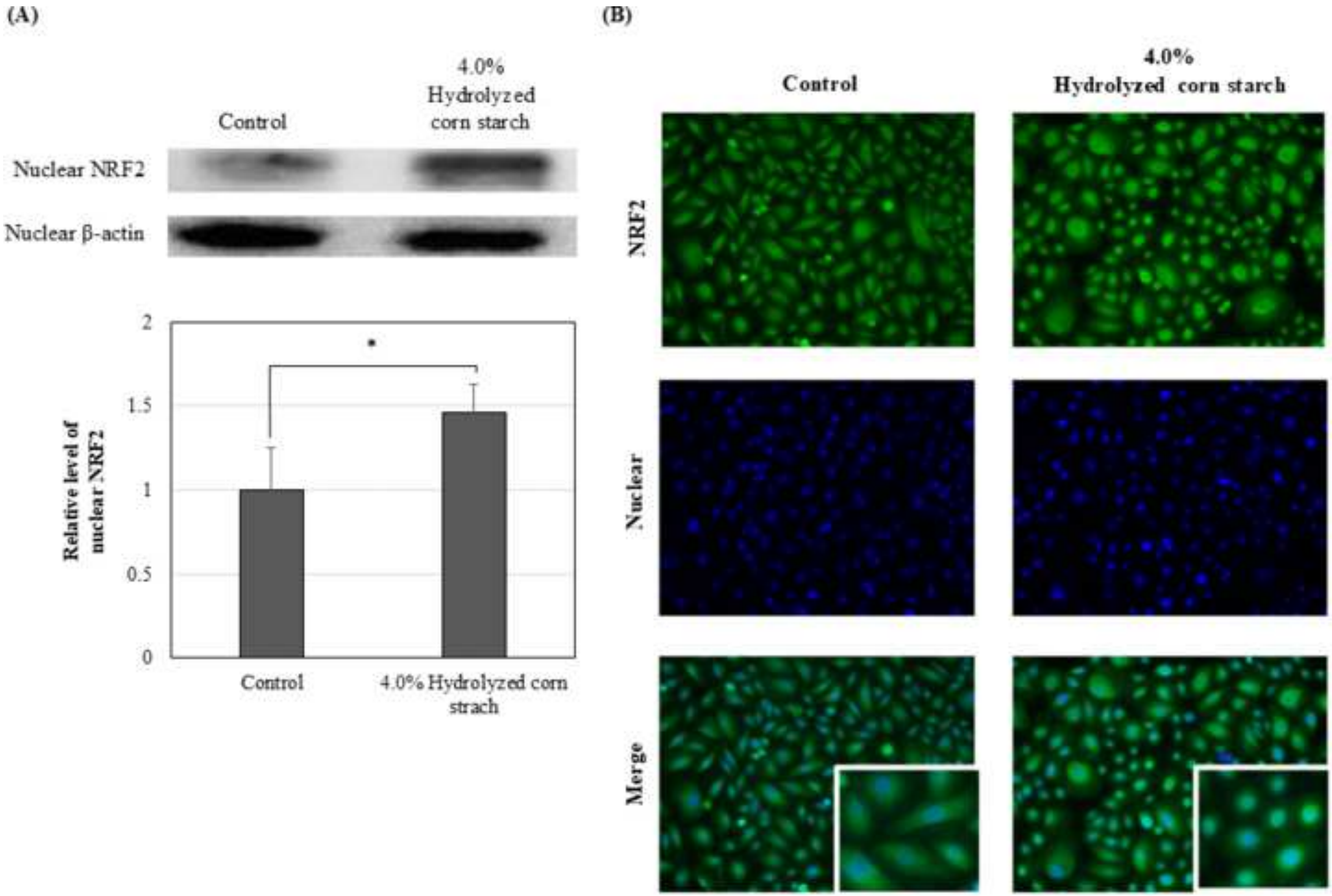

Click here to access/download
Supporting Information
S1_Table.docx

Click here to access/download
Supporting Information
S1_raw_images.pdf

Click here to access/download
Supporting Information
S1_Fig.tif

Hydrolyzed corn starch with maltotetraose for skin defense through NRF2 pathway activation in human keratinocytes

Short title: Hydrolyzed corn starch with maltotetraose induced antioxidative responses via NRF2 pathway

Hitomi Hagawa[1*], Eri Ichikawa[2], Keiko Otake[1], Mayuko Ishii[1], Naoko Kawaguchi[1]

[1] Global Development Center, Research & Development Headquarters, Lion Corporation, Edogawa-ku, Tokyo, Japan

[2] Northeast Asia Business Division, Business Development Department 1, Lion Corporation, Tokyo, Japan

* Corresponding author:

E-mail: hagawa-h@lion.co.jp (HH)

Abstract

Oxidative stress, which can be triggered by various external stimuli, such as ultraviolet radiation and pollution, compromises skin health, accelerates aging and skin disorders, and impacts quality of life. This study investigated the potential of hydrolyzed corn starch containing maltotetraose in skin defense through nuclear factor erythroid 2-related factor 2 (NRF2) pathway activation in human keratinocytes exposed to oxidative stress. Using DNA microarray analysis and assessing key antioxidant-responsive genes, we evaluated the capacity of this ingredient~~hydrolyzed corn starch~~ to enhance cellular defense mechanisms. Human keratinocytes treated with it ~~hydrolyzed corn starch~~ demonstrated significant upregulation of antioxidant-responsive genes, including *HMOX1*, *GPX2*, and *NQO1*, which are known targets of NRF2. These findings were corroborated by western blotting and immunostaining analyses, which confirmed increased NRF2 protein expression and NRF2 nuclear translocation. Moreover, fluorescence assays and microscopy showed that treatment with the ingredient~~hydrolyzed corn starch~~ effectively reduced reactive oxygen species levels in keratinocytes exposed to oxidative stress. These results suggest~~ed~~ that ~~hydrolyzed corn starch containing maltotetraose~~ activation~~es~~ of the NRF2 pathway by this ingredient~~, thereby,~~ enhances~~ing~~ the cellular antioxidative response and reduces~~ing~~ reactive oxygen species levels in keratinocytes. This ~~novel functionality~~NRF2-mediated antioxidative activity positions hydrolyzed corn starch containing maltotetraose ~~maltotetoraose~~ as a promising ingredient for cosmetic products, with potential implications for improving skin health, reducing pigmentation, and combating aging. However, these effects were not directly evaluated in this study. Future studies are warranted to evaluate its efficacy *in vivo*.

Introduction

The skin serves as the interface between the external and internal environments and is continuously exposed to various external stimuli, including ultraviolet radiation, air pollution, tobacco smoke, and alcohol [1-3]; these stimuli generate reactive oxygen species (ROS). Excessive or prolonged exposure to ROS leads to oxidative stress [4], which in turn exacerbates skin pigmentation and aging, ultimately resulting in uneven skin tone, hyperpigmentation, roughness, sagging, and wrinkles. This may lead to cosmetic imperfections and progress to severe dryness, sensitivity, and more severe conditions, such as atopic dermatitis, psoriasis, and skin cancer, thereby significantly impacting quality of life. Considering that the skin is continuously subjected to oxidative stress, potent antioxidant defenses are essential, especially for low-barrier or sensitive skin, which is particularly susceptible to external stimuli.

The skin possesses a robust antioxidant defense system, with the transcription factor nuclear factor erythroid 2-related factor 2 (NRF2) playing a central role by regulating the expression of a wide array of antioxidant enzymes responsible for detoxifying and eliminating oxidative agents. ~~In the absence of oxidative stress,~~ Under basal conditions, NRF2 is maintained in an inactive state in the cytoplasm through its binding to Kelch-like ECH-associated protein 1 (Keap1) and is directed toward ~~protein~~ proteasomal degradation. Under oxidative stress, NRF2 is released from Keap1~~, becomes phosphorylated,~~ and translocates into the nucleus, where it binds to the antioxidant ~~responsive~~response element (ARE). This interaction modulates key skin defense mechanisms, including phase II detoxification, inflammatory signaling, DNA repair, and antioxidant responses (Fig 1) [5, 6]. In the skin, the NRF2 pathway has been implicated in photoprotection, regulation of pigmentation, wound healing, and reinforcement of the epidermal barrier function [3, 7]. From a cosmetic perspective, enhancement of NRF2 signaling is therefore expected to strengthen endogenous antioxidant and barrier defenses, thereby helping to prevent photoaging, pigmentation disorders, and barrier impairment induced by environmental stimuli. Moreover, transient NRF2 activation is unlikely

to be harmful, as NRF2 upregulates Keap1 expression via a negative feedback mechanism that contributes to the maintenance of physiological redox balance in cells [3]. Consequently, incorporating NRF2 modulators into cosmetic formulations can be viewed as a promising strategy to protect the skin from environmental stress and to maintain skin homeostasis.

~~Given its role in modulating various physiological processes in the skin, NRF2 represents a promising target in cosmetic applications aimed at protection, care, and repair.~~

Hydrolyzed corn starch is officially categorized as a skin conditioning agent, specifically a humectant, in cosmetic ingredient nomenclature [8]. We have identified hydrolyzed corn starch, derived from corn, as a safe and effective ~~raw and~~ naturally derived functional cosmetic ingredient. This starch mainly contains maltotetraose (MTO), a polymer of glucose linked by α-1,4-glycosidic bond. Given its higher concentration of hydroxyl groups compared to other humectants, such as glycerin and butylene glycol, MTO~~ exhibits superior skin-moisturizing properties~~ has potentially enhanced moisturizing capacity. Moisturization is a fundamental aspect of skin care; for low-barrier and sensitive skin, application of moisturizers is crucial for restoring the skin barrier [~~5~~9]. In addition, the itch–scratch cycle, observed in sensitive skin, can significantly impair quality of life. Our previous studies have demonstrated that MTO possesses notable itch-suppressing effects. Specifically, MTO significantly suppresses chronic itching by modulating mechanisms involving substance P and the neurokinin 1 receptor (NK1R) [~~6, 7~~10, 11], notably through inhibition of NK1R activation [~~8~~12]. Based on these findings, we developed an itch-relief moisturizer containing MTO. A clinical trial involving patients with dry, itchy, and sensitive skin demonstrated that treatment with MTO effectively moisturized the skin, enhanced the skin barrier, and reduced itching [~~9~~13]. Therefore, MTO holds promise as a functional component in the cosmetic field.

In this study, we aimed to investigate the other potential applications of MTO as a cosmetic ingredient by exploring its novel functionalities using human epidermal skin cells. Specifically, we examined the possibility of enhancing the skin's ability to defend against oxidative stress by activating

NRF2 and reducing ROS.

Fig 1. NRF2-Keap1 pathway under basal and oxidative stress conditions.

Under basal conditions, NRF2 is retained in the cytoplasm through its interaction with Keap1, which promotes NRF2 ubiquitination and proteasomal degradation. In the presence of oxidative stress, structural changes in Keap1 lead to the release and stabilization of NRF2. The stabilized NRF2 translocates into the nucleus, where it binds to ARE in the promoters of target genes. This induces the expression of phase II detoxification enzymes, antioxidant enzymes, and other cytoprotective molecules.

Formatted: Indent: First line: 0"

Formatted: Font:

Materials and methods

Ethics Statement

Normal Human Epidermal Keratinocytes (NHEK; adult, single donor) were purchased from PromoCell GmbH (Heidelberg, Germany; Cat. No. C-12003; Lot Nos. 489Z018.2, 470Z025.1, 479Z032.1). PromoCell obtains donor tissues only after written informed consent and in accordance with the Declaration of Helsinki, providing fully anonymized samples (https://www.promocell.com/ethics). Under Lion Corporation Institutional Review Board (IRB) policy, research readily obtainable from commercial sources is exempt from additional review.

Cell culture

Human keratinocytes (NHEKs; PromoCell, Cat. No. C-12003) ~~cells~~ were purchased from Takara Bio Inc. (Shiga, Japan; Cat. No. C-12003) and cultured in Keratinocyte Growth Medium 2 (PromoCell, Cat. No. C-20011~~; Takara~~) containing 5 µg/mL insulin, 0.125 ng/mL human recombinant epidermal growth factor, 0.33 µg/mL hydrocortisone, 0.39 µg/mL epinephrine, 10 µg/mL holo-transferrin, 0.06 mM $CaCl_2$ and 0.004 ml/mL bovine pituitary extract at 37°C in a humidified atmosphere of 5% CO_2. Hydrolyzed corn starch (Leogard MT; Lion Specialty Chemicals Co., Ltd., Tokyo, Japan) was added to dissolve in the medium with NHEKs.

DNA microarray analysis

NHEKs were seeded and grown in six-well plates until they were subconfluent in a 37℃, 5% CO_2 incubator. Then, the control samples (only the medium) and 2.0% hydrolyzed corn starch in the medium were added, and after culture for 24 h, RNeasy Plus Mini Kit (Qiagen, Hilden, Germany) was used to extract RNA from the cells following the manufacturer's protocol. The quality and quantity of the total RNA were evaluated on an Agilent 2100 Bioanalyzer (Agilent Technologies Japan, Ltd., Tokyo, Japan) and the total RNA integrity number exceeded seven. These total RNA samples were subjected to DNA microarray analysis using a Clariom™ S Array, human (Thermo Fisher Scientific Inc., MA, USA) by Kurabo (Osaka, Japan), and the fluorescence signals were scanned with a Biosystems™ GeneChip Scanner 3000 7G System. Biosystems™ GeneChip Command Console Software ver. 3.2 was used to reduce the array images to the intensity values for each probe (CEL files).

DNA microarray data analysis

Using R software (ver. 4.3.2; The R Project, https://www.r-project.org/), the CEL files obtained from three independent samples per condition (n=3) were quantified according to the distribution Robust Multichip Analysis. A statistical comparison of the control and 2.0% hydrolyzed corn starch was performed with the Rank Products and Benjamini and Hochberg false discovery rate (FDR) corrections. The Human Genome Array annotation file was used (Clariom™ S Array, human Transcript Cluster Annotations, CSV, Release 36). Differentially expressed genes were extracted on setting the values of $p < 0.15$, and the Database for Annotation, Visualization, and Integrated Discovery ~~was~~were used to classify the selected probe sets according to functional annotations. The functional analysis was performed on the basis of the molecular function in UniProtKB(UP)_Keywords(KW)_MF. In addition, while comparing cells treated with 2.0% hydrolyzed corn starch and control cells, genes that exhibited signal intensities of 20% to 100% with

a fold change of ≥2.0 were considered upregulated. A significant difference was defined as an FDR-corrected value of $p < 0.05$.

Gene-expression analysis

NHEKs were seeded and grown in six-well plates until they were subconfluent in a 37℃, 5% CO_2 incubator. Then, the samples of control and 1.0%, 2.0% and 4.0% hydrolyzed corn starch were added, and after culturing for 3, 6, 10, 24, and 48 h, an RNeasy Plus Mini Kit (Qiagen) was used to extract RNA from the cells following the manufacturer's protocol. Then, the One Step TB Green® PrimeScript™ RT-PCR Kit II (Takara) was used to evaluate the mRNA levels of heme oxygenase 1 (*HMOX1*)~~1~~, glutathione peroxidase 2 (*GPX2*)~~2, and~~ NAD(P)H: quinone oxidoreductase 1 (*NQO1*)~~1~~, peroxiredoxin 1 (*PRDX1*), superoxide dismutase 1 (*SOD1*), catalase (*CAT*), glutathione reductase (*GSR*), glutamate-cysteine ligase catalytic subunit (*GCLC*), thioredoxin (*TXN*), and glyceraldehyde 3-phosphate dehydrogenase (*GAPDH*). The primer sequences were as follows: human *HMOX1* forward, 5′-tcctgctcaacatccagctc-3′; human *HMOX1* reverse, 5′-cacatggcataaagccctac-3′; human *GPX2* forward, 5′-ttgatatcagtccccttcactg-3′; human *GPX2* reverse, 5′-ctgccctttattggtctcttc-3′; human *NQO1* forward, 5′-agtcatctcattccactgttgg-3′; and human *NQO1* reverse, 5′-catctggtaaaggaggttttcc-3′~~.~~, human *PRDX1* forward, 5′-gccaagtgattggtgcttc-3′, human *PRDX1* reverse, 5′-aatggtgcgcttcgggtc-3′, human *SOD1* forward, 5′-ctcactctcaggagaccattg-3′, human *SOD1* reverse, 5′-cacaagccaaacgacttccag-3′, human *CAT* forward, 5′-tgcggagattcaacactgc-3′, human *CAT* reverse, 5′-ggcaatgttctcacacagac-3′, human *GSR* forward, 5′-tatgtgagccgcctgaatg-3′, human *GSR* reverse, 5′-ctgacctctattgtgggcttg-3′, human *GCLC* forward, 5′-gaagtggatgtggacaccag-3′, human *GCLC* reverse, 5′-cttgtagtcaggatggtttgc-3′, human *TXN* forward, 5′-gtagttgacttctcagccacg-3′, human *TXN* reverse, 5′-ctgacagtcatccacatctacttc-3′, human *GAPDH* forward, 5′-tgacaacgaatttggctacag-3′; and human *GAPDH* reverse, 5′- agggtctctctcttcctcttg-3′. Three replicate wells were evaluated for each group (n = 3).

Formatted: Font: Italic
Formatted: Font: Italic
Formatted: Font: Italic
Formatted: Font: Italic
Formatted: Font: Italic
Formatted: Font: Italic
Formatted: Font: Italic
Formatted: Font: Italic
Formatted: Font: Italic
Formatted: Font: Italic

Evaluations of the ROS levels

The ROS assay was performed according to the manufacturer's protocol for the ROS Assay Kit Photo-oxidation Resistant DCFH-DA (DOJINDO, Kyushu, Japan), a redox-sensitive dye that fluoresces after being oxidized by ROS. NHEKs were seeded into 96-well plates at a density of 7×10^4 cells/well. After culturing overnight for 24 or 48 h at 37°C in a 5% CO_2 incubator, the samples of control, 2.0%, and 4.0% hydrolyzed corn starch were added. The cells were washed twice with Hank's buffered salt solution (HBSS), and then the ROS detection fluorescent reagent was added for 30 min in a 37°C, 5% CO_2 incubator. Subsequently, the cells were washed twice with HBSS, then 200 μM H_2O_2 diluted in HBSS was added, and the cells were reacted for 1 h in a 37°C, 5% CO_2 incubator. The cells were then washed twice with HBSS, and a fluorescence microplate reader was used to measure the ROS levels. Three replicate wells were evaluated for each group (n = 3).

Western blotting

NHEKs were seeded and grown in six-well plates until they were subconfluent in a 37°C, 5% CO_2 incubator. Then, the samples of the control and hydrolyzed corn starch were added after culturing for a specified time. The cell suspension was collected for NRF2 detection by separately extracting the nuclear and cytoplasmic proteins according to the NE-PER™ Nuclear and Cytoplasmic Extraction Reagents (Thermo Fisher Scientific Inc.) protocol, followed by protein quantification with the bicinchoninic acid protein assay kit (Takara).

Equal concentrations of protein samples were diluted in Novex Tris-Glycine Sodium Dodecyl Sulfate Sample Buffer (Invitrogen Corp., Carlsbad, California, USA) and NuPAGE Sample Reducing Agent (Invitrogen) followed by heating at 85°C for 3 min to denature the proteins. Then, the protein samples were subjected to sodium dodecyl sulfate-polyacrylamide gel electrophoresis and transferred to polyvinylidene difluoride membranes (Bio-Rad Laboratories, Inc., California USA). The membrane was blocked with 5% skim milk in tris-buffered saline and polysorbate 20 (FUJIFILM Wako Pure Chemical Corp., Osaka, Japan) for 1 h at room temperature, and the corresponding primary antibodies: β-actin (Cell Signaling Technology, Inc., Danvers, MA, USA) at a 1:5000 dilution and NRF2

(Proteintech Group, Inc., Rosemont, IL, USA) at a 1:500 dilution were incubated overnight at 4°C. The next day, the polyvinylidene difluoride membranes were washed three times with tris-buffered saline and polysorbate 20 and incubated with the corresponding rabbit antibody (Abcam; 1:20,000 dilution for NRF2 and 1:100,000 dilution for β-actin) for 1 h at room temperature. The target protein bands were detected with a chemiluminescence method using the ECL select western blotting detection reagent (Cytiva, Tokyo, Japan) and Amersham ImageQuant 800 (Cytiva). The target protein was quantified using ImageJ (National Institutes of Health, Bethesda, MD, USA), and each protein amount was normalized to that of β-actin. Three replicate wells were evaluated for each group (n = 3).

Immunostaining

NHEKs were seeded and grown in six-well plates until they were subconfluent in a 37℃, 5% CO_2 incubator. Then, the samples of the control and 4.0% hydrolyzed corn starch were added. After culturing for 2 h, the cell supernatant was removed, and then 1 mL/well of 4% (w/v) formaldehyde (Pierce) solution dissolved in PBS was added and incubated at room temperature for 15 min. Then, after washing three times with PBS, 1 mL/well of 0.5% Triton-X (Sigma Aldrich, St. Louis, Missouri, USA) solution dissolved in PBS was added followed by incubation at room temperature for 15 min. After washing three times with PBS again, 2 mL/well of 3% bovine serum albumin (FUJIFILM Wako Pure Chemical Corp.) solution dissolved in PBS was added for blocking at room temperature for 1 h. Then, the primary antibody (dilution concentration 1:250) was incubated overnight at 4°C. The next day, after washing three times with PBS, a secondary antibody (dilution concentration 1:300) was added and incubated at room temperature for 30 min. After washing three times with PBS, 1 mL of PBS was added to each well, followed by the addition of 2 drops/well of NucBlue™ Live ReadyProbes™ Reagent (Invitrogen). The plate was covered with aluminum foil to block light, incubated at room temperature for 20 min, and observed under a fluorescence microscope.

Statistical analysis

JMP® statistical analysis software (JMP Statistical Discovery LLC, Cary, NC, USA) was used to perform Student's *t*-test and Dunnett's test to determine the significance between the hydrolyzed corn starch-treated and control groups. Data are presented as the mean ± SD and "n" depicts the number of biological replicates in each experiment. Values of $p < 0.05$ were considered significant.

Results

Effect of hydrolyzed corn starch treatment on the gene-expression profiles

The statistical comparison of control cells and those treated with 2.0% hydrolyzed corn starch for 24 h according to the Rank Products method with multiple testing corrections using the Benjamini and Hochberg FDR ($p < 0.15$) resulted in the selection of 73 probes (S1 Table~~data not shown~~). Subsequently, we used the online Database for Annotation, Visualization, and Integrated Discovery software module to identify the overrepresented Keywords and Gene Ontology (GO) terms in the selected genes. A functional analysis of the selected 73 probes demonstrated that the effects of hydrolyzed corn starch treatment indicated significant involvement in the oxidation reduction (KW-0560, GO:0016491). In addition, 14 genes related to the oxidation reduction were found (Table 1). Among these genes, *NQO1*, Aldo-keto reductase family 1 member C1 (*AKR1C1*), Aldo-keto reductase family 1 member C2 (*AKR1C2*~~,~~), Aldo-keto reductase family 1 member C3 (*AKR1C3*), Cytochrome P450 family 1 subfamily A member 1 (*CYP4F11*), *GPX2*, and *HMOX1* were upregulated more than 2-fold by treatment with hydrolyzed corn starch. We focused on *HMOX1*, *GPX2*, and *NQO1*, which are commonly regulated by NRF2. To confirm the reproducibility of the microarray data on upregulation of these genes by hydrolyzed corn starch treatment, the mRNA expressions of *HMOX1*, *GPX2*, and *NQO1* in keratinocytes were measured by ~~real-time~~ reverse transcription quantitative polymerase chain reaction (RT-qPCR) (Fig ~~1~~2A). The findings indicated a concentration-dependent increase in mRNA expressions when treated with 1.0%, 2.0%, and 4.0% hydrolyzed corn starch for 24 h.

Furthermore, we examined the effect of hydrolyzed corn starch at different exposure times (3, 6, 10, 24, and 48 h) on the mRNA expression of these enzymes in keratinocytes (Figs ~~1~~2B-D). Hydrolyzed corn starch increased the mRNA expression of *HMOX1*, *GPX2*, and *NQO1* at different times in a gene dependent manner.

To further characterize the antioxidant response to hydrolyzed corn starch, additional antioxidant-related gene expression analyses were performed. The mRNA expression levels of *PRDX1*, *SOD1*, *CAT*, *GSR*, *GCLC*, and *TXN* were determined by RT-qPCR after 24 h treatment with 1.0%, 2.0%, and 4.0% hydrolyzed corn starch (S1 Fig). As shown in S1 Fig, the mRNA expression levels of *GCLC* and *TXN* were upregulated in a concentration-dependent manner, and that of *PRDX1* was significantly increased at 4.0% hydrolyzed corn starch compared with control (*$p < 0.05$). In contrast, *SOD1*, *CAT*, and *GSR* showed no significant changes under these conditions.

Table 1. Oxidation reduction-related genes

UNIGENE	GENE NAME
Hs.406515	NAD(P)H quinone dehydrogenase 1 (*NQO1*)
Hs.460260	Aldo-keto reductase family 1 member C1 (*AKR1C1*)
Hs.460260, Hs.567256	Aldo-keto reductase family 1 member C2 (*AKR1C2*)
Hs.78183	Aldo-keto reductase family 1 member C3 (*AKR1C3*)
Hs.72912	Cytochrome P450 family 1 subfamily A member 1 (*CYP1A1*)
Hs.187393	Cytochrome P450 family 4 subfamily F member 11 (*CYP4F11*)
Hs.179608	Dehydrogenase/reductase 9 (*DHRS9*)
Hs.2704	Glutathione peroxidase 2 (*GPX2*)
Hs.517581	Heme oxygenase 1 (*HMOX1*)
Hs.596461	Isocitrate dehydrogenase (NADP(+)) 2 (*IDH2*)
Hs.464071	Phosphogluconate dehydrogenase (*PGD*)
Hs.495728	Pirin (*PIR*)

Hs.196384	Prostaglandin-endoperoxide synthase 2 (*PTGS2*)
Hs.654922	Thioredoxin reductase 1 (*TXNRD1*)

Fig ~~1~~2. Effects of hydrolyzed corn starch on the mRNA expression levels of the oxidative stress-related genes in keratinocytes.

(A) Cells were treated with 1.0%, 2.0%, and 4.0% hydrolyzed corn starch for 24 h. (B-D) Cells were treated with 2.0% hydrolyzed corn starch for 3, 6, 10, 24, and 48 h. The mRNA levels of ~~heme oxygenase (~~*HMOX*~~)-~~*1* (~~C~~B), ~~glutathione peroxidase (~~*GPX2*~~)~~ (~~D~~C), and ~~NAD(P)H: quinone oxidoreductase (~~*NQO*~~)-~~*1* (~~C~~D) were determined by ~~quantitative real-time polymerase chain reaction~~RT-qPCR and normalized to that of ~~glyceraldehyde 3-phosphate dehydrogenase (~~*GAPDH*~~) (n = 3)~~. Dunnett's test, **p* < 0.05, ***p* < 0.01, ****p* < 0.001 (n = 3).

Effects of hydrolyzed corn starch on the ROS level in keratinocytes exposed to hydrogen peroxide (H_2O_2)

To evaluate the antioxidant response to treatment with 2.0% and 4.0% hydrolyzed corn starch for 24 and 48 h, intracellular ROS levels were measured using the fluorescent probe DCFH-DA (Figs ~~2~~3A and 3B). As shown in Figs ~~2A~~3A and 3B, compared with the H_2O_2-untreated group, the intracellular ROS production was increased in the H_2O_2-treated group. Pretreatment with the hydrolyzed corn starch inhibited the increase in ROS production induced by H_2O_2-treatment, and 4.0% hydrolyzed corn starch after 48 h exposure significantly inhibited ROS production relative to that of the control ($p < 0.001$) (Fig 3B). Typical fluorescence images of the cells are shown in Fig ~~2B~~3C. The fluorescence caused by the oxidation of DCFH-DA was increased by H_2O_2, and the change was reduced by hydrolyzed corn starch.

Fig ~~2~~3. Effects of hydrolyzed corn starch on the reactive oxygen species (ROS) production in keratinocytes exposed to H_2O_2.

Formatted: Font: Italic

Formatted: Font: Italic

Formatted: Font: Italic

Formatted: Font: Italic

Formatted: Font: Italic

Formatted: Font: Italic

Formatted: Font: Italic

(A, B) Cells were labeled with DCFH-DA, treated with 2.0% and 4.0% hydrolyzed corn starch for 24 h (A) and 48 h (B), and exposed to 200 μM H_2O_2 for 1 h~~,~~. ~~and t~~ The fluorescence intensity of the cells was measured to determine the ROS levels~~ (A)~~. (C) Typical images of cells fluorescing due to the oxidation of DCFH-DA by ROS~~ are shown in (B)~~. Dunnett's test, ****p* < 0.001 (n = 3).

Effects of hydrolyzed corn starch on NRF2 activation

The activation of NRF2 occurs by translocating NRF2 into the nucleus from the cytosol. After NRF2 translocates into the nucleus, it can bind to the ARE and drive the expression of target genes, such as *HMOX1*, *GPX2*, and *NQO1*.

To analyze the protein level of NRF2 in the nucleus, we performed western blotting on cells treated with 4.0% hydrolyzed corn starch for 2 h. β-acti~~o~~n served as a reference protein for normalization. As shown in Fig ~~3A~~4A, 4.0% hydrolyzed corn starch increased the protein levels of NRF2 in the nucleus, and 4.0% hydrolyzed corn starch significantly increased NRF2 in the nucleus compared with that in the control ($p < 0.05$). Furthermore, Fig ~~3B~~4B shows that NRF2 is localized from the cytosol to the nucleus in the cells treated with 4.0% hydrolyzed corn starch for 2 h.

Fig ~~3~~4. Effects of hydrolyzed corn starch on NRF2 activation in keratinocytes.

(A) Cells were treated with 4.0% hydrolyzed corn starch for 2 h, and nuclear NRF2 protein levels ~~(A)~~ were determined by western blotting and normalized to that of nuclear β-actin. ~~Representative blots are shown in (A).~~ Student's *t*-test, **p* < 0.05 (n = 3). (B) Nuclear translocation of NRF2 was determined by immunostaining. ~~(B). Student's *t*-test, *p < 0.05 (n = 3).~~

Formatted: Not Highlight

Discussion

The skin generates ROS in response to external stimuli, such as ultraviolet radiation and air pollution. The transcription factor NRF2 plays a central role in the cellular defense mechanism against oxidative stress. Upon translocation from the cytoplasm to the nucleus, NRF2 binds to the ARE and upregulates genes, including *HMOX1*, *GPX2*, and *NQO1*. The *HMOX1* gene encodes heme oxygenase-

1, which is a key enzyme that catalyzes the rate-limiting first step of heme degradation, yielding carbon monoxide, ferrous, and biliverdin. Biliverdin is rapidly reduced to bilirubin, a known strong antioxidant [~~10~~14]. *GPX2* reduces hydrogen peroxide and lipid hydroperoxides at the expense of glutathione, whereas *NQO1* reduces quinone to hydroquinone. These antioxidant enzymes act both directly and indirectly to help alleviate oxidative stress in the long term [~~11~~15]. Conversely, when ROS levels become excessively high, oxidative damage to DNA and proteins can occur along with increased synthesis and activation of matrix metalloproteinases, enzymes that degrade extracellular matrix components, and higher ROS levels are thought to promote collagen and elastin degradation, leading to skin aging, such as wrinkles and sagging [~~12, 13~~16, 17]. Furthermore, ROS stimulate keratinocytes and release~~s~~ various melanocyte activating factors, such as endothelin-1 and protease-activated receptor-2, which is known to promote melanin synthesis and melanosome transport [~~14, 15~~18, 19], and is thought to cause skin problems, such as age spots and pigmentation.

In this study, we demonstrated that hydrolyzed corn starch activates the NRF2 pathway in keratinocytes, leading to increase the expression of antioxidant-responsive genes and a subsequent reduction in ROS levels. Specifically, NRF2 activation occurred 2 h after the application of hydrolyzed corn starch, subsequently leading to ≥2-fold changes in *NQO1* at 6 h, *HMOX1* at 10 h, and *GPX2* at 48 h and ultimately leading to the inhibition of H_2O_2-dependent ROS generation at 48 h. These findings indicate that hydrolyzed corn starch enhances the cellular defense capacity against oxidative stress via the NRF2 pathway in keratinocytes.

In addition, previous studies have suggested that the expressions of *GCLC*, *TXN*, *PRDX1*, *SOD1*, *CAT*, and *GSR* can be regulated by NRF2 [2, 20, 21], although the extent of NRF2 involvement may vary depending on the cell type and experimental conditions. In our study, treatment with 1.0%, 2.0%, and 4.0% hydrolyzed corn starch for 24 h upregulated the mRNA expression levels of *GCLC* and *TXN* in a concentration-dependent manner and significantly increased that of *PRDX1* at 4.0%, whereas *SOD1*, *CAT*, and *GSR* showed no significant changes under these conditions. Taken together with the

Formatted: Not Highlight
Formatted: Not Highlight
Formatted: Not Highlight
Formatted: Font: Italic
Formatted: Font: Italic
Formatted: Font: Italic
Formatted: Font: Italic
Formatted: Font: Italic
Formatted: Not Highlight
Formatted: Not Highlight
Formatted: Font: Italic
Formatted: Font: Italic
Formatted: Font: Italic
Formatted: Font: Italic
Formatted: Font: Italic
Formatted: Font: Italic

observed induction of *HMOX1*, *GPX2*, and *NQO1*, these additional changes in antioxidant-related genes further support the possibility that the overall gene-expression profile induced by hydrolyzed corn starch is, at least in part, a consequence of NRF2 activation, although contributions from other transcriptional regulators cannot be excluded.

Given that excessive ROS are known to contribute to collagen and elastin degradation and to hyperpigmentation through the activation of melanocyte-stimulation factors [16-19], NRF2 activation and ROS reduction by hydrolyzed corn starch raise the possibility that this ingredient may indirectly influence skin conditions such as wrinkles, sagging, and pigmentation. However, our current data are limited to antioxidant responses in keratinocytes, and we did not directly assess structural or functional endpoints (e.g., collagen content, skin elasticity, melanin production) in the skin. Therefore, any anti-wrinkle, anti-sagging, or depigmenting effects remain speculative and should be validated in future *in vitro* and clinical studies. In addition, our experiments were conducted exclusively in keratinocytes and did not address the role of dermal fibroblasts, which are crucial for extracellular matrix production and skin elasticity. Future investigations using dermal fibroblasts, melanocyte-keratinocyte co-culture systems, and *in vivo* or clinical evaluations will be required to clarify whether the NRF2 activation and ROS reduction observed here translate into measurable improvements in these skin conditions. ~~This study's results suggest that hydrolyzed corn starch enhances the skin's defense ability against oxidative stress by activating NRF2. Therefore, hydrolyzed corn starch may mitigate skin issues, such as wrinkles, sagging, and pigmentation by reducing ROS levels.~~

~~Specifically, p~~People with sensitive skin often have low-barrier skin function, making them susceptible to external stimuli and increased oxidative stress. The multifunctional profile of hydrolyzed corn starch, including moisturizing, itch-relief, and antioxidant effects via NRF2 activation, supports its potential application as a cosmetic ingredient for sensitive skin.

Moreover, recent studies have demonstrated that activation of the NRF2 pathway can suppress inflammation by inhibiting the expression of interleukin 6 and interleukin 1β, cytokines that exacerbate

Formatted: Font: Italic
Formatted: Font: Italic
Formatted: Font: Italic
Formatted: Indent: First line: 0.19"
Formatted: Font: Italic
Formatted: Font: Italic
Formatted: Indent: First line: 1 ch

inflammation [16]. On the basis of these reports, the NRF2 activation observed in our study raises the possibility ~~suggesting~~ that hydrolyzed corn starch may have anti-inflammatory effects on the skin. However, we did not measure inflammatory markers, cytokine expression, or other indices of inflammation in our experiments. Thus, any anti-inflammatory effect of hydrolyzed corn starch should be regarded as a hypothesis derived from the known function of NRF2 rather than as an observed effect.

~~Notably, this study revealed that NRF2 activation occurred 2 h after the application of hydrolyzed corn starch, subsequently leading to ≥2-fold gene-expression changes in *NQO1* from 6 h, *HMOX1* from 10 h, and *GPX2* from 48 h and ultimately resulting in ROS reduction at 48 h. Although these findings suggested that MTO~~ Our results also suggest that maltotetraose (MTO), a major component of hydrolyzed corn starch, may play~~s~~ a key role in NRF2 activation and subsequent ROS reduction in keratinocytes,~~these functions,~~ although the detailed mechanism remains unclear. Whether ROS reduction is solely mediated by the NRF2 pathway and the specific mechanism by which MTO activates NRF2 are not yet established. In addition, we did not evaluate the overall status of enzymatic and non-enzymatic antioxidant capacity in this study, and thus the global impact of MTO on the cutaneous antioxidant defense system remains unclear. A comprehensive understanding will require the identification of the individual compounds in the hydrolyzed corn starch, subsequent validation using each individual compound, and elucidation of the associated molecular interactions and signaling pathways with specific inhibitors. ~~Further clinical trials are warranted to validate these effects in human.~~

Conclusion

The main focus of this study was to evaluate the potential novel beneficial effects of hydrolyzed corn starch containing maltotetraose on the skin, particularly defense against oxidative stress. The results suggest that hydrolyzed corn starch enhances cellular antioxidant capacity through induction of antioxidant-responsive genes, thereby~~,~~ contributing to the reduction of ROS levels. Furthermore, the results also suggest that hydrolyzed corn starch enhances cellular defense against oxidative stress by

NRF2 activation.

Acknowledgments

We thank Dr. Tetsuji Hirao (Professor, School of Pharmacy and Pharmaceutical Sciences, Mukogawa Women's University), for valuable technical advice and support throughout this study.

Data ~~Set~~ Availability

~~The datasets used and/or analysed during the current study are available from the corresponding author on reasonable request.~~

All microarray data files have been deposited in the NCBI Gene Expression Omnibus (GEO) under accession number GSEXXXXXXX (to be updated once the accession number is assigned). All other datasets used for the statistical analyses, including individual data points and sample sizes for each experiment, have been deposited in the Zenodo repository and are freely available under the accession DOI 10.5281/zenodo.19227766. All other relevant data are within the manuscript and its Supporting Information files.

~~Funding~~

~~This research was supported financially by the Lion Corporation. No other specific grant was received from funding agencies in the public, commercial, or not-for-profit sectors.~~

Formatted: Font color: Text 1

Formatted: Font: Not Bold, Font color: Text 1, Highlight

Formatted: Font color: Text 1, Highlight

Formatted: Font color: Text 1

Formatted: Font: Not Bold, Font color: Text 1, Not Highlight

Formatted: Font color: Text 1

Formatted: Font: Bold, Font color: Text 1, Highlight

References

1. Jaffri JM. Reactive oxygen species and antioxidant system in selected skin disorders. Malays J Med Sci. 2023;30: 7
2. Ngo V, Duennwald ML. Nrf2 and oxidative stress: A general overview of mechanisms and implications in human disease. Antioxidants. 2022;11: 2345
3. Frants MC, Rozot R, Marrot L. NRF2 in dermo-cosmetic: From scientific knowledge to skin care products. BioFactors. 2023;49: 32-61.
4. Chen J, Liu Y, Zhao Z, Qiu J. Oxidative stress in the skin: Impact and related protection. Int J Cosmet Sci. 2021;43: 495-509
5. Schäfer M, Werner S. Nrf2-A regulator of keratinocyte redox signaling. Free Radical Biology and Medicine. 2015;88: 243-252
6. Salman S, Paulet V, Hardonniére K, Kerdine-Römer S. The role of NRF2 transcription factor in inflammatory skin diseases. Biofactors. 2025;51(2)

Formatted: Font: (Default) Times New Roman, 12 pt
Formatted: Font: (Default) Times New Roman, 12 pt
Formatted: Font: (Default) Times New Roman, 12 pt
Formatted: Font: (Default) Times New Roman, 12 pt

7. Kim HJ, Hong JH. Emerging Therapeutic Strategies for Nrf2-Associated Skin Disorders: From Photoaging to Autoimmunity. Antioxidants. 2026;15(1): 69

~~4.~~8. Andersen FA, Bergfeld WF, Belsito DV, Klaassen CD, Marks JG Jr, Shank RC, et al. Final Report of the Safety Assessment of Cosmetic Ingredients Derived From Zea Mays (Corn). International Journal of Toxicology. 2011;30(Supplement 1): 17S-39S

Formatted: Font: (Default) Times New Roman, 12 pt

~~5.~~9. Jiang C, Guo C, Yan J, Chen J, Peng S, Huang H, et al. Sensitive skin syndrome: Research progress on mechanisms and applications. J Dermatol Sci Cosmet Technol. 2024;1: 100015.

~~6.~~10. Ständer S, Yosipovitch G. Substance P and neurokinin 1 receptor are new targets for the treatment of chronic pruritus. Br J Dermatol. 2019;81: 932-938.

~~7.~~11. Alam M, Buddenkotte J, Ahmad F, Steinhoff M. Neurokinin 1 receptor antagonists for pruritus. Drugs. 2021;81: 621-634.

~~8.~~12. Uozumi T, Morishita S, Tanaka Y, Yumoto M. NK1 receptor antagonist composition. US Patent

8426386 B2, April 23, 2013.

~~9.~~13. Ichikawa E, Inoue A, Matsuzaki K, Kaneda S, Nito A, Yokoyama M. Clinical evaluation of the efficacy of itch-relief moisturizers containing maltotetraose for dry, itchy, and sensitive skin. Skinmed. 2024;22: 187-196.

~~10.~~14. Stocker R, Yamamoto Y, McDonagh AF, Glazer AN, Ames BN. Bilirubin is an antioxidant of possible physiological importance. Science. 1987;235: 1043-1046.

~~11.~~15. Kensler TW, Wakabayashi N, Biswal S. Cell survival responses to environmental stresses via the Keap1-Nrf2-ARE pathway. Annu Rev Pharmacol Toxicol. 2007;47: 89-116

~~12.~~16. Fisher GJ, Datta SC, Talwar HS, Wang ZQ, Varani J, Kang S, et al. Molecular basis of sun-induced premature skin ageing and retinoid antagonism**.** Nature. 1996;379: 335-339.

~~13.~~17. Fisher GJ, Talwar HS, Lin J, Lin P, McPhillips F, Wang Z, et al. Retinoic acid inhibits induction of c-Jun protein by ultraviolet radiation that occurs subsequent to activation of mitogen-activated protein kinase pathways in human skin in vivo. J Clin Invest. 1998;101: 1432-1440.

~~14.~~18. Imokawa G, Yada Y, Miyagishi M. Endothelins secreted from human keratinocytes are intrinsic mitogens for human melanocytes. J Biol Chem. 1992;267: 24675-24680.

19. Li J, Tang LY, Fu WW, Yuan J, Sheng YY, Yang QP. Low-concentration hydrogen peroxide can upregulate keratinocyte intracellular calcium and PAR-2 expression in a human keratinocyte–melanocyte co-culture system. Arch Dermatol Res. 2016;308: 723-731.

20. He F, Ru X, Wen T. NRF2, a Transcription Factor for Stress Response and Beyond. Int J Mol Sci. 2020;21(13): 4777

~~15.~~21. Jung K, Kwak Mi. The Nrf2 System as a Potential Target for the Development of Indirect Antioxidants. Molecules. 2010;15(10): 7266-7291

22. Kobayashi EH, Suzuki T, Funayama R, Nagashima T, Hayashi M, Sekine H, et al. Nrf2 suppresses macrophage inflammatory response by blocking proinflammatory cytokine transcription. Nat Commun. 2016;7: 11624.

Supporting information

S1 Table. 73 differentially expressed probes. Cells were treated with 2.0% hydrolyzed corn starch for 24 h. Differentially expressed probes were identified by microarray analysis.

S1 Fig. Effects of hydrolyzed corn starch on the mRNA expression levels of antioxidant related genes. Cells were treated with 1.0%, 2.0%, and 4.0% hydrolyzed corn starch for 24 h.

~~16.~~

Formatted: Font: (Default) Times New Roman, 18 pt, Bold

Formatted: Font: 12 pt

Formatted: Font: Bold

Formatted: Not Highlight

Formatted: Font: Bold, Not Highlight

Formatted: Not Highlight

Formatted: Font: Bold

Formatted: Not Highlight

Formatted: Font: (Default) Times New Roman, 12 pt

Formatted: Normal, No bullets or numbering

[Point-by-point response] (Black: Editor's and Reviewer's comments, Red: our responses)

Journal Requirement (Editor)

1. Please ensure that your manuscript meets PLOS ONE's style requirements, including those for file naming. The PLOS ONE style templates can be found at https://journals.plos.org/plosone/s/file?id=wjVg/PLOSOne_formatting_sample_main_body.pdf and https://journals.plos.org/plosone/s/file?id=ba62/PLOSOne_formatting_sample_title_authors_affiliations.pdf.

We have carefully revised the manuscript to ensure that it follows the PLOS ONE style requirements, including those for file naming and formatting. We have consulted and applied the formatting guidelines provided in the PLOS ONE style templates.

2. Thank you for stating in your Funding Statement: This research was supported financially by the Lion Corporation. No other specific grant was received from funding agencies in the public, commercial, or not-for-profit sectors." Please provide an amended statement that declares *all* the funding or sources of support (whether external or internal to your organization) received during this study, as detailed online in our guide for authors at http://journals.plos.org/plosone/s/submit-now. Please also include the statement "There was no additional external funding received for this study." in your updated Funding Statement.

Please include your amended Funding Statement within your cover letter. We will change the online submission form on your behalf.

We appreciate the editor's guidance regarding the Funding Statement. We have revised the Funding Statement to read as follows, and will include this updated version in the cover letter:

"This research was supported financially by the Lion Corporation. No other specific grant was received from funding agencies in the public, commercial, or not-for-profit sectors. There was no additional external funding received for this study."

We understand that the online submission form will be updated by the editorial office based on the text provided in the cover letter.

3. Thank you for stating the following financial disclosure:

“This research was supported financially by the Lion Corporation. No other specific grant was received from funding agencies in the public, commercial, or not-for-profit sectors.” Please state what role the funders took in the study. If the funders had no role, please state: "The funders had no role in study design, data collection and analysis, decision to publish, or preparation of the manuscript." If this statement is not correct you must amend it as needed. Please include this amended Role of Funder statement in your cover letter; we will change the online submission form on your behalf.

We thank the editor for pointing out the need to clarify the role of the funder.

In accordance with the journal’s request, we have added the following statement regarding the role of the funder, which will be included in the cover letter:

"The funders had no role in study design, data collection and analysis, decision to publish, or preparation of the manuscript."

We understand that the editorial office will update the corresponding section in the online submission system based on this statement.

4. We note that you have indicated that there are restrictions to data sharing for this study. For studies involving human research participant data or other sensitive data, we encourage authors to share de-identified or anonymized data. However, when data cannot be publicly shared for ethical reasons, we allow authors to make their data sets available upon request. For information on unacceptable data access restrictions, please see http://journals.plos.org/plosone/s/data-availability#loc-unacceptable-data-access-restrictions. Before we proceed with your manuscript, please address the following prompts:

a) If there are ethical or legal restrictions on sharing a de-identified data set, please explain them in detail (e.g., data contain potentially identifying or sensitive patient information, data are owned by a third-party organization, etc.) and who has imposed them (e.g., a Research Ethics Committee or Institutional Review Board, etc.). Please also provide contact information for a data access committee, ethics committee, or other institutional body to which data requests may be sent.

b) If there are no restrictions, please upload the minimal anonymized data set necessary to replicate your study findings to a stable, public repository and provide us with the relevant URLs, DOIs, or accession numbers. Please see http://www.bmj.com/content/340/bmj.c181.long

for guidelines on how to de-identify and prepare clinical data for publication. For a list of recommended repositories, please see https://journals.plos.org/plosone/s/recommended-repositories. You also have the option of uploading the data as Supporting Information files, but we would recommend depositing data directly to a data repository if possible.

Please update your Data Availability statement in the submission form accordingly.

We have revised the Data Availability statement in the manuscript to more clearly describe where the data can be accessed. The revised statement reads as follows: However, the microarray data have not yet been assigned an accession number in GEO. The revised statement reads as follows:

"All microarray data files have been deposited in the NCBI Gene Expression Omnibus (GEO) under accession number GSEXXXXXX (to be updated once the accession number is assigned). All other datasets used for the statistical analyses, including individual data points and sample sizes for each experiment, have been deposited in the Zenodo repository and are freely available under the accession DOI 10.5281/zenodo.19227766. All other relevant data are within the manuscript and its Supporting Information files." (line 372–380)

We hope that this revision sufficiently clarifies the sample sizes and the accessibility of all datasets used in our analyses and ensures compliance with the PLOS ONE Data Availability policy.

5. In the online submission form you indicate that your data is not available for proprietary reasons and have provided a contact point for accessing this data. Please note that your current contact point is a co-author on this manuscript. According to our Data Policy, the contact point must not be an author on the manuscript and must be an institutional contact, ideally not an individual. Please revise your data statement to a non-author institutional point of contact, such as a data access or ethics committee, and send this to us via return email. Please also include contact information for the third party organization, and please include the full citation of where the data can be found.

In response to the journal's data policy, we have revised our data sharing plan so that the underlying datasets are now publicly available and no longer subject to proprietary access restrictions.

As noted above, the microarray data have been submitted to the NCBI Gene Expression

Omnibus (GEO) and will be available under accession GSEXXXXXX once it is assigned. All other datasets used for statistical analyses have been deposited in Zenodo (DOI 10.5281/zenodo.19227766). Therefore, we no longer rely on a "data available upon request" model, and no author will serve as a direct data-access contact point.

Because the data are openly available through these public repositories, an additional institutional contact point for data access is no longer required.

6. Please be informed that funding information should not appear in the Acknowledgments section or other areas of your manuscript. We will only publish funding information present in the Funding Statement section of the online submission form. Please remove any funding-related text from the manuscript.

We acknowledge the journal's policy that funding information should only appear in the Funding Statement section of the online submission form and not in the Acknowledgments or other parts of the manuscript. Accordingly, we have removed all funding-related text from the Acknowledgments section and from any other location in the manuscript.

7. We note that you have included the phrase "data not shown" in your manuscript. Unfortunately, this does not meet our data sharing requirements. PLOS does not permit references to inaccessible data. We require that authors provide all relevant data within the paper, Supporting Information files, or in an acceptable, public repository. Please add a citation to support this phrase or upload the data that corresponds with these findings to a stable repository (such as Figshare or Dryad) and provide and URLs, DOIs, or accession numbers that may be used to access these data. Or, if the data are not a core part of the research being presented in your study, we ask that you remove the phrase that refers to these data.

We thank the editor and reviewers for drawing our attention to the use of the phrase "data not shown" and for emphasizing the importance of data transparency. In response to the concern that "data not shown" is not acceptable for key results and that the underlying data must be available, we have now provided the supporting microarray data for the 73 selected probes as a supplementary file (S1 Table). These changes ensure that all relevant data supporting our findings are accessible to readers and fully comply with PLOS ONE's data sharing requirements.

8. PLOS ONE now requires that authors provide the original uncropped and unadjusted images underlying all blot or gel results reported in a submission's figures or Supporting Information

files. This policy and the journal's other requirements for blot/gel reporting and figure preparation are described in detail at https://journals.plos.org/plosone/s/figures#loc-blot-and-gel-reporting-requirements and https://journals.plos.org/plosone/s/figures#loc-preparing-figures-from-image-files. When you submit your revised manuscript, please ensure that your figures adhere fully to these guidelines and provide the original underlying images for all blot or gel data reported in your submission. See the following link for instructions on providing the original image data: https://journals.plos.org/plosone/s/figures#loc-original-images-for-blots-and-gels. In your cover letter, please note whether your blot/gel image data are in Supporting Information or posted at a public data repository, provide the repository URL if relevant, and provide specific details as to which raw blot/gel images, if any, are not available. Email us at plosone@plos.org if you have any questions.

We understand and appreciate the journal's policy requiring the submission of original, uncropped, and unadjusted images for all blot and gel data. In accordance with this policy, we have prepared and submitted the raw Western blot images underlying Figure 4 as Supporting information (S1_raw_images.pdf). These files correspond directly to the processed images shown in Figure 4. We will clearly indicate in the cover letter that the raw blot images for Figure 4 have been provided, and we confirm that there are no additional blot or gel images in the manuscript for which raw data are unavailable.

9. If the reviewer comments include a recommendation to cite specific previously published works, please review and evaluate these publications to determine whether they are relevant and should be cited. There is no requirement to cite these works unless the editor has indicated otherwise.

We note that the reviewers did not explicitly recommend any specific previously published works that must be cited in this manuscript. Accordingly, no reviewer-suggested citations needed to be added. We have, however, reviewed and updated the references in the Introduction and Discussion where appropriate to ensure that the most relevant and recent literature is cited.

Reviewer #1

1. Repetition of the phrase "hydrolyzed corn starch containing maltotetraose" affects flow and should be reduced in the Abstract section.

We appreciate this helpful comment. In the revised Abstract, we now use the full expression "hydrolyzed corn starch containing maltotetraose" only when the ingredient is first introduced and again in the concluding sentence where we summarize its significance. In the rest of the Abstract, we refer to it more concisely as "this ingredient (line: 24 and 32)," "it," (line: 25) or "the ingredient" (line: 29-30) where the meaning is clear. We believe this has reduced redundancy and improved the readability of the Abstract.

2. No pigmentation-related markers, melanocyte models, or aging-associated endpoints are mentioned.

We appreciate insightful comments. We fully agree that our current study did not include pigmentation-related markers, melanocyte-based models, or aging-associated endpoints, and that any statements regarding pigmentation or aging must therefore be made with caution.

In response to this comment, we have revised the Abstract and Discussion to clearly state that the data presented in this study are limited to antioxidant responses and NRF2 activation in keratinocytes. Any potential implications for pigmentation or aging are now explicitly described as hypothetical and not directly evaluated in this work. In the Abstract we now state:

"This NRF2-mediated antioxidative activity positions hydrolyzed corn starch containing maltotetraose as a promising ingredient for cosmetic products, with potential implications for improving skin health, reducing pigmentation, and combating aging. However, these effects were not directly evaluated in this study. Future studies are warranted to evaluate its efficacy *in vivo*." (line: 33-38)

In the Discussion, we also note that:

"However, our current data are limited to antioxidant responses in keratinocytes, and we did not directly assess structural or functional endpoints (e.g., collagen content, skin elasticity, melanin production) in the skin. Therefore, any anti-wrinkle, anti-sagging, or depigmenting effects remain speculative and should be validated in future *in vitro* and clinical studies." (line: 324-328)

We hope that these revisions clarify the scope and limitations of our study.

3. In line 32, the phrase “novel functionality” is vague and should be replaced with more precise scientific language.

We appreciate this helpful comment. We agree that the term “novel functionality” was vague. In the revised manuscript, we have replaced this phrase with the more specific and scientifically accurate term “NRF2-mediated antioxidative activity,” (line: 34) which directly reflects the mechanism demonstrated in our study. The sentence now reads:

“This NRF2-mediated antioxidative activity positions hydrolyzed corn starch containing maltotetraose as a promising ingredient for cosmetic products, with potential implications for improving skin health, reducing pigmentation, and combating aging.” (line: 33-36)

This revision clarifies that the functionality in question is the activation of the NRF2 pathway leading to an enhanced antioxidative response.

4. Typographical error identified: “maltotetoraose” should be corrected to “maltotetraose” in line 33.

We thank the reviewer for pointing out this typographical error. In the revised manuscript, we have corrected the misspelling “maltotetoraose” to “maltotetraose” on line 35. We have also checked the entire manuscript to ensure that the term “maltotetraose” is spelled consistently throughout.

5. In line 56, the statement that NRF2 is a “promising target in cosmetic applications” is plausible but under-argued. Clarify why moderate NRF2 activation is beneficial and safe in cosmetic formulations.

We appreciate this valuable comment. We agree that additional explanation was needed to clarify why NRF2 can be regarded as a beneficial and safe target in the context of cosmetic formulations.

In the revised Introduction, we have elaborated on the role of NRF2 in skin and the rationale for targeting it in cosmetics. (line: 59-67) Specifically, we now emphasize that:

•NRF2 regulates a broad range of antioxidant and phase II detoxification enzymes that detoxify and eliminate reactive oxygen species and other oxidative agents, thereby contributing to photoprotection, regulation of pigmentation, wound healing, and reinforcement of epidermal

barrier function [3,5-7]. Enhancement of NRF2 signaling is therefore expected to strengthen endogenous antioxidant and barrier defenses, thereby helping to prevent photoaging, pigmentation disorders, and barrier impairment induced by environmental stressors.

•Transient NRF2 activation is unlikely to be harmful, as NRF2 upregulates Keap1 expression via a negative feedback mechanism that contributes to the maintenance of physiological redox balance in cells.

We hope that this clarification addresses the concern regarding why moderate NRF2 activation can be considered both beneficial and safe in cosmetic formulations.

6. "Raw and functional cosmetic ingredient" is vague. Consider replacing with "naturally derived functional cosmetic ingredient" in line 57.

Thank you for pointing this out. We agree that the previous wording was ambiguous. In the revised manuscript, we have replaced "raw and functional cosmetic ingredient" with a clearer description. The relevant sentence now reads: "We have identified hydrolyzed corn starch, derived from corn, as a safe and effective naturally derived functional cosmetic ingredient." (line: 71-72)

This wording better reflects both the natural origin and the functional role of the ingredient in cosmetic formulations.

7. The assertion that MTO has "superior skin-moisturizing properties" based on hydroxyl group content alone is chemically simplistic. Provide experimental or literature-based evidence supporting superior humectancy, or rephrase to a more cautious claim (e.g., "potentially enhanced moisturizing capacity") in line 60.

We fully agree that our initial wording was too strong given that it was based only on the number of hydroxyl groups. In accordance with your suggestion, we have revised this part of the text to adopt a more cautious wording. The sentence now reads:

"Given its higher concentration of hydroxyl groups compared to other humectants, such as glycerin and butylene glycol, MTO has potentially enhanced moisturizing capacity." (line: 74-76)

By using the phrase "potentially enhanced moisturizing capacity," we avoid overinterpreting the chemical structure alone and clearly indicate that superior moisturizing efficacy is a possibility

rather than an established fact.

Where relevant, we refer to our clinical study demonstrating moisturizing and barrier-improving effects of an MTO-containing moisturizer [13], which supports the practical usefulness of MTO in skin hydration without attributing this solely to the number of hydroxyl groups.

8. In line 184, the term "robes" appears to be a typographical error and should be corrected to "probes". The phrase "data not shown" is not acceptable for a primary result in a manuscript. Transparency requires that the underlying data be accessible.

We thank the reviewer for carefully pointing out the typographical error and the issue regarding data transparency.

First, the spelling error "robes" has been corrected to "probes" in the revised manuscript. (line: 219)

Second, in response to the reviewer's comment that "data not shown" is not acceptable for major results and that the underlying data should be accessible, we have now provided the supporting microarray data (S1 Table) for the selected 73 probes. (line: 219)

9. Reporting only the number of selected probes (n = 73) without presenting any supporting data (e.g., fold changes, adjusted p-values, or gene identities) is insufficient. At a minimum, a supplementary table should be provided.

We have added S1 Table, which lists, for each of the 73 probes, the gene name, Unigene, fold change, and p-value. We believe these changes improve the transparency and reproducibility of our analysis and adequately address the reviewer's concern.

10. The discussion appropriately outlines the NRF2–ARE antioxidant pathway and its relevance to skin oxidative stress. However, the conclusion that hydrolyzed corn starch mitigates wrinkles, sagging, and pigmentation is speculative and not directly supported by the presented data.

We agree that our original wording could be interpreted as claiming direct evidence for anti-wrinkle, anti-sagging, and depigmenting effects, which is not supported by our current dataset. Accordingly, we have revised the Discussion to clearly distinguish between our experimental findings and potential implications.

The following paragraphs were added:

"Given that excessive ROS are known to contribute to collagen and elastin degradation and to hyperpigmentation through the activation of melanocyte-stimulation factors [16-19], NRF2

activation and ROS reduction by hydrolyzed corn starch raise the possibility that this ingredient may indirectly influence skin conditions such as wrinkles, sagging, and pigmentation. However, our current data are limited to antioxidant responses in keratinocytes, and we did not directly assess structural or functional endpoints (e.g., collagen content, skin elasticity, melanin production) in the skin. Therefore, any anti-wrinkle, anti-sagging, or depigmenting effects remain speculative and should be validated in future *in vitro* and clinical studies. In addition, our experiments were conducted exclusively in keratinocytes and did not address the role of dermal fibroblasts, which are crucial for extracellular matrix production and skin elasticity. Future investigations using dermal fibroblasts, melanocyte-keratinocyte co-culture systems, and *in vivo* or clinical evaluations will be required to clarify whether the NRF2 activation and ROS reduction observed here translate into measurable improvements in these skin conditions." (line: 321-332)

11. Justify this statement: the anti-inflammatory effect is inferred solely from literature citing NRF2-mediated suppression of IL-6 and IL-1β. Since no inflammatory markers were measured in this study, this conclusion should be framed as a potential implication, not as an observed effect in lines 265-268.

We fully concur with this comment. In the revised Discussion, we explicitly state that any anti-inflammatory effect is hypothetical and derived from known NRF2 functions, not from our own measurements. The relevant part now reads:

"Moreover, recent studies have demonstrated that activation of the NRF2 pathway can suppress inflammation by inhibiting the expression of interleukin 6 and interleukin 1β, cytokines that exacerbate inflammation [16]. On the basis of these reports, the NRF2 activation observed in our study raises the possibility that hydrolyzed corn starch may have anti-inflammatory effects on the skin. However, we did not measure inflammatory markers, cytokine expression, or other indices of inflammation in our experiments. Thus, any anti-inflammatory effect of hydrolyzed corn starch should be regarded as a hypothesis derived from the known function of NRF2 rather than as an observed effect." (line: 340-346)

Reviewer #2

1. Please provide latest references for the second paragraph of the Introduction section.

We appreciate your suggestion to include more recent references supporting the role of NRF2 in skin biology and pathology. In the revised version of the Introduction (second paragraph, line: 50-67), we have added the following key references:

[5] Schäfer M, Werner S. Nrf2-A regulator of keratinocyte redox signaling. Free Radical Biology and Medicine. 2015;88: 243-252

[6] Salman S, Paulet V, Hardonniére K, Kerdine-Römer S. The role of NRF2 transcription factor in inflammatory skin diseases. Biofactors. 2025;51(2)

[7] Kim HJ, Hong JH. Emerging Therapeutic Strategies for Nrf2-Associated Skin Disorders: From Photoaging to Autoimmunity. Antioxidants. 2026;15(1): 69

These references have been incorporated into the second paragraph of the Introduction to better substantiate the description of NRF2 as a central regulator of cutaneous antioxidant and cytoprotective responses, and to align the manuscript with the latest advances in the field.

2. If possible please provide a schematic to explain the pathways during presence and absence of oxidative stress.

Thank you for this helpful suggestion. In the revised manuscript, we have added a schematic illustration of the NRF2–Keap1 pathway under basal and oxidative stress conditions.

The new figure is included as Figure 1 and is described as follows:

"Fig 1. NRF2-Keap1 pathway under basal and oxidative stress conditions.

Under basal conditions, NRF2 is retained in the cytoplasm through its interaction with Keap1, which promotes NRF2 ubiquitination and proteasomal degradation. In the presence of oxidative stress, structural changes in Keap1 lead to the release and stabilization of NRF2. The stabilized NRF2 translocates into the nucleus, where it binds to ARE in the promoters of target genes. This induces the expression of phase II detoxification enzymes, antioxidant enzymes, and other cytoprotective molecules." (line: 90-96)

We believe this schematic will facilitate readers' understanding of the molecular mechanism by which NRF2 contributes to the skin's defense against oxidative stress and will clarify the rationale for targeting NRF2 in our study

3. Is there previously available literature that supports hydrolyzed corn starch or MTO f as a potent cosmetic ingredient?

Thank you for this important comment. We agree that the basis for describing hydrolyzed corn starch as a promising cosmetic ingredient should be clarified.

In the revised manuscript, we have added one sentence in the Introduction to explicitly indicate the official cosmetic classification of hydrolyzed corn starch:

"Hydrolyzed corn starch is officially categorized as a skin conditioning agent, specifically a humectant, in cosmetic ingredient nomenclature [8]." (line: 70-71)

We hope that this clarification in the Introduction, sufficiently addresses the reviewer's concern regarding the existing evidence supporting hydrolyzed corn starch as a cosmetic ingredient.

4. The data of 73 probes can be provided as supplementary information?

We agree with the reviewer that the data for the 73 selected probes should be made available as supplementary information. In the revised manuscript, we have therefore added Supplementary S1 Table, which provides, for each of the 73 probes, the gene name, Unigene, fold change, and p-value. The corresponding text in the Results section has also been updated to explicitly refer to S1 Table instead of "data not shown." (line: 219)

We hope that this supplementary table sufficiently addresses the reviewer's request for access to the underlying data.

5. Need more clarity on the sample size and availability of the statistical analysis data.

We appreciate the reviewer's comment regarding the need for clearer information on sample size and statistical analyses. In the revised manuscript, we have clarified the sample size (n) and statistical methods as follows.

•For the DNA microarray analysis, we now specify that "the CEL files obtained from three independent samples per condition (n = 3)". (line: 126-127)

•For the gene expression and western blotting experiments, we now state that " Three replicate wells were evaluated for each group (n = 3)" in the Materials and Methods sections (line: 159, 170, 193).

•The figure legends (e.g., Fig 2 (line: 247-254)) have been revised to indicate the statistical test used (Dunnett's test) and the significance thresholds, together with the sample size (n = 3).

•We have revised the Data Availability statement in the manuscript as follows:

"All microarray data files have been deposited in the NCBI Gene Expression Omnibus (GEO) under accession number **GSEXXXXXXX (to be updated once the accession number is assigned)**. All other datasets used for the statistical analyses, including individual data points and sample sizes for each experiment, have been deposited in the Zenodo repository and are freely available under the accession DOI 10.5281/zenodo.19227766. All other relevant data are within the manuscript and its Supporting Information files." (line: 372-380)

We hope that this revision sufficiently clarifies the sample sizes and the accessibility of all datasets used in our analyses.

Reviewer #3

1. The bar graphs are not properly labeled on the x-axis, and statistical significance is not indicated.
We appreciate the reviewer's valuable comment regarding the x-axis labels and the indication of statistical significance.

In response, we have revised the figures as follows:

Fig 2: We performed a Dunnett's multiple comparison test and have now indicated statistically significant differences on the graph. In addition, we adjusted the positions of the x-axis labels to improve clarity and readability.

Fig 3: We conducted a Student's t-test and have added the corresponding statistical significance to the graph. Similarly, we optimized the placement of the x-axis labels in this figure.

We believe these revisions address the reviewer's concerns and improve the quality and interpretability of the figures.
2. The authors claim that maltotetraose is a promising ingredient for cosmetic applications with potential roles in improving skin health, reducing pigmentation, and combating aging. However, no experimental data are provided to support claims related to pigmentation reduction or anti-aging effects. Moreover, the experiments were conducted exclusively in keratinocytes. The role of dermal fibroblasts, which are critical for maintaining skin elasticity and extracellular matrix integrity, has not been addressed.
We appreciate this important comment and have revised the Discussion to avoid overstatement and to clarify the limitations of our study design. We now explicitly acknowledge that our data are restricted to keratinocyte responses and do not include fibroblast or pigmentation endpoints. The following statements were added:

"Given that excessive ROS are known to contribute to collagen and elastin degradation and to hyperpigmentation through the activation of melanocyte-stimulation factors [16-19], NRF2 activation and ROS reduction by hydrolyzed corn starch raise the possibility that this ingredient may indirectly influence skin conditions such as wrinkles, sagging, and pigmentation. However, our current data are limited to antioxidant responses in keratinocytes, and we did not directly assess structural or functional endpoints (e.g., collagen content, skin elasticity, melanin production) in the skin. Therefore, any anti-wrinkle, anti-sagging, or depigmenting effects remain speculative and should be validated in future in vitro and clinical studies. In addition,

our experiments were conducted exclusively in keratinocytes and did not address the role of dermal fibroblasts, which are crucial for extracellular matrix production and skin elasticity. Future investigations using dermal fibroblasts, melanocyte-keratinocyte co-culture systems, and in vivo or clinical evaluations will be required to clarify whether the NRF2 activation and ROS reduction observed here translate into measurable improvements in these skin conditions." (line: 321-332)

Furthermore, we have revised the description of maltotetraose (MTO) so that it is no longer presented as a definitively "promising" cosmetic ingredient, but rather positioned as a mechanistic hypothesis based on the current data.

"Our results also suggest that maltotetraose (MTO), a major component of hydrolyzed corn starch, may play a key role in NRF2 activation and subsequent ROS reduction in keratinocytes, although the detailed mechanism remains unclear. Whether ROS reduction is solely mediated by the NRF2 pathway and the specific mechanism by which MTO activates NRF2 are not yet established. In addition, we did not evaluate the overall status of enzymatic and non-enzymatic antioxidant capacity in this study, and thus the global impact of MTO on the cutaneous antioxidant defense system remains unclear. A comprehensive understanding will require the identification of the individual compounds in the hydrolyzed corn starch, subsequent validation using each individual compound, and elucidation of the associated molecular interactions and signaling pathways with specific inhibitors." (line: 350-359)

3. The discussion section requires substantial improvement to better interpret the findings and place them in the context of existing literature.

We have reorganized the Discussion to follow a more logical progression:

(1) overview of NRF2–ARE signaling and ROS biology in the skin

(2) summary of our key findings (NRF2 activation, gene expression, and ROS reduction in keratinocytes)

(3) additional findings (expression of other antioxidant related genes)

(4) cautious discussion of potential implications for skin aging and pigmentation, explicitly framed as hypotheses

(5) clarification of limitations, including the lack of data on fibroblasts, extracellular matrix, pigmentation, and inflammatory markers

(6) future directions, including the need for compound-level analyses, mechanistic studies with inhibitors, and clinical validation.

4. The authors evaluated NRF2-associated antioxidant response genes, including HMOX1, GPX2, and NQO1. However, the effects of maltotetraose on other antioxidant systems, such as PRDX1, as well as overall enzymatic and non-enzymatic antioxidant status, were not investigated.

We appreciate the reviewer's suggestion to further investigate the broader antioxidant system beyond NRF2-related genes such as *HMOX1*, *GPX2*, and *NQO1*. In response, we performed additional analyses of antioxidant-related gene expression. Specifically, we quantified the mRNA expression levels of peroxiredoxin 1 (*PRDX1*), superoxide dismutase 1 (*SOD1*), catalase (*CAT*), glutathione reductase (*GSR*), glutamate-cysteine ligase catalytic subunit (*GCLC*), and thioredoxin (*TXN*) by RT-qPCR after treatment with 1.0%, 2.0%, and 4.0% hydrolyzed corn starch for 24 h. As shown in the revised Results section and Fig. S1, *GCLC* and *TXN* were upregulated in a concentration-dependent manner, and *PRDX1* was significantly increased at 4.0%, whereas *SOD1*, *CAT*, and *GSR* showed no significant changes.

These findings have been incorporated into the Results (line: 238-244) and discussed in the Discussion section (line: 311-320). In addition, we explicitly noted in the revised Discussion that "we did not evaluate the overall status of enzymatic and non-enzymatic antioxidant capacity in this study, and thus the global impact of maltotetraose on the cutaneous antioxidant defense system remains unclear." (line: 354-356)
