# Supplementary material for: Hydrolyzed corn starch with maltotetraose for skin defense through NRF2 pathway activation in human keratinocytes
Source: PLoS One. 2026 Jun 26;21(6):e0351422. doi: 10.1371/journal.pone.0351422 (PMC13309040; doi:10.1371/journal.pone.0351422)
Supplement: Peer review history

# Peer review: Decision Letter 0

**DOI:** 10.1371/journal.pone.0351422.r001

**Authors:** Ahmed Abdel Moneim

12 Feb 2026

PONE-D-25-63339 Hydrolyzed corn starch with maltotetraose for skin defense through NRF2 pathway activation in human keratinocytes PLOS One

Dear Dr. Hagawa,

Thank you for submitting your manuscript to PLOS ONE. After careful consideration, we feel that it has merit but does not fully meet PLOS ONE’s publication criteria as it currently stands. Therefore, we invite you to submit a revised version of the manuscript that addresses the points raised during the review process.

Please submit your revised manuscript by Mar 29 2026 11:59PM. If you will need more time than this to complete your revisions, please reply to this message or contact the journal office at plosone@plos.org. When you're ready to submit your revision, log on to https://www.editorialmanager.com/pone/ and select the 'Submissions Needing Revision' folder to locate your manuscript file.

Please include the following items when submitting your revised manuscript:

- A letter that responds to each point raised by the academic editor and reviewer(s). You should upload this letter as a separate file labeled 'Response to Reviewers'.
- A marked-up copy of your manuscript that highlights changes made to the original version. You should upload this as a separate file labeled 'Revised Manuscript with Track Changes'.
- An unmarked version of your revised paper without tracked changes. You should upload this as a separate file labeled 'Manuscript'.

If you would like to make changes to your financial disclosure, please include your updated statement in your cover letter. Guidelines for resubmitting your figure files are available below the reviewer comments at the end of this letter.

If applicable, we recommend that you deposit your laboratory protocols in protocols.io to enhance the reproducibility of your results. Protocols.io assigns your protocol its own identifier (DOI) so that it can be cited independently in the future. For instructions see: https://journals.plos.org/plosone/s/submission-guidelines#loc-laboratory-protocols. Additionally, PLOS ONE offers an option for publishing peer-reviewed Lab Protocol articles, which describe protocols hosted on protocols.io. Read more information on sharing protocols at https://plos.org/protocols?utm_medium=editorial-email&utm_source=authorletters&utm_campaign=protocols.

We look forward to receiving your revised manuscript.

Kind regards,

Ahmed E. Abdel Moneim

Academic Editor

PLOS One

Journal Requirements:

When submitting your revision, we need you to address these additional requirements.

1. Please ensure that your manuscript meets PLOS ONE's style requirements, including those for file naming. The PLOS ONE style templates can be found at

https://journals.plos.org/plosone/s/file?id=wjVg/PLOSOne_formatting_sample_main_body.pdf and

https://journals.plos.org/plosone/s/file?id=ba62/PLOSOne_formatting_sample_title_authors_affiliations.pdf.

2. Thank you for stating in your Funding Statement:

“This research was supported financially by the Lion Corporation. No other specific grant was received from funding agencies in the public, commercial, or not-for-profit sectors.”

Please provide an amended statement that declares *all* the funding or sources of support (whether external or internal to your organization) received during this study, as detailed online in our guide for authors at http://journals.plos.org/plosone/s/submit-now.  Please also include the statement “There was no additional external funding received for this study.” in your updated Funding Statement.

Please include your amended Funding Statement within your cover letter. We will change the online submission form on your behalf.

3. Thank you for stating the following financial disclosure:

“This research was supported financially by the Lion Corporation. No other specific grant was received from funding agencies in the public, commercial, or not-for-profit sectors.”

Please state what role the funders took in the study.  If the funders had no role, please state: "The funders had no role in study design, data collection and analysis, decision to publish, or preparation of the manuscript."

If this statement is not correct you must amend it as needed.

Please include this amended Role of Funder statement in your cover letter; we will change the online submission form on your behalf.

4. We note that you have indicated that there are restrictions to data sharing for this study. For studies involving human research participant data or other sensitive data, we encourage authors to share de-identified or anonymized data. However, when data cannot be publicly shared for ethical reasons, we allow authors to make their data sets available upon request. For information on unacceptable data access restrictions, please see http://journals.plos.org/plosone/s/data-availability#loc-unacceptable-data-access-restrictions.

Before we proceed with your manuscript, please address the following prompts:

a) If there are ethical or legal restrictions on sharing a de-identified data set, please explain them in detail (e.g., data contain potentially identifying or sensitive patient information, data are owned by a third-party organization, etc.) and who has imposed them (e.g., a Research Ethics Committee or Institutional Review Board, etc.). Please also provide contact information for a data access committee, ethics committee, or other institutional body to which data requests may be sent.

b) If there are no restrictions, please upload the minimal anonymized data set necessary to replicate your study findings to a stable, public repository and provide us with the relevant URLs, DOIs, or accession numbers. Please see http://www.bmj.com/content/340/bmj.c181.long for guidelines on how to de-identify and prepare clinical data for publication. For a list of recommended repositories, please see https://journals.plos.org/plosone/s/recommended-repositories. You also have the option of uploading the data as Supporting Information files, but we would recommend depositing data directly to a data repository if possible.

Please update your Data Availability statement in the submission form accordingly.

5. In the online submission form you indicate that your data is not available for proprietary reasons and have provided a contact point for accessing this data. Please note that your current contact point is a co-author on this manuscript. According to our Data Policy, the contact point must not be an author on the manuscript and must be an institutional contact, ideally not an individual. Please revise your data statement to a non-author institutional point of contact, such as a data access or ethics committee, and send this to us via return email. Please also include contact information for the third party organization, and please include the full citation of where the data can be found.

6. Please be informed that funding information should not appear in the Acknowledgments section or other areas of your manuscript. We will only publish funding information present in the Funding Statement section of the online submission form. Please remove any funding-related text from the manuscript.

7. We note that you have included the phrase “data not shown” in your manuscript. Unfortunately, this does not meet our data sharing requirements. PLOS does not permit references to inaccessible data. We require that authors provide all relevant data within the paper, Supporting Information files, or in an acceptable, public repository. Please add a citation to support this phrase or upload the data that corresponds with these findings to a stable repository (such as Figshare or Dryad) and provide and URLs, DOIs, or accession numbers that may be used to access these data. Or, if the data are not a core part of the research being presented in your study, we ask that you remove the phrase that refers to these data.

8. PLOS ONE now requires that authors provide the original uncropped and unadjusted images underlying all blot or gel results reported in a submission’s figures or Supporting Information files. This policy and the journal’s other requirements for blot/gel reporting and figure preparation are described in detail at https://journals.plos.org/plosone/s/figures#loc-blot-and-gel-reporting-requirements and https://journals.plos.org/plosone/s/figures#loc-preparing-figures-from-image-files. When you submit your revised manuscript, please ensure that your figures adhere fully to these guidelines and provide the original underlying images for all blot or gel data reported in your submission. See the following link for instructions on providing the original image data: https://journals.plos.org/plosone/s/figures#loc-original-images-for-blots-and-gels.

In your cover letter, please note whether your blot/gel image data are in Supporting Information or posted at a public data repository, provide the repository URL if relevant, and provide specific details as to which raw blot/gel images, if any, are not available. Email us at plosone@plos.org if you have any questions.

9. If the reviewer comments include a recommendation to cite specific previously published works, please review and evaluate these publications to determine whether they are relevant and should be cited. There is no requirement to cite these works unless the editor has indicated otherwise.

[Note: HTML markup is below. Please do not edit.]

Reviewers' comments:

Reviewer's Responses to Questions

**Comments to the Author**

1. Is the manuscript technically sound, and do the data support the conclusions?

The manuscript must describe a technically sound piece of scientific research with data that supports the conclusions. Experiments must have been conducted rigorously, with appropriate controls, replication, and sample sizes. The conclusions must be drawn appropriately based on the data presented.

Reviewer #1: Partly

Reviewer #2: Yes

Reviewer #3: No

**********

2. Has the statistical analysis been performed appropriately and rigorously?

Reviewer #1: Yes

Reviewer #2: Yes

Reviewer #3: No

**********

3. Have the authors made all data underlying the findings in their manuscript fully available?

The PLOS Data policy requires authors to make all data underlying the findings described in their manuscript fully available without restriction, with rare exception (please refer to the Data Availability Statement in the manuscript PDF file). The data should be provided as part of the manuscript or its supporting information, or deposited to a public repository. For example, in addition to summary statistics, the data points behind means, medians and variance measures should be available. If there are restrictions on publicly sharing data—e.g. participant privacy or use of data from a third party—those must be specified.

Reviewer #1: Yes

Reviewer #2: No

Reviewer #3: No

**********

4. Is the manuscript presented in an intelligible fashion and written in standard English?

PLOS ONE does not copyedit accepted manuscripts, so the language in submitted articles must be clear, correct, and unambiguous. Any typographical or grammatical errors should be corrected at revision, so please note any specific errors here.

Reviewer #1: Yes

Reviewer #2: Yes

Reviewer #3: No

**********

5. Review Comments to the Author

Please use the space provided to explain your answers to the questions above. You may also include additional comments for the author, including concerns about dual publication, research ethics, or publication ethics. (Please upload your review as an attachment if it exceeds 20,000 characters)

Reviewer #1: Comments to the authors:

1. Repetition of the phrase “hydrolyzed corn starch containing maltotetraose” affects flow and should be reduced in the Abstract section.

2. No pigmentation-related markers, melanocyte models, or aging-associated endpoints are mentioned.

3. In line 32, the phrase “novel functionality” is vague and should be replaced with more precise scientific language.

4. Typographical error identified: “maltotetoraose” should be corrected to “maltotetraose” in line 33.

5. In line 56, the statement that NRF2 is a “promising target in cosmetic applications” is plausible but under-argued. Clarify why moderate NRF2 activation is beneficial and safe in cosmetic formulations.

6. “Raw and functional cosmetic ingredient” is vague. Consider replacing with “naturally derived functional cosmetic ingredient” in line 57.

7. The assertion that MTO has “superior skin-moisturizing properties” based on hydroxyl group content alone is chemically simplistic. Provide experimental or literature-based evidence supporting superior humectancy, or rephrase to a more cautious claim (e.g., “potentially enhanced moisturizing capacity”) in line 60.

8. In line 184, the term “robes” appears to be a typographical error and should be corrected to “probes”. The phrase “data not shown” is not acceptable for a primary result in a manuscript. Transparency requires that the underlying data be accessible.

9. Reporting only the number of selected probes (n = 73) without presenting any supporting data (e.g., fold changes, adjusted p-values, or gene identities) is insufficient. At a minimum, a supplementary table should be provided.

10. The discussion appropriately outlines the NRF2–ARE antioxidant pathway and its relevance to skin oxidative stress. However, the conclusion that hydrolyzed corn starch mitigates wrinkles, sagging, and pigmentation is speculative and not directly supported by the presented data.

11. Justify this statement: the anti-inflammatory effect is inferred solely from literature citing NRF2-mediated suppression of IL-6 and IL-1β. Since no inflammatory markers were measured in this study, this conclusion should be framed as a potential implication, not as an observed effect in lines 265-268.

Reviewer #2: It’s an interesting piece of work which should be corroborated with human trials as early as possible.

1. Please provide latest references for the second paragraph of the Introduction section.

2. If possible please provide a schematic to explain the pathways during presence and absence of oxidative stress.

3. Is there previously available literature that supports hydrolyzed corn starch or MTO f as a potent cosmetic ingredient?

4. The data of 73 probes can be provided as supplementary information?

5. Need more clarity on the sample size and availability of the statistical analysis data.

Reviewer #3: Comments to the authors

The author investigated the potential of hydrolyzed corn starch containing maltotetraose in skin defense through nuclear factor erythroid 2- related factor 2 (NRF2) pathway activation in human keratinocytes exposed to oxidative stress. They have checked the hydrolyzed corn starch on antioxidant-responsive genes, including HMOX1, GPX2, and NQO1, which are known targets of NRF2 human keratinocytes cells. The manuscript lacks rigor in several key aspects, including statistical analysis, experimental depth, novelty, and the discussion. Therefore, I recommend rejection of the manuscript in its current form.

1. The bar graphs are not properly labeled on the x-axis, and statistical significance is not indicated.

2. The authors claim that maltotetraose is a promising ingredient for cosmetic applications with potential roles in improving skin health, reducing pigmentation, and combating aging. However, no experimental data are provided to support claims related to pigmentation reduction or anti-aging effects. Moreover, the experiments were conducted exclusively in keratinocytes. The role of dermal fibroblasts, which are critical for maintaining skin elasticity and extracellular matrix integrity, has not been addressed.

3. The discussion section requires substantial improvement to better interpret the findings and place them in the context of existing literature.

4. The authors evaluated NRF2-associated antioxidant response genes, including HMOX1, GPX2, and NQO1. However, the effects of maltotetraose on other antioxidant systems, such as PRDX1, as well as overall enzymatic and non-enzymatic antioxidant status, were not investigated.

**********

6. PLOS authors have the option to publish the peer review history of their article (what does this mean?). If published, this will include your full peer review and any attached files.

If you choose “no”, your identity will remain anonymous but your review may still be made public.

**Do you want your identity to be public for this peer review?** For information about this choice, including consent withdrawal, please see our Privacy Policy.

Reviewer #1: No

Reviewer #2: **Yes:** Dr. Gauri Awasthi

Reviewer #3: **Yes:** Agilan Balupillai

**********

[NOTE: If reviewer comments were submitted as an attachment file, they will be attached to this email and accessible via the submission site. Please log into your account, locate the manuscript record, and check for the action link "View Attachments". If this link does not appear, there are no attachment files.]

To ensure your figures meet our technical requirements, please review our figure guidelines: https://journals.plos.org/plosone/s/figures

You may also use PLOS’s free figure tool, NAAS, to help you prepare publication quality figures: https://journals.plos.org/plosone/s/figures#loc-tools-for-figure-preparation.

NAAS will assess whether your figures meet our technical requirements by comparing each figure against our figure specifications.

# Author comment: Author response to Decision Letter 1

**DOI:** 10.1371/journal.pone.0351422.r002

29 Mar 2026

Journal Requirement (Editor)

1. Please ensure that your manuscript meets PLOS ONE's style requirements, including those for file naming. The PLOS ONE style templates can be found at https://journals.plos.org/plosone/s/file?id=wjVg/PLOSOne_formatting_sample_main_body.pdf and https://journals.plos.org/plosone/s/file?id=ba62/PLOSOne_formatting_sample_title_authors_affiliations.pdf.

>We have carefully revised the manuscript to ensure that it follows the PLOS ONE style requirements, including those for file naming and formatting. We have consulted and applied the formatting guidelines provided in the PLOS ONE style templates.

2. Thank you for stating in your Funding Statement: This research was supported financially by the Lion Corporation. No other specific grant was received from funding agencies in the public, commercial, or not-for-profit sectors.” Please provide an amended statement that declares *all* the funding or sources of support (whether external or internal to your organization) received during this study, as detailed online in our guide for authors at http://journals.plos.org/plosone/s/submit-now. Please also include the statement “There was no additional external funding received for this study.” in your updated Funding Statement.

Please include your amended Funding Statement within your cover letter. We will change the online submission form on your behalf.

>We appreciate the editor’s guidance regarding the Funding Statement. We have revised the Funding Statement to read as follows, and will include this updated version in the cover letter:“This research was supported financially by the Lion Corporation. No other specific grant was received from funding agencies in the public, commercial, or not-for-profit sectors. There was no additional external funding received for this study.”

We understand that the online submission form will be updated by the editorial office based on the text provided in the cover letter.

3. Thank you for stating the following financial disclosure:

“This research was supported financially by the Lion Corporation. No other specific grant was received from funding agencies in the public, commercial, or not-for-profit sectors.” Please state what role the funders took in the study. If the funders had no role, please state: "The funders had no role in study design, data collection and analysis, decision to publish, or preparation of the manuscript." If this statement is not correct you must amend it as needed. Please include this amended Role of Funder statement in your cover letter; we will change the online submission form on your behalf.

>We thank the editor for pointing out the need to clarify the role of the funder.

In accordance with the journal’s request, we have added the following statement regarding the role of the funder, which will be included in the cover letter:

"The funders had no role in study design, data collection and analysis, decision to publish, or preparation of the manuscript."

We understand that the editorial office will update the corresponding section in the online submission system based on this statement.

4. We note that you have indicated that there are restrictions to data sharing for this study. For studies involving human research participant data or other sensitive data, we encourage authors to share de-identified or anonymized data. However, when data cannot be publicly shared for ethical reasons, we allow authors to make their data sets available upon request. For information on unacceptable data access restrictions, please see http://journals.plos.org/plosone/s/data-availability#loc-unacceptable-data-access-restrictions. Before we proceed with your manuscript, please address the following prompts:

a) If there are ethical or legal restrictions on sharing a de-identified data set, please explain them in detail (e.g., data contain potentially identifying or sensitive patient information, data are owned by a third-party organization, etc.) and who has imposed them (e.g., a Research Ethics Committee or Institutional Review Board, etc.). Please also provide contact information for a data access committee, ethics committee, or other institutional body to which data requests may be sent.

b) If there are no restrictions, please upload the minimal anonymized data set necessary to replicate your study findings to a stable, public repository and provide us with the relevant URLs, DOIs, or accession numbers. Please see http://www.bmj.com/content/340/bmj.c181.long for guidelines on how to de-identify and prepare clinical data for publication. For a list of recommended repositories, please see https://journals.plos.org/plosone/s/recommended-repositories. You also have the option of uploading the data as Supporting Information files, but we would recommend depositing data directly to a data repository if possible.

Please update your Data Availability statement in the submission form accordingly.

>We have revised the Data Availability statement in the manuscript to more clearly describe where the data can be accessed. The revised statement reads as follows: However, the microarray data have not yet been assigned an accession number in GEO. The revised statement reads as follows:

“All microarray data files have been deposited in the NCBI Gene Expression Omnibus (GEO) under accession number GSEXXXXXX (to be updated once the accession number is assigned). All other datasets used for the statistical analyses, including individual data points and sample sizes for each experiment, have been deposited in the Zenodo repository and are freely available under the accession DOI 10.5281/zenodo.19227766. All other relevant data are within the manuscript and its Supporting Information files.” (line 372–380)

We hope that this revision sufficiently clarifies the sample sizes and the accessibility of all datasets used in our analyses and ensures compliance with the PLOS ONE Data Availability policy.

5. In the online submission form you indicate that your data is not available for proprietary reasons and have provided a contact point for accessing this data. Please note that your current contact point is a co-author on this manuscript. According to our Data Policy, the contact point must not be an author on the manuscript and must be an institutional contact, ideally not an individual. Please revise your data statement to a non-author institutional point of contact, such as a data access or ethics committee, and send this to us via return email. Please also include contact information for the third party organization, and please include the full citation of where the data can be found.

>In response to the journal’s data policy, we have revised our data sharing plan so that the underlying datasets are now publicly available and no longer subject to proprietary access restrictions.

As noted above, the microarray data have been submitted to the NCBI Gene Expression Omnibus (GEO) and will be available under accession GSEXXXXXX once it is assigned. All other datasets used for statistical analyses have been deposited in Zenodo (DOI 10.5281/zenodo.19227766). Therefore, we no longer rely on a “data available upon request” model, and no author will serve as a direct data-access contact point.

Because the data are openly available through these public repositories, an additional institutional contact point for data access is no longer required.

6. Please be informed that funding information should not appear in the Acknowledgments section or other areas of your manuscript. We will only publish funding information present in the Funding Statement section of the online submission form. Please remove any funding-related text from the manuscript.

>We acknowledge the journal’s policy that funding information should only appear in the Funding Statement section of the online submission form and not in the Acknowledgments or other parts of the manuscript. Accordingly, we have removed all funding-related text from the Acknowledgments section and from any other location in the manuscript.

7. We note that you have included the phrase “data not shown” in your manuscript. Unfortunately, this does not meet our data sharing requirements. PLOS does not permit references to inaccessible data. We require that authors provide all relevant data within the paper, Supporting Information files, or in an acceptable, public repository. Please add a citation to support this phrase or upload the data that corresponds with these findings to a stable repository (such as Figshare or Dryad) and provide and URLs, DOIs, or accession numbers that may be used to access these data. Or, if the data are not a core part of the research being presented in your study, we ask that you remove the phrase that refers to these data.

>We thank the editor and reviewers for drawing our attention to the use of the phrase “data not shown” and for emphasizing the importance of data transparency. In response to the concern that “data not shown” is not acceptable for key results and that the underlying data must be available, we have now provided the supporting microarray data for the 73 selected probes as a supplementary file (S1 Table). These changes ensure that all relevant data supporting our findings are accessible to readers and fully comply with PLOS ONE’s data sharing requirements.

8. PLOS ONE now requires that authors provide the original uncropped and unadjusted images underlying all blot or gel results reported in a submission’s figures or Supporting Information files. This policy and the journal’s other requirements for blot/gel reporting and figure preparation are described in detail at https://journals.plos.org/plosone/s/figures#loc-blot-and-gel-reporting-requirements and https://journals.plos.org/plosone/s/figures#loc-preparing-figures-from-image-files. When you submit your revised manuscript, please ensure that your figures adhere fully to these guidelines and provide the original underlying images for all blot or gel data reported in your submission. See the following link for instructions on providing the original image data: https://journals.plos.org/plosone/s/figures#loc-original-images-for-blots-and-gels. In your cover letter, please note whether your blot/gel image data are in Supporting Information or posted at a public data repository, provide the repository URL if relevant, and provide specific details as to which raw blot/gel images, if any, are not available. Email us at plosone@plos.org if you have any questions.

>We understand and appreciate the journal’s policy requiring the submission of original, uncropped, and unadjusted images for all blot and gel data. In accordance with this policy, we have prepared and submitted the raw Western blot images underlying Figure 4 as Supporting information (S1_raw_images.pdf). These files correspond directly to the processed images shown in Figure 4. We will clearly indicate in the cover letter that the raw blot images for Figure 4 have been provided, and we confirm that there are no additional blot or gel images in the manuscript for which raw data are unavailable.

9. If the reviewer comments include a recommendation to cite specific previously published works, please review and evaluate these publications to determine whether they are relevant and should be cited. There is no requirement to cite these works unless the editor has indicated otherwise.

>We note that the reviewers did not explicitly recommend any specific previously published works that must be cited in this manuscript. Accordingly, no reviewer-suggested citations needed to be added. We have, however, reviewed and updated the references in the Introduction and Discussion where appropriate to ensure that the most relevant and recent literature is cited.

Reviewer #1

1. Repetition of the phrase “hydrolyzed corn starch containing maltotetraose” affects flow and should be reduced in the Abstract section.

>We appreciate this helpful comment. In the revised Abstract, we now use the full expression “hydrolyzed corn starch containing maltotetraose” only when the ingredient is first introduced and again in the concluding sentence where we summarize its significance. In the rest of the Abstract, we refer to it more concisely as “this ingredient (line: 24 and 32),” “it,” (line: 25) or “the ingredient” (line: 29-30) where the meaning is clear. We believe this has reduced redundancy and improved the readability of the Abstract.

2. No pigmentation-related markers, melanocyte models, or aging-associated endpoints are mentioned.

>We appreciate insightful comments. We fully agree that our current study did not include pigmentation-related markers, melanocyte-based models, or aging-associated endpoints, and that any statements regarding pigmentation or aging must therefore be made with caution.

In response to this comment, we have revised the Abstract and Discussion to clearly state that the data presented in this study are limited to antioxidant responses and NRF2 activation in keratinocytes. Any potential implications for pigmentation or aging are now explicitly described as hypothetical and not directly evaluated in this work. In the Abstract we now state:

“This NRF2-mediated antioxidative activity positions hydrolyzed corn starch containing maltotetraose as a promising ingredient for cosmetic products, with potential implications for improving skin health, reducing pigmentation, and combating aging. However, these effects were not directly evaluated in this study. Future studies are warranted to evaluate its efficacy in vivo.” (line: 33-38)

In the Discussion, we also note that:

“However, our current data are limited to antioxidant responses in keratinocytes, and we did not directly assess structural or functional endpoints (e.g., collagen content, skin elasticity, melanin production) in the skin. Therefore, any anti-wrinkle, anti-sagging, or depigmenting effects remain speculative and should be validated in future in vitro and clinical studies.” (line: 324-328)

We hope that these revisions clarify the scope and limitations of our study.

3. In line 32, the phrase “novel functionality” is vague and should be replaced with more precise scientific language.

>We appreciate this helpful comment. We agree that the term “novel functionality” was vague. In the revised manuscript, we have replaced this phrase with the more specific and scientifically accurate term “NRF2-mediated antioxidative activity,” (line: 34) which directly reflects the mechanism demonstrated in our study. The sentence now reads:

“This NRF2-mediated antioxidative activity positions hydrolyzed corn starch containing maltotetraose as a promising ingredient for cosmetic products, with potential implications for improving skin health, reducing pigmentation, and combating aging.” (line: 33-36)

This revision clarifies that the functionality in question is the activation of the NRF2 pathway leading to an enhanced antioxidative response.

4. Typographical error identified: “maltotetoraose” should be corrected to “maltotetraose” in line 33.

>We thank the reviewer for pointing out this typographical error. In the revised manuscript, we have corrected the misspelling “maltotetoraose” to “maltotetraose” on line 35. We have also checked the entire manuscript to ensure that the term “maltotetraose” is spelled consistently throughout.

5. In line 56, the statement that NRF2 is a “promising target in cosmetic applications” is plausible but under-argued. Clarify why moderate NRF2 activation is beneficial and safe in cosmetic formulations.

>We appreciate this valuable comment. We agree that additional explanation was needed to clarify why NRF2 can be regarded as a beneficial and safe target in the context of cosmetic formulations.

In the revised Introduction, we have elaborated on the role of NRF2 in skin and the rationale for targeting it in cosmetics. (line: 59-67) Specifically, we now emphasize that:

•NRF2 regulates a broad range of antioxidant and phase II detoxification enzymes that detoxify and eliminate reactive oxygen species and other oxidative agents, thereby contributing to photoprotection, regulation of pigmentation, wound healing, and reinforcement of epidermal barrier function [3,5-7]. Enhancement of NRF2 signaling is therefore expected to strengthen endogenous antioxidan

Attachment Submitted filename: Response to Reviewers.pdf

# Peer review: Decision Letter 1

**DOI:** 10.1371/journal.pone.0351422.r003

**Authors:** Ahmed Abdel Moneim, Ahmed Abdel Moneim

27 May 2026

Hydrolyzed corn starch with maltotetraose for skin defense through NRF2 pathway activation in human keratinocytes

PONE-D-25-63339R1

Dear Dr. Hagawa,

We’re pleased to inform you that your manuscript has been judged scientifically suitable for publication and will be formally accepted for publication once it meets all outstanding technical requirements.

Within one week, you’ll receive an e-mail detailing the required amendments. When these have been addressed, you’ll receive a formal acceptance letter and your manuscript will be scheduled for publication.

An invoice will be generated when your article is formally accepted. Please note, if your institution has a publishing partnership with PLOS and your article meets the relevant criteria, all or part of your publication costs will be covered. Please make sure your user information is up-to-date by logging into Editorial Manager at Editorial Manager® and clicking the ‘Update My Information' link at the top of the page. For questions related to billing, please contact billing support.

If your institution or institutions have a press office, please notify them about your upcoming paper to help maximize its impact. If they’ll be preparing press materials, please inform our press team as soon as possible -- no later than 48 hours after receiving the formal acceptance. Your manuscript will remain under strict press embargo until 2 pm Eastern Time on the date of publication. For more information, please contact onepress@plos.org.

Kind regards,

Ahmed E. Abdel Moneim

Academic Editor

PLOS One

Additional Editor Comments (optional):

Reviewers' comments:

Reviewer's Responses to Questions

**Comments to the Author**

1. If the authors have adequately addressed your comments raised in a previous round of review and you feel that this manuscript is now acceptable for publication, you may indicate that here to bypass the “Comments to the Author” section, enter your conflict of interest statement in the “Confidential to Editor” section, and submit your "Accept" recommendation.

Reviewer #2: All comments have been addressed

Reviewer #4: All comments have been addressed

**********

2. Is the manuscript technically sound, and do the data support the conclusions?

The manuscript must describe a technically sound piece of scientific research with data that supports the conclusions. Experiments must have been conducted rigorously, with appropriate controls, replication, and sample sizes. The conclusions must be drawn appropriately based on the data presented.

Reviewer #2: Yes

Reviewer #4: Yes

**********

3. Has the statistical analysis been performed appropriately and rigorously?

Reviewer #2: Yes

Reviewer #4: Yes

**********

4. Have the authors made all data underlying the findings in their manuscript fully available?

The PLOS Data policy requires authors to make all data underlying the findings described in their manuscript fully available without restriction, with rare exception (please refer to the Data Availability Statement in the manuscript PDF file). The data should be provided as part of the manuscript or its supporting information, or deposited to a public repository. For example, in addition to summary statistics, the data points behind means, medians and variance measures should be available. If there are restrictions on publicly sharing data—e.g. participant privacy or use of data from a third party—those must be specified.

Reviewer #2: Yes

Reviewer #4: Yes

**********

5. Is the manuscript presented in an intelligible fashion and written in standard English?

PLOS ONE does not copyedit accepted manuscripts, so the language in submitted articles must be clear, correct, and unambiguous. Any typographical or grammatical errors should be corrected at revision, so please note any specific errors here.

Reviewer #2: Yes

Reviewer #4: Yes

**********

6. Review Comments to the Author

Please use the space provided to explain your answers to the questions above. You may also include additional comments for the author, including concerns about dual publication, research ethics, or publication ethics. (Please upload your review as an attachment if it exceeds 20,000 characters)

Reviewer #2: The authors have addressed all the comments according to the authors satisfaction including the schematic figure and data relevant to the study hence the manuscript should be considered for publication.

Reviewer #4: An important point to be considered for future studies shall be the qualitative analysis of the corn starch hydrolysate, particularly for contents of maltotetraose and other relevant components, as the compositions can vary from batch to batch.

**********

7. PLOS authors have the option to publish the peer review history of their article (what does this mean?). If published, this will include your full peer review and any attached files.

If you choose “no”, your identity will remain anonymous but your review may still be made public.

**Do you want your identity to be public for this peer review?** For information about this choice, including consent withdrawal, please see our Privacy Policy.

Reviewer #2: **Yes:** Dr. Gauri Awasthi

Reviewer #4: **Yes:** Muhammad Waheed Akhtar

**********

Attachment Submitted filename: PONE-D-25-63339_R1.pdf

# Editor report: Acceptance letter

**DOI:** 10.1371/journal.pone.0351422.r004

**Authors:** Ahmed Abdel Moneim, Ahmed Abdel Moneim

PONE-D-25-63339R1

PLOS One

Dear Dr. Hagawa,

I'm pleased to inform you that your manuscript has been deemed suitable for publication in PLOS One. Congratulations! Your manuscript is now being handed over to our production team.

At this stage, our production department will prepare your paper for publication. This includes ensuring the following:

* All references, tables, and figures are properly cited

* All relevant supporting information is included in the manuscript submission,

* There are no issues that prevent the paper from being properly typeset

You will receive further instructions from the production team, including instructions on how to review your proof when it is ready. Please keep in mind that we are working through a large volume of accepted articles, so please give us a few days to review your paper and let you know the next and final steps.

Lastly, if your institution or institutions have a press office, please let them know about your upcoming paper now to help maximize its impact. If they'll be preparing press materials, please inform our press team within the next 48 hours. Your manuscript will remain under strict press embargo until 2 pm Eastern Time on the date of publication. For more information, please contact onepress@plos.org.

You will receive an invoice from PLOS for your publication fee after your manuscript has reached the completed accept phase. If you receive an email requesting payment before acceptance or for any other service, this may be a phishing scheme. Learn how to identify phishing emails and protect your accounts at https://explore.plos.org/phishing.

If we can help with anything else, please email us at customercare@plos.org.

Thank you for submitting your work to PLOS ONE and supporting open access.

Kind regards,

PLOS ONE Editorial Office Staff

on behalf of

Dr. Ahmed E. Abdel Moneim

Academic Editor

PLOS One
